# Comparative Analysis of Evolutionary Distances Using the Genus *Mycobacterium*

**DOI:** 10.3390/ijms262110471

**Published:** 2025-10-28

**Authors:** Danila Zimenkov, Anastasia Ushtanit

**Affiliations:** Center for High-Tech Bioeconomy, Engelhardt Institute of Molecular Biology, Russian Academy of Sciences, 119991 Moscow, Russia

**Keywords:** *Mycobacterium*, phylogenomics, cluster analysis, phylogenetic tree, AAI

## Abstract

Infections caused by nontuberculous mycobacteria are becoming significant due to the increasing number of vulnerable individuals worldwide. Understanding the evolutionary relationships within the genus *Mycobacterium* is critical for improving species identification and, consequently, enhancing diagnosis, treatment, and epidemiological tracking. Pairwise comparisons of average nucleotide identity, genome–genome distance calculations, Mash values, multilocus sequence analyses, and average amino acid identities (AAIs) revealed that the AAI metric is the best to distinguish *Mycobacterium* from other genera of *Mycobacteriales*. Furthermore, genes encoding 16S and 23S rRNAs could also be used for the genus delineation: the previously established threshold of 94.5–95.0% of the *rrs* was confirmed, and the value for the *rrl* gene was estimated at 88.5–89.0%. The genus-delineating thresholds do not confirm the proposed splitting of the *Mycobacterium* into five genera, and the overall performance of conserved signatures used for splitting was not satisfactory. We estimated that *Mycobacterium* contains at least 402 distinct species, 246 of which were identified in clinical human specimens. The obtained tree and the corresponding list of species with proposed corrections to the names made from whole-genome sequences provide a reliable framework for the identification and taxonomic positioning of novel species within the genus.

## 1. Introduction

The genus *Mycobacterium*, part of the family *Mycobacteriaceae* and the order *Mycobacteriales*, includes numerous species, some of which are significant human and animal pathogens. The well-known pathogens are *Mycobacterium tuberculosis* and *Mycobacterium leprae*, the causative agents of tuberculosis and leprosy, respectively. Other species, collectively termed nontuberculous mycobacteria (NTMs), act as opportunistic pathogens in humans, particularly affecting immunocompromised individuals. A large list of acquired or inherited comorbidities includes bronchiectasis, cystic fibrosis, and chronic obstructive pulmonary disease [1]. Infections generally occur through everyday exposures, such as water–air aerosols, contact with open water sources, soil, birds, or animals. Unlike tuberculosis and leprosy, NTM infections are not transmissible between people, although evidence from the whole-genome sequencing has suggested nosocomial infections caused by rapidly growing mycobacteria of the *M. chelonaeabscessus* complex [2].

The detection of mycobacterial species in clinical samples is on the rise, facilitated by advances in molecular detection methods and the growing number of sequenced genomes [3,4,5,6]. Currently, more than 60 mycobacterial species are recognized as human pathogens [6].

The phylogenetics of the *Mycobacterium* genus is actively studied, and phylogenetic trees are constructed using various genome comparison methods that use nucleotide or protein sequence data [7,8,9]. These methods fall into two main categories: whole-genome sequence and multilocus approaches that involve selected conserved genes. The latter is similar to multilocus sequence analysis (MLSA) of isolates, although there is no consensus on the specific genes or proteins necessary for a reliable taxonomic reconstruction. The number of loci analyzed simultaneously in these studies ranges from a few [10] to hundreds [11,12].

Whole-genome approaches, including average nucleotide identity (ANI) [13], Mash distance [14], genome–genome distance calculator (GGDC) [15,16], and average amino acid identity (AAI) [17], are considered more reliable due to the large amount of information they utilize. However, the presence of different algorithm implementations and the scarcity of comprehensive method comparisons prevent a full reliance on any single approach. Consequently, phylogenetic trees from various studies often show discordant branches and are incomplete, representing only a fraction of known genomic sequences.

Compounding this issue, some deposited genomes have been incorrectly labeled due to ambiguities in species delineation when limited to single-locus sequence data. For example, the widely used 16S rRNA gene fragment alone is insufficient for precise species identification [18]. Other genes, such as *rpoB*, *gyrB*, and *hsp65*, which are widely used in clinical studies [19,20], offer increased reliability. However, the interpretation of these sequences is hampered by the lack of a comprehensive reference tree. In our previous studies of nontuberculous mycobacterial infections, we also identified a number of new species as unnamed species, the taxonomic position of which was not clear [21,22].

The recent proposal to divide the genus *Mycobacterium* into five separate genera based on vertically inherited traits [23] further complicates species identification [24,25] due to the parallel use of different nomenclatures. This dual naming convention is prevalent in both the literature and databases, adding to the confusion.

In this study, we analyzed mycobacterial genomes from the NCBI database and compared various evolutionary metrics for species delineation and reconstruction of the phylogenetic tree of *Mycobacterium*. We assessed the reliability of the phylogenetic reconstructions by evaluating the topologies of the resulting trees.

## 2. Results

### 2.1. Pairwise Comparison of Genome–Genome Distances

Whole-genome sequences of *Mycobacterium* (including also the ‘new’ genera *Mycolicibacterium*, *Mycolicibacillus*, *Mycolicibacter*, and *Mycobacteroides* [23]) were retrieved from NCBI. In total, 1390 genomes at various stages of completeness (from contigs to complete genomes) were used for further analysis. After initial sorting with the 95% ANI approach, 568 distinct mycobacterial species were identified. Excluding branches that contained genomes assembled from metagenomic studies alone, the list of mycobacterial species was shortened to 402. Part of the species were divided into subspecies (ANI = 95%–98%), resulting in a total of 497 distinct records (Table S1). In groups of closely related genomes, type strains with validly published status in the LPSN were preferred, and the sequence of a type strain was used.

The same analysis was performed for the *Hoyosella*, *Williamsia*, *Nocardia*, *Rhodococcus*, and other genomes of the *Mycobacteriales* order submitted to NCBI. In total, 315 genomes with assembly statuses ranging from contig to complete genome were used as an outgroup for the comparison of different genome–genome distances (Table S2).

**Figure 1:**
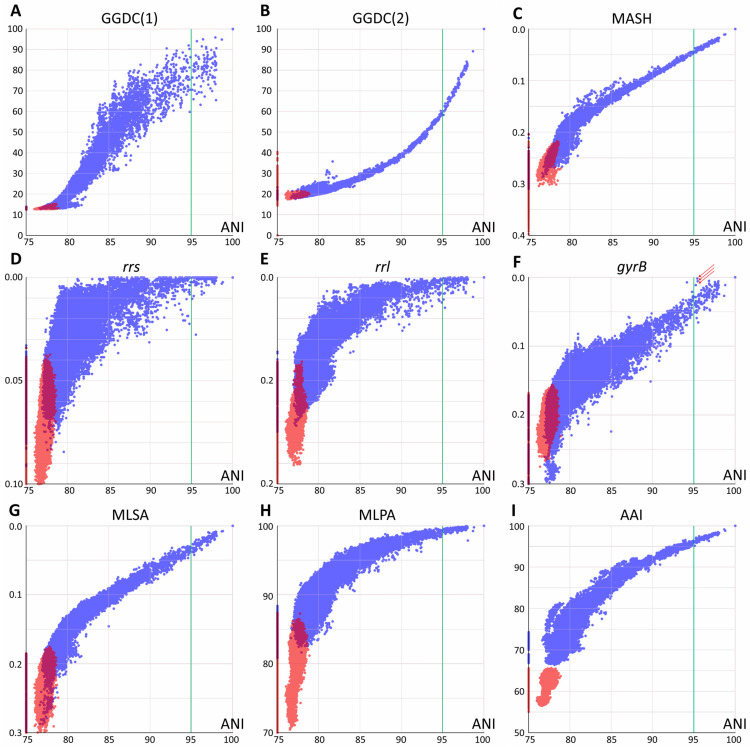
Pairwise comparison of evolutionary distances. Average nucleotide identity (ANI) was used as a reference. (**A**,**B**) Genome–genome distances calculator formulas 1 and 2, respectively; (**C**) Mash metrics; (**D**–**F**) Jukes–Cantor distance between *rrs* and *rrl* genes, coding for 16S and 23S ribosomal RNAs and the DNA-gyrase *gyrB* gene; arrows point to highly similar *gyrB* sequences for the *M. phocaicum* species violating the linear trend (see text) (**G**) distance obtained by multilocus sequence analysis; (**H**) distance obtained using 15 conservative protein sequences; (**I**) whole-genome amino acid identity distance AAI. Blue dots correspond to distances within the *Mycobacterium* genus (including *Mycobacteroides*, *Mycolicibacterium*, *Mycolicibacter*, and *Mycolicibacillus*), and red dots correspond to distances between *Mycobacterium* and species from other genera of *Mycobacteriales*. Purple areas refer to intersection of distributions. The species borderline ANI value of 95% represented by vertical green line.

Each pair of the joined set of *Mycobacteriales* genomes was compared using various methods based on their genomic sequences. Two groups of distances were analyzed: between species of the *Mycobacterium* genus and between *Mycobacterium* and other members of *Mycobacteriales*. The results of pairwise comparisons of distances are shown in Figure 1, with the ANI value used as a reference for all distances.

All whole-genome-based distances, except GGDC (formula 1), correlated well at ANI values of 90–100% or even lower with little error. This allows the identification of closely related genomes using any of these approaches. GGDC formula 1 has limitations in establishing phylogenetic relationships. The corresponding pairwise distribution of ANI-GGDC has a sigmoid form, with significant deviations toward greater GGDC distances or greater ANI similarity observed at high ANI values up to 100% (Figure 1). However, the second formula for GGDC correlates well with ANI values for closely related genomes. The reliable ranges of these distance values could be estimated between 85 and 100% for ANI and 0.00 and 0.15 for GGDC formula 2 (Figure 1B). For more diverse genomes, both calculations result in a high error. Mash metrics have a slight advantage over the ANI for comparison of less related genomes, as seen from the pairwise correlation rate change at 82% ANI (Figure 1C): below this value, a range of Mash values corresponds to the same ANI.

Single-gene distances yielded the expected results: both *rrs* and *rrl* genes that encode ribosomal rRNAs are limited in their applicability for species and subspecies delineation [18], since distinct species (ANI less than 95%) can have closely related gene sequences. However, in general, species with identical or highly similar *rrs* genes belong to the same clade, such as *M. septicum* and *M. peregrinum*, which are located in the *M. fortuitum* complex.

Analysis of other typical genes used for species identification and phylogenetic reconstruction revealed a different trend. For the DNA gyrase subunit gene *gyrB*, a linear trend between 80 and 100% ANI is observed, albeit with a higher error rate compared to whole-genome distances (Figure 1). Species delineation is possible using a single *gyrB* gene sequence. A minor violation of this rule observed as three dots with ANI about 95.2–95.4 and highly similar *gyrB* sequences (Figure 1F) was caused by comparison of subspecies within the highly diverse *M. phocaicum* species. Integration of many targets in MLSA analysis significantly decreases the error, and the 15 loci selected in this study result in the best performance compared to other nucleotide distances: the narrowest distribution and the best separation of intragenus and intergenera distances. The pairwise MLSA-ANI graph confirms that ANI metrics below 80–85% cannot be applied.

Pairwise analysis using amino acid sequences revealed distributions similar to those of nucleotide-based metrics; however, they had wider distributions compared to Mash, GGDC(2), and MLSA. The 15-protein metrics MPSA is more compressed toward 100% compared to the full-proteome AAI metrics; the latter provides better separation of genomes across the whole range studied.

### 2.2. Delineation of Mycobacterium Genus

Comparison of genome–genome distances showed one noticeable exception: AAI values allowed reliable discrimination between the *Mycobacterium* genomes (including the five ‘new’ genera) and other *Mycobacteriales* (Figure 1). The intersection of two cluster distributions converts to zero between 65.6 and 66.4%, which correlates well with previous estimates of the genus borderline at 65% [9].

**Figure 2:**
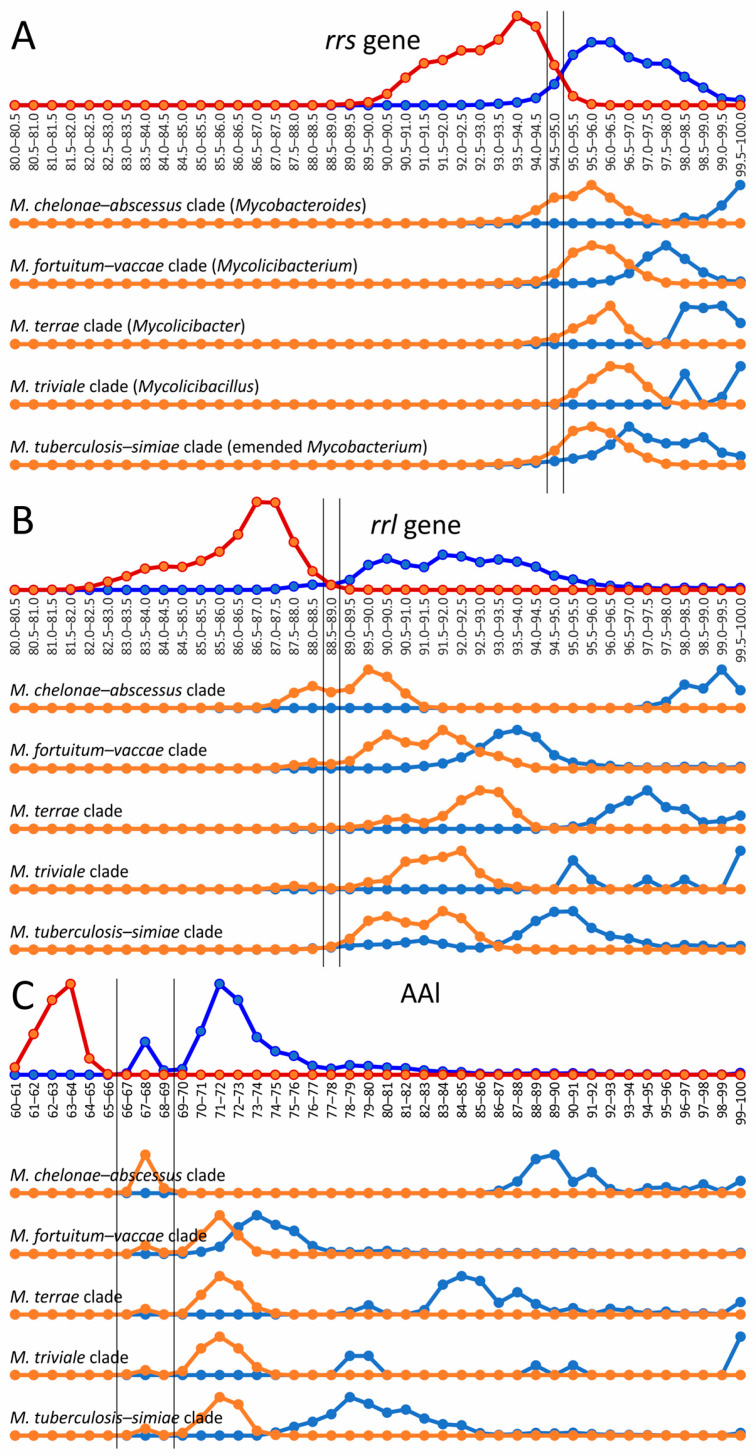
Genus delineation using the 16S, 23S rRNA gene distances and AAI metric. The evolutionary distances between species using the *rrs* (**A**) and *rrl* (**B**) genes and AAI metric (**C**) within the *Mycobacterium* genus (dark blue line) were compared to distances between *Mycobacterium* and species from other genera of *Mycobacteriales* (red line). The alternative splitting of the *Mycobacterium* into five genera was tested: genome–genome distances within the clade, corresponding to genus (light blue), compared to distances between genomes of this proposed genus and other genomes of *Mycobacterium* (orange line). Estimated borderline values for genus delineation are shown with vertical black lines.

The 16S rRNA gene *rrs* sequence is widely used for genus delineation, and the gene similarity threshold was estimated to be at 94.5–95.0% [26,27]. In line with these observations, two distributions of intragenus and intergenera sets of genome–genome distances separated well with a range of uncertainty between 94.5 and 95.5% *rrs* identity (Figure 2). Interestingly, the diversity of the 23S rRNA gene *rrl* was greater than that of *rrs*—the threshold that delineates the *Mycobacterium* genus was estimated to be between 88.5 and 89.0%. For *rrl*, the intersection of distributions was also not significant (Figure 2).

The same analysis was performed for the five mycobacterial clades—*M. chelonae*–*abscessus*, *M. fortuitum*–*vaccae*, *M. terrae*, *M. triviale*, and *M. tuberculosis*–*simiae*—which were assumed to represent separate genera [23]. The distances between the *rrs* and *rrl* genes were compared inside each cluster and between the analyzed and four other clusters (Figure 2).

In general, all intercluster distances were above 94.5% for *rrs* and 89% for *rrl* genes, which compromises the proposed genus splitting. However, a small part of the intercluster distance distribution for *M. chelonae*–*abscessus* versus all other *Mycobacterium* was above the genus delineation border (Figure 2). Although the discrimination of *M. chelonae*–*abscessus* using the *rrs* gene nearly perfectly falls within the intragenus range, 36% of intercluster distances for the *rrl* gene were above the genus-delineating threshold (Figure 2B). There was no significant difference for distances between *M. chelonae*–*abscessus* and other clusters analyzed individually; all clusters contained genomes whose distances to the members of *M. chelonae*–*abscessus* were as below and also above the threshold.

However, the distribution of AAI distances between genomes of the *Mycobacterium* cluster has a bimodal nature with peaks at 67–68% and at 71–72%. The former distribution corresponded to the distances between *M. chelonae*–*abscessus* and other *Mycobacterium* genomes. The exclusion of *M. chelonae*–*abscessus* genomes led to the extended gap at 66–69%, splitting two distance distributions inside the reduced *Mycobacterium* cluster and between this cluster and other genomes of *Mycobacteriales*.

### 2.3. Cluster Analysis of Genomes

**Figure 3:**
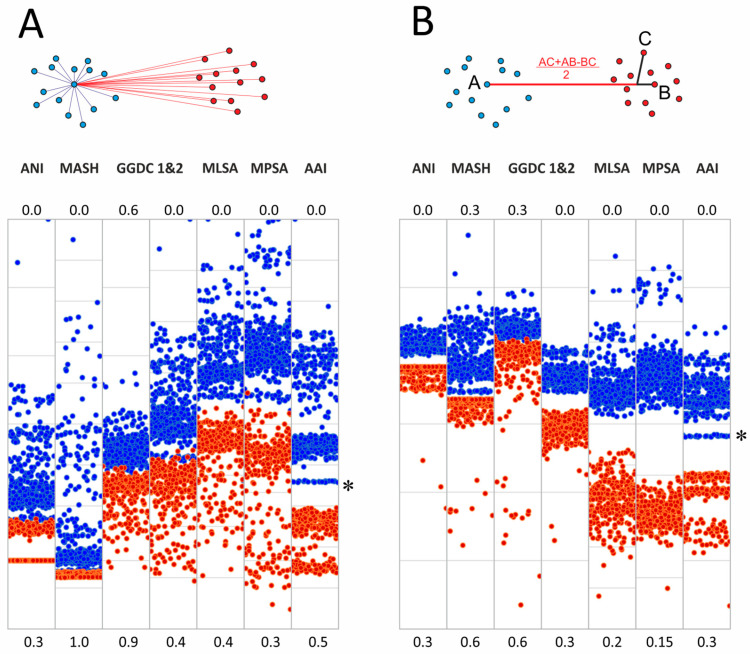
Cluster analysis of genomes of the order *Mycobacteriales* (**A**) Medoids approach and scatter plots of pairwise distances within the genus *Mycobacterium* (blue dots) and between the genomes of *Mycobacterium* and other *Mycobacteriales* (red dots). (**B**) Modified linear discriminant analysis of the same metrics. The position of the *M. chelonae*–*abscessus* cluster is marked with an asterisk.

Separation of genome groups could also be estimated based on cluster analysis methods, which are more advanced compared to the all-by-all approach of genome–genome distances described above. We tested two approaches for the determined genome–genome distances. The first is based on the determination of cluster medoids, and the second uses modified linear discriminant analysis (LDA) (Figure 3).

The medoid algorithm is based on finding the specific genome of one cluster whose average distance to other members of the cluster is minimal. The comparison of distances from the *Mycobacterium* medoids to other genomes in the cluster and genomes in the ‘other *Mycobacteriales*’ cluster is shown in Figure 3. In general, the discrimination of inter- and intracluster distances was improved for all metrics compared to the all-by-all approach. Thus, for MLSA and MPSA, the intersection of two distributions falls within 1%. Detailed distribution parameters are provided in Supplementary Table S3.

We also tested another approach for cluster delineation based on linear discriminant analysis, which is based on the projection of cluster dots onto a lower-dimensional space. The projection of a point C onto the AB line, which joins two dots that belong to different clusters, was determined by implying the phylogenetic tree topology (Figure 3B). The analysis included the selection of dots A and B, which provide the best separation of intra- and intercluster distances.

This approach further improved the separation of *Mycobacterium* from other *Mycobacteriales*. The complete discrimination with noticeable gaps between the two sets was observed for all distances except GGDC formula 1 (Figure 3). Delineating an unknown genome in this approach requires calculating two distances to these centroids of two clusters and calculating the projection using the formula given in Figure 3B.

Interestingly, we observed subcluster structure when AAI was used as the distance between genomes for both the medoid and LDA approaches (Figure 3). Separate bands reflect the presence of genetically related genomes within the analyzed clusters, which share a common ancestor. As expected, the distal band of *Mycobacterium*, and thus the closest to other *Mycobacteriales*, refers to the *M. chelonae*–*abscessus* complex (Figure 3, marked with an asterisk).

### 2.4. Phylogenetic Tree of Mycobacterium

Two phylogenetic trees for 402 different *Mycobacterium* species were built using the most robust 15-loci nucleotide distances (MLSA) and AAI. The average error estimated from the four-point rule for the AAI-implied tree was lower than that of the MLSA tree (12% vs. 17%). Additionally, a significantly greater number of branches had 100% support—192 vs. 67 (Figure S1), while the total number of internal branches was equal to 403 for both. This result strongly supports the best performance of the AAI metric for taxonomic reconstruction at the species and genus levels.

**Figure 4:**
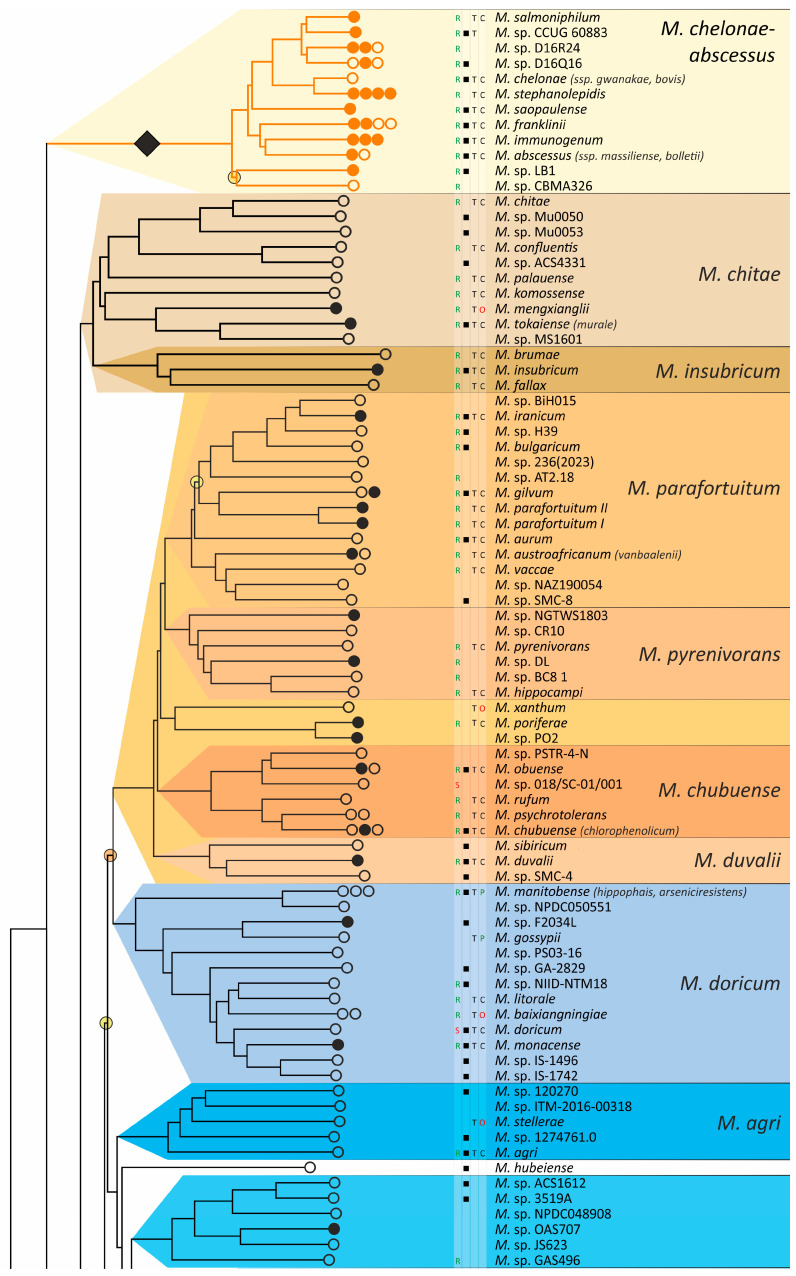

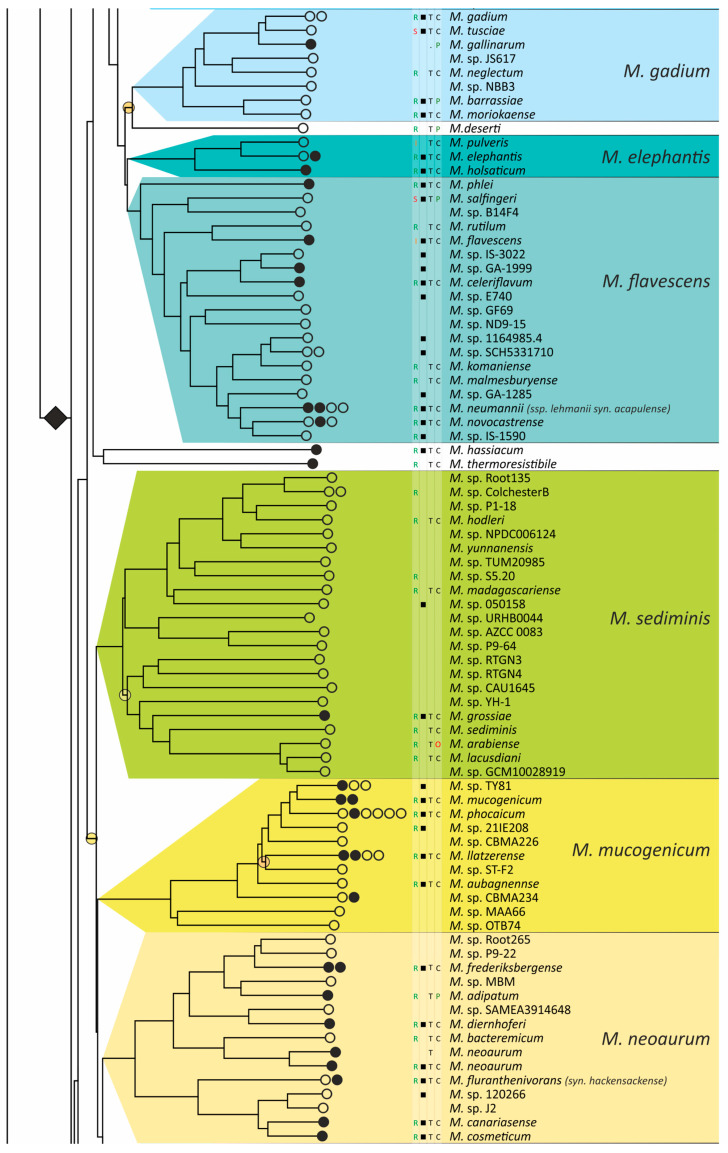

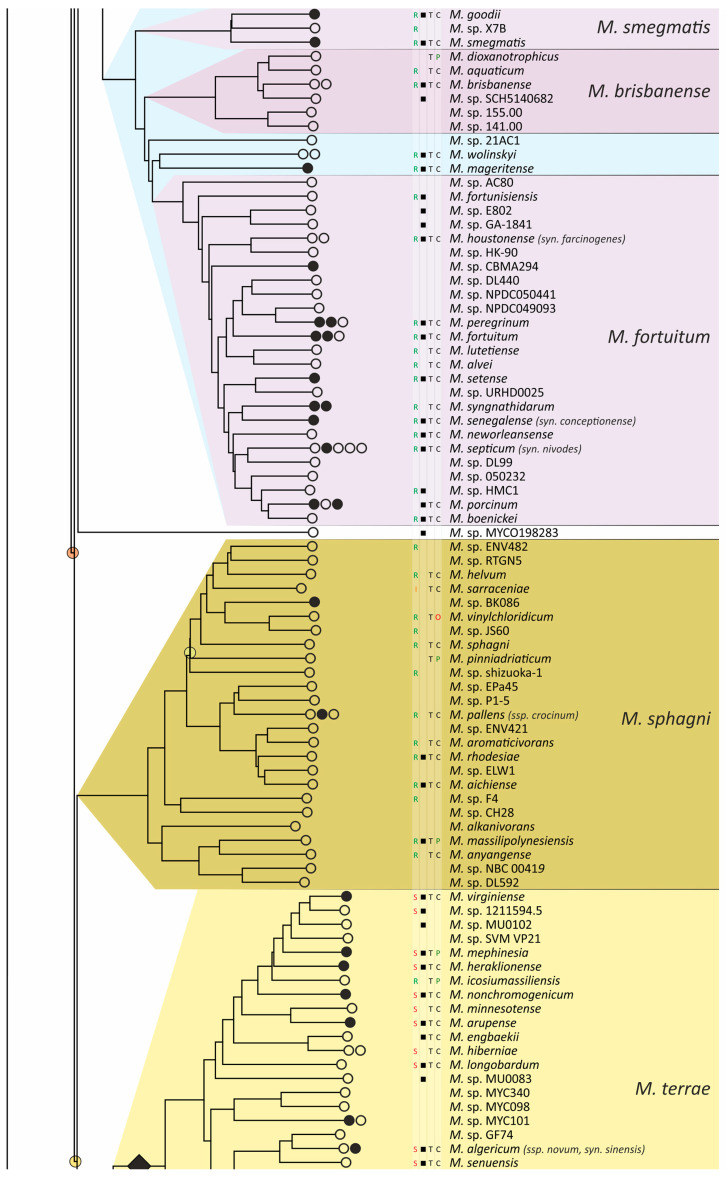

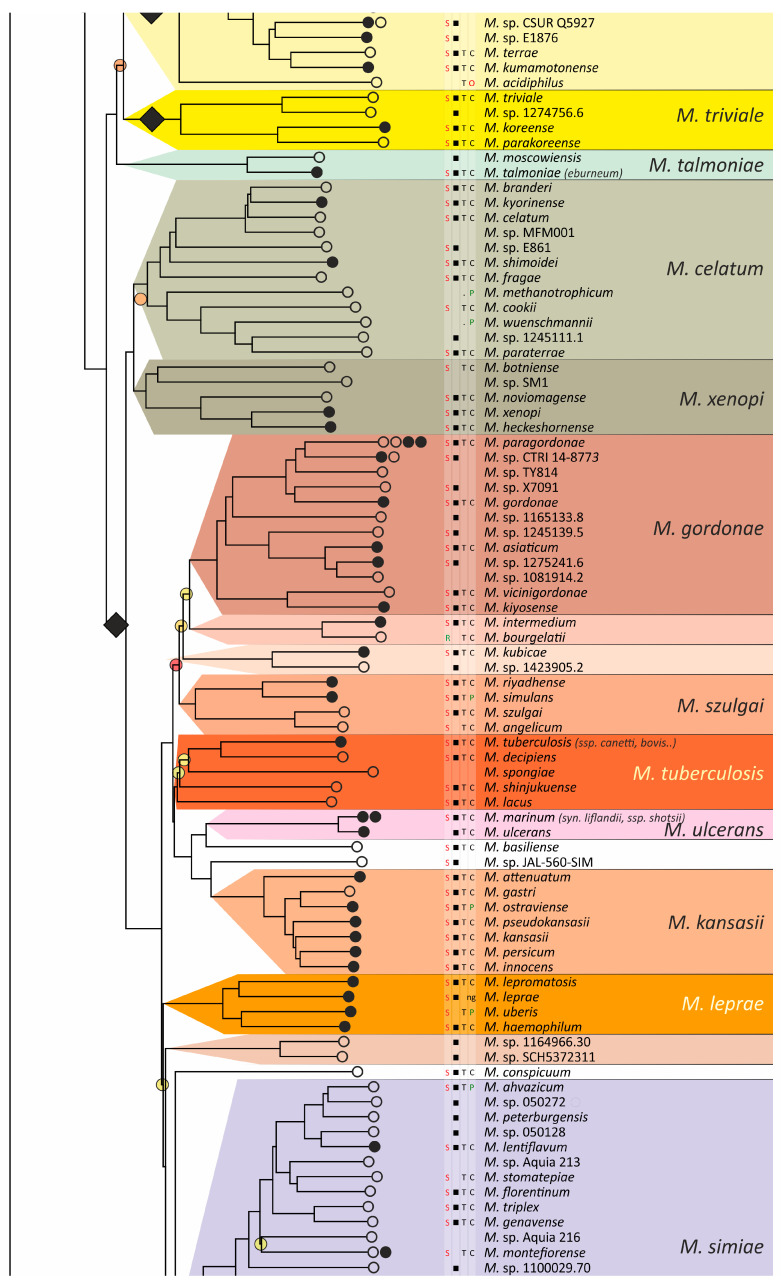

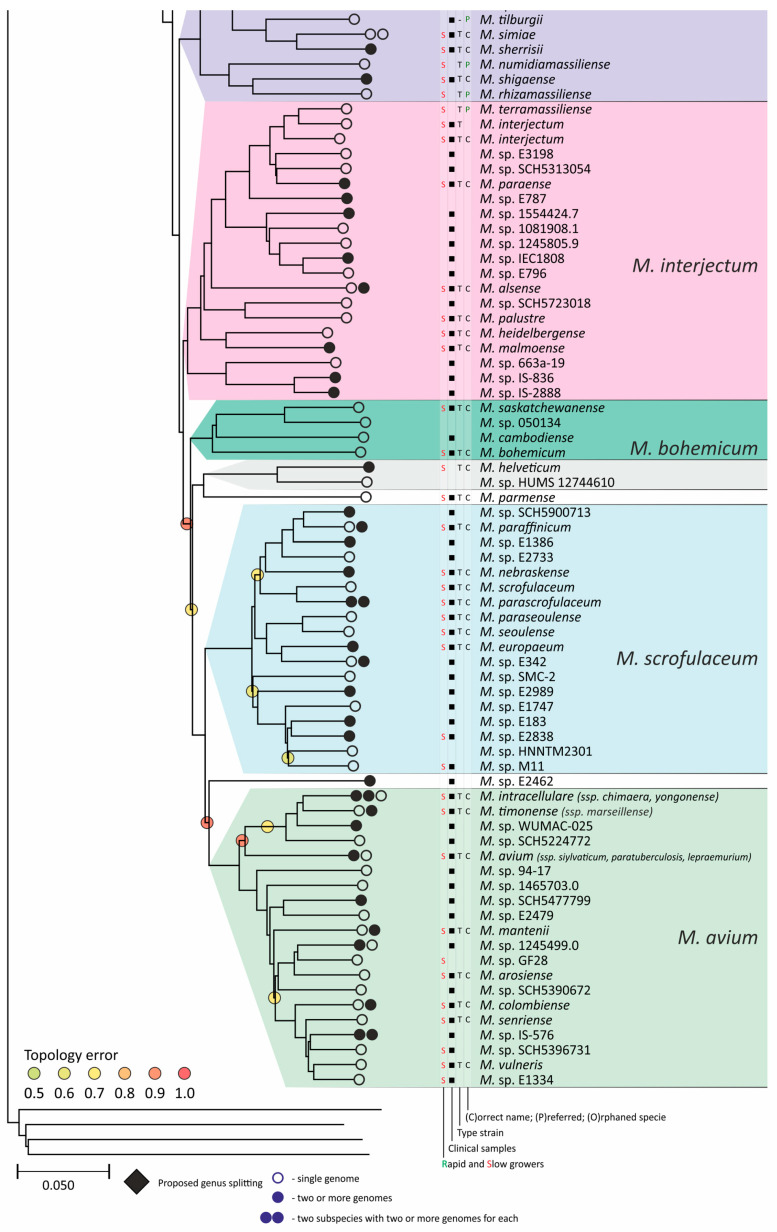
Phylogenetic tree of the genus *Mycobacterium*. The phylogenetic tree was obtained from AAI genome–genome distances; inner branches with topological error above 0.5 are marked with gradient-colored circles. Branches that corresponds to the proposed genera are pointed with black rhombs. Known and proposed clusters of species are coded with random colors. Each terminal branch corresponding to a species is designated with circles arranged horizontally, whose number reflects the number of subspecies. Empty circle point that only single genome was used for branch reconstruction, while filled circles indicate that more than one genome were available and were included in this study. The four columns left to the species names comprise data on the growth speed (rapid or slow), identification of the species in clinical human samples (black square), availability of the type strain in collections, and taxonomic sattus according to LPSN.

The obtained phylogenetic tree is characterized by a set of complexes or groups [28] rarely interspersed with orphan species (Figure 4). The *M. chelonae*–*abscessus* complex is the closest to the common ancestor of *Mycobacterium* and is rooted deeply and separately from other clades and species [9,11,29,30,31]. We propose that 12 distinct species are included in this complex, which is higher than it was established previously [32]. Only two species were represented by a single genome each: the fish pathogen *M. stephanolepidis* [33] and the strain isolated from the sputum of a patient with cystic fibrosis [34]. For other species, multiple genomes are available, and six of the species have been further divided into subspecies (Figure 4).

The branching of *Mycobacterium* into slow and rapid growers had one exception. The rapid-growing *M. sphagni* group was attached to the branch of slow-growing mycobacteria (Figure 4); however, an error rate of 66% indicated a local violation of the topology. The count of the {ac|bd} topology was 9,997,631, while those for the {ab|cd} and {ad|bc} topologies were 6,406,629 and 2,657,782, respectively. Here, branch {c} refers to the *M. sphagni* cluster; {d} refers to all slow-growing; {b} refers to rapidly growing *Mycobacterium* excluding the *M. chelonae*–*abscessus*, *M. chitae*, and *M. insubricum* groups; and {a} refers to the latter three clusters plus the root genomes of *Mycobacteriales*. Thus, the calculation of alternative quartet topologies indeed shows that the *M. sphagni* group belongs to the rapid-growers branch with a lower error of 47%, thus confirming the previous studies [11,29].

Rapidly growing mycobacteria are the predecessors of slow growers, with minor exceptions, probably due to later adaptation to ecological niches. Thus, while *M. icosiumassiliensis* and *M. bourgelatii* are rapid growers among the branch of slow-growing mycobacteria, *M. tusciae*, *M. salfingeri*, *M. doricum*, *M.* sp. 018/SC-01/001, and *M. insubricum* are slow growers located on the branch of rapid-growers of the tree [31].

Three groups, *M. terrae*, *M. triviale*, and *M. talmoniae*, are supposed to be placed in an intermediate position between slow- and rapid-growing [31], and indeed, they are rooted deeper than others on the slow-grower’s branch.

In addition to the *M. chelonae*–*abscessus* complex [35], other well-separated genomic groups [36] can be observed in the rapid-grower’s branch: *M. chitae* [37], *M. insubricum*, *M. parafortuitum* [38], *M. pyrenivorans* [11], *M. chubuense*, *M. duvalii* [11], *M. doricum* [37], *M. agri* [11], *M. gadium* [11], *M. elephantis* [11], *M. flavescens* [37], *M. sediminis* [11], *M. mucogenicum* [39], *M. neoaurum* [40], *M. smegmatis* [41], *M. brisbanense*, *M. fortuitum* [41], and *M. sphagni* [37]. Slow-growing *Mycobacterium* contain seventeen distinct groups: *M. terrae* [41], *M. triviale* [28], *M. talmoniae* [42], *M. celatum* [28], *M. xenopi* [7], *M. gordonae* [37], *M. kubicae* [25], *M. szulgai* [43], *M. tuberculosis*, *M. ulcerans* [37], *M. kansasii* [44], *M. leprae* [45], *M. simiae* [46], *M. interjectum* [37], *M. bohemicum*, *M. scrofulaceum* [28], and *M. avium* [47] (Figure 4).

Three novel groups of related species are proposed: *M. insubricum*, *M. brisbanense*, and *M. bohemicum*. Further, we used the *M. chubuense* naming instead of *M. chlorophenolicum* for the group of six species [11], based on the similarity of representative genomes and the priority of the *M. chubuense* naming [48]. All other cluster names are given in accordance with the earlier reports or the names of 16S rRNA clades used in Bergey’s manual [37]. The used *M. tuberculosis* group includes the *M. tuberculosis* complex itself and four other related species (Figure 4).

At least two larger supercomplexes could be proposed based on the existence of large common branches comprising several complexes. Thus, the *M. parafortuitum* supercomplex joins the *M. parafortuitum*, *M. pyrenivorans*, *M. poriferae*, *M. chubuense*, and *M. duvalii* complexes. Similarly, separate *M. fortuitum*, *M. smegmatis*, and *M. brisbanense* complexes, as well as three orphan species, are joined in a larger supercomplex, also known as the *M. fortuitum-smegmatis* group [7].

Many published genomes were not assigned any names and were not validly published [31]. Of the 402 species, only 181 had correct names, and 22 were marked as ‘preferred name’ in the LPSN [49]. Seven more species had an ‘orphaned’ status, and *M. leprae* was marked as ‘non-cultivated’. Other predicted unnamed or incorrectly named species (*n* = 191) were designated as *M.* sp. with the strain identification. The existence of 39 of these species was confirmed by the availability of several genomes that refer to subspecies or strains within species. In 151 of the total 402 species, several independent genomes were found. Approximately half of the genomes representing different species (*n* = 209) were obtained from the sequencing of type strains (Figure 4).

Sixteen species had alternative names caused by their independent discovery, and we propose that they constitute the same species according to distance measurements [11]. The data is summarized in Table 1. Most synonyms refer to separate subspecies within the same species, and the earliest name was used as the species name [50]. However, some exceptions to this rule were left unchanged. Thus, *M. fluoranthenivorans* [51] was described later than *M. hackensackense* [52], although the former is a correct name, while the latter is ‘preferred’ according to LPSN. An even more complex case is the correct naming of the species with the recommended name *M. algericum* [53]. Two other known genomes comprise the subspecies branch within this species, *M. sinensis* [54] and *M. novum* [55], which were discovered before *M. algericum.*

Four species with correct names according to the LPSN did not have sequenced genomes: *M. aquiterrae* [78], *M. arcueilense* [79], *M. montmartrense* [79], and *M. oryzae* [80]. The published sequences of 16S genes do not differ significantly from those of other known species. Thus, *M. arcueilense* and *M. montmartrense* are very close (0 and 7 mismatches) to *M. peregrinum* str. 852002-51209_SCH5440388, which we placed as a separate subspecies within the *M. peregrinum* represented by the DSM 43271 strain. The 16S rRNA gene of *M. oryzae* is similar to that of *Mycobacterium* str. djl-10 (SAMN05415090), which is a subspecies of *M. tokaiense*. The sequence of the *M. aquiterrae* 16S rRNA gene is similar to that of the SAMEA3906798 sample obtained in a metagenomic study [81].

In the whole list of 402 species, 37 are likely to have had incorrect annotations (Table S1). Thus, eight genomes annotated as *M. heraclionense* are distributed in separate *M. virginiense*, *M. nonchromogenicum*, and two orphan species within the *M. terrae* clade. They are located close to the correct *M. heraclionense* represented by the type strain JCM 30995. Furthermore, the greatest variation in taxonomic position was observed for the genomes annotated as *M. colombiense*, *M. asiaticum*, and *M. gordonae*, which included six, five, and four species, respectively.

We confirmed the division of *M. salmoniphilum* [82] into two separate species with an average ANI distance between them of 92% [32]. One species is represented by the type strains ATCC 13758, DSM 43276, CCUG 60884, and CCUG 62472, while the second is represented by CCUG 60883 and CCUG 60885. Both species are supported by the presence of other sequenced isolates [32].

We found two different species annotated as *M. malmoense* [83]. Four genomes, including that of the type strain DSM 44163, are grouped within the *M. interjectum* clade, which is in line with previous studies by Tortoli and Behra [11,29]. The other group of five strains, E826, E896, E614, E1298, and E3012 (PRJNA305922), which were isolated in Cambodia, are *M. parascrofulaceum* species within the separate *M. scrofulaceum* clade, represented by the genome of the type strain ATCC BAA-614.

Similarly, *M. neoaurum* [84] is split into two species represented by the type strains JCM 6365 (DSM 44074) and DSM 43536, which were previously revealed [85]. Both species were confirmed by independent isolation of the strains [86,87].

Additionally, genomes annotated as *M. sinensis* are found in two species, which we designated as *M.* sp. E1876 and *M.* sp. CSUR_Q5927, in addition to being correctly positioned as a subspecies of *M. algericum* based on the sequence of the type strain JDM601.

An unresolved situation was in the case of the presence of two alternative genomes for the same strain [88]. The first case was *M. interjectum* DSM 44064, which has two genomes, GCF_002102225.1 and GCF_025821415.1. These genomes are different, with ANI = 93.1%, Mash = 0.06, and AAI = 93.9%, which are below the species delineation thresholds. No other genomes are available for comparison. The second case was identified for the *M. parafortuitum* strains CCUG 20999 (GCF_002086815.1) and JCM 6367 (CCUG 20999, GCF_010725485.1). The genome–genome distances were close to the borderline values—ANI = 94.7%, AAI = 95.8%. Two other genomic sequences of different isolates are available that support both branches: GCF_900417285.1 and GCF_002946335.1. Individual gene sequences were also different for both cases, i.e., the *gyrB* gene of *M. interjectum* GCF_002102225.1 has 137 mismatches compared to *M. interjectum* GCF_025821415.1 per total length of 2030 bp. Therefore, these genomes are likely to correspond to separate species, and some sort of error had happened during strain deposition.

### 2.5. Phylogenetic Validation of Conserved Molecular Signatures

The lists of conserved molecular signatures, specific to the clades ‘*M. fortuitum*–*vaccae*’, slow-growing *Mycobacterium*, and ‘*M. tuberculosis*–*simiae*’, were validated using our more comprehensive set of genomes. According to the analysis by Gupta et al. [23], the clade ‘*M. fortuitum*–*vaccae*’ had four CSIs and ten CSPs, slow-growers had three CSIs and four CSPs, and ‘*M. tuberculosis*–*simiae*’ had three CSIs and three CSPs [23]. All protein homologs and corresponding indels were identified in the list of 402 mycobacterial genomes representing different species.

**Figure 5:**
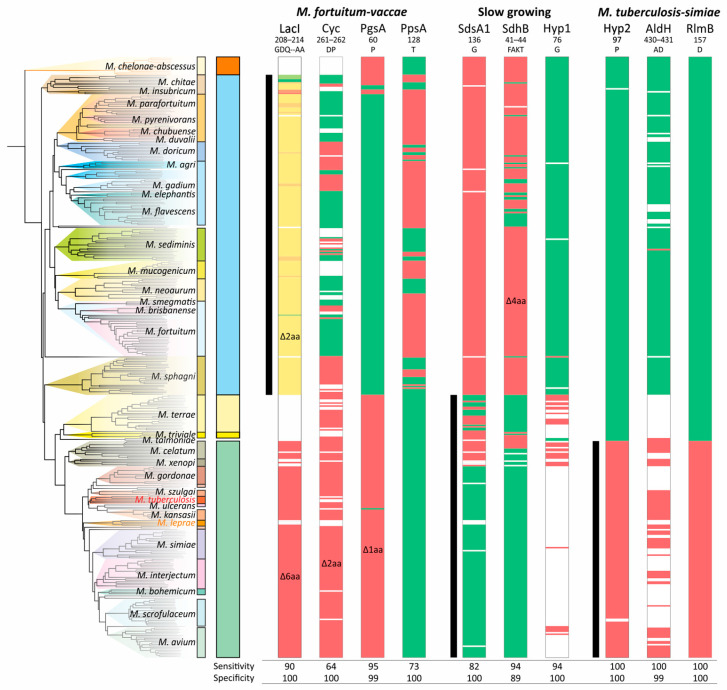
Distribution of the conserved signature indels (CSIs) along the phylogenetic tree of *Mycobacterium*. The presence and absence of the corresponding amino acid sequence are marked with green or red color, respectively. The white color indicates that the protein homolog is absent in the species. For the LacI-like protein, a gradual two-color scheme was used, designating the number of deleted amino acids from green (all present) to red (deletion of 6 amino acids). The ranges of species that belong to analyzed clades are shown with black vertical lines.

The analysis of CSIs showed that they do not necessarily belong to a single branch of the phylogenetic tree. While the deletion of two amino acids in LacI was more or less specific, the significant variability in the deletion of two amino acids (DP) in Cyc does not allow it to be used as a signature of the ‘*M. fortuitum*–*vaccae*’ clade—the sensitivity was only 64% (Figure 5). The specific distribution of CSI in PgsA was partially violated in the *M. chitae* group; deletion of the single amino acid in PpsA was not characteristic for the entire branch and has not occurred in the *M. sediminis*, *M. neoaurum*, and *M. sphagni* groups (Figure 5).

Furthermore, for the ‘*M. fortuitum*–*vaccae*’ clade, both LacI-like and Cyclase-like proteins were absent in a significant number of genomes, and thus the sensitivity/specificity values of their corresponding CSIs are lower than calculated. Similar results were found for the other two analyzed clades. The most robust CSIs were those of the hypothetical (Hyp2) and RlmB proteins for the ‘*M. tuberculosis*–*simiae*’ clade (Figure 5).

**Figure 6:**
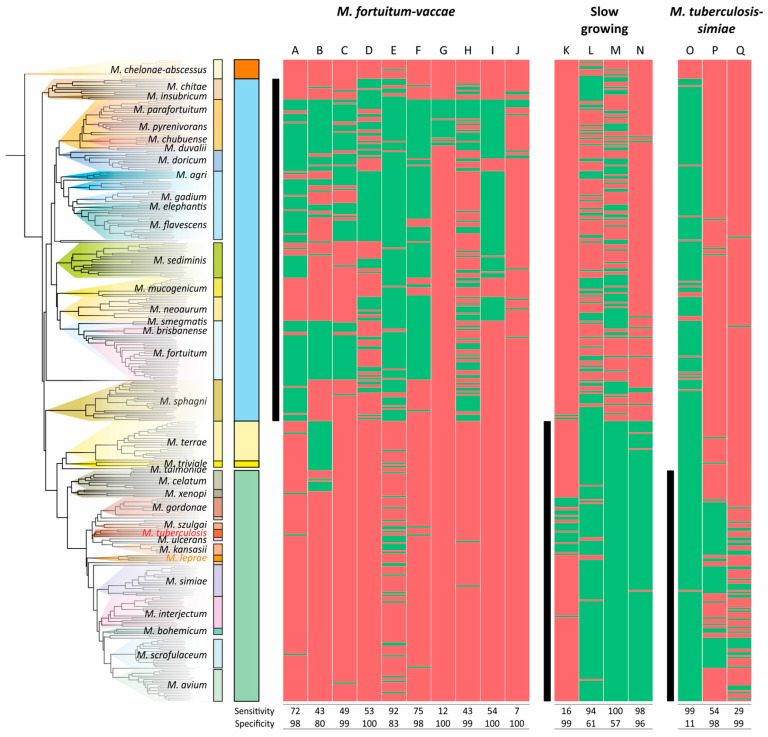
Distribution of conserved signature proteins (CSPs) along the phylogenetic tree of *Mycobacterium*. The list of proteins that separate the *M. fortuitum*–*vaccae* (*Mycolicibacterium*), slow-growers, and *M. tuberculosis*–*simiae* (emended *Mycobacterium*) clades was taken from the study by Gupta et al. [23]. The presence/absence of the protein homologue (>30% identity) is coded with green/red color, respectively. The ranges of species that belong to the analyzed clades are shown with black vertical lines.

The distributions of CSPs in mycobacterial species used as a marker for the division of the genera were even less sensitive and specific for the clades analyzed (Figure 6). The most robust CSPs were A, E, and N, while the average sensitivity and specificity for the 17 markers were 58% and 87%, respectively (Figure 6).

## 3. Discussion

Species delineation using partial or whole-genome sequencing is not a trivial task, even in the modern era of whole-genome sequencing and bioinformatics. Many different metrics are available for genome comparison based on nucleotide or protein sequences, which differ in their performance, calculation complexity, and error rates. The criteria for selecting the appropriate metrics for constructing phylogenetic trees are not always clear.

We compared various approaches for determining genome–genome distances using partial or complete genome sequences from a set of *Mycobacterium* genomes and compared these with other genomes of the order *Mycobacteriales*. All multilocus and whole-genome metrics correlated well for closely related genomes with ANI values greater than 90%. Therefore, species identification using any of the metrics and established delineation thresholds is reliable if any genome that belongs to the genus is available. That is, from the previously established ANI threshold of 95% [89], the corresponding Mash value from our study was 0.04–0.05.

The concept of borderline distance values delineating various taxa is appealing, but there are several considerations that complicate this process beyond the species level. First, error rates increase for more divergent genomes, as seen from the widening of pairwise comparison distributions. Furthermore, the trendline between any of the distances analyzed and the ANI showed the uncertainty of ANI values below 70–80%. The Mash method showed slightly better performance, but the multilocus nucleotide alignment distance (MLSA) and protein-based methods (MPSA and AAI) resulted in even greater improvement. These methods are characterized by a significantly lower intersection of pairwise distances within the genus and between genomes of different genera.

For AAI distances, complete discrimination of *Mycobacterium* from other *Mycobacteriales* was achieved at values below 66.4%, with the gap between two distributions in the range of 65.6–66.4%. A similar value of 65% was estimated in the study by Meehan et al. for the same genus [9]. However, in other studies, the AAI threshold values that delineate genera vary widely between 60% and 80% [26,90,91,92]. Evolution is not uniform across different taxa, and local trees can have significantly different shapes, complicating the establishment of clear borderlines between different taxa [93]. Whether a universal value could be proposed is a question of extensive ongoing studies [10].

The evolution of individual genes is not uniform and is influenced by horizontal transfer, and the analysis of many loci simultaneously minimizes the distance error. We confirmed that the *rrs* and *rrl* genes encoding the 16S and 23S rRNAs are impractical for species identification since many diverse species have nearly identical sequences. For species delineation, other conserved genes, such as *rpoB* or *gyrB*, are preferable. However, the rRNA genes provide nearly perfect discrimination at the genus level. Interestingly, the 16S rRNA gene is more conserved than the 23S rRNA gene, as was suggested earlier [94,95]. This was evident from the difference in the distributions of the intra- and intergenus distance and the established *Mycobacterium* genus borders: 94.5–95.0% for the *rrs* gene and 88.5–89.0% for *rrl*. While the observed *rrs* similarity threshold was previously estimated in a set of studies [26,27,96], and our value is in good agreement with it, the genus delineation threshold for *rrl* has not been established yet to the best of our knowledge.

The question of borderlines is tightly linked to the metrics and dimensionality of the intergenomic distance space. It was proposed that they are not Euclidean but belong to hypergeometric space with negative curvature [97,98]. In this metric, a triangle connecting three genomes has inward-curved borders. As the curvature decreases, the tree becomes closer to a three-ray star, which itself is a perfect representation of the smallest unit of the phylogenetic tree. The hypergeometric space better fits the four-point condition for phylogenetic trees and real data of intergenomic distances [98]. The most important consequence of this modeling approach is that distances cannot be compared directly, making the problem of adding new taxa to a phylogeny more complex than deriving borderline distances at different levels of classification [99].

It could also be proposed that the relationships of branches in the ideal phylogenetic tree are better defined by Manhattan (taxicab) distances or the Minkowski metric with *p* = 1. In that case, one must travel between genomes along the streets of a city where crossroads refer to the common ancestors of the branches. There are limitations to this space, so alternative roads cannot be selected to travel between genomes. However, considering the method of phylogenetic networks [100], the similarity becomes even more apparent.

Phylogenetic reconstruction is a subtask of the general mathematical problem of cluster analysis. There are numerous approaches for joining and splitting groups of objects based on the comparison of intra- and intercluster distances. A simple approach of pairwise comparisons of distances within a cluster and between clusters can provide reliable classification only if the dimensions of the clusters are smaller than the distance between them. Otherwise, intra- and intercluster ranges of distances will overlap, since the comparisons are performed in an all-by-all manner. Cluster analysis, a rapidly developing area of mathematics, offers many algorithms for solving this problem.

Limited by a matrix of pairwise distances instead of Euclidean coordinates, we applied two algorithms: an analysis of medoids and a modified linear discriminant analysis. While the medoids approach improved genus discrimination for all the metrics, the latter allowed the strict splitting of two distributions of distances inside the *Mycobacterium* and between *Mycobacterium* and other *Mycobacteriales*. The method maximizes the distance between two clusters by rotating the axis joining two dots that belong to separate clusters in a 2D space. LDA analysis also revealed fundamentally different distributions of AAI distances. We observed clusters splitting into subclusters, reflecting large groups of genomes joined by a common ancestor within the genus. For *Mycobacteriales*, sharp bands for different genera could be identified. For the genus *Mycobacterium*, the *M. chelonae*–*abscessus* clade is clearly separated and is located closer to other *Mycobacteriales*. We thus concluded that AAI metrics are additive and reliably reflect the true phylogenetic relationships within the order.

The tree topology validation was tested using the four-point rule on the distance matrix to estimate the internal branch support. This rule and the derived calculation of alternative quartet topologies are the cornerstones of many phylogenetic tree reconstruction methods [101,102,103]. In the approach by Chumakov and Iusmanov [103], errors were calculated at each terminal branch, allowing the estimation of the correctness of the genome or sequence position in the tree. We applied the same statistics to each internal branch, using all possible combinations of leaves (terminal branches) belonging to the four sets attached to the tested branch. Such statistics are also used as one method for tree comparison [104] and can serve for taxonomy evaluation, as in the quartet sampling method [105]. The comparison of errors for two alternative trees obtained from the MLSA and AAI distances confirmed the superiority of the latter distance: the average error was lower, and the number of branches with strong support (zero error) was higher.

The resulting phylogenetic tree of *Mycobacterium* is characterized by a significant number of ‘bushlike’ clades [93], where species diverged long ago and rapidly, resulting in short common branches with a high topology error rate. The existence of independent sequences corresponding to the same strain or closely related strains strongly confirmed the reliability of the phylogenetic analysis (Figure 4).

An important discussion raised by Gupta’s study [23] is whether *Mycobacterium* should be divided into five separate genera. Further genome studies opposed this proposal [9,10,31], and significant considerations related to clinical microbiology must be considered [24]. However, the novel genus names are used by NCBI, and recent studies [106] have used ‘new’ names along with the ‘old’ classification [25].

Our study confirmed the earlier finding by Turenne et al. that the taxonomic position of *M. talmoniae* (syn. *eburneum*) violates the proposed genus splitting [31]. This species is in the common branch of the *M. terrae* and *M. triviale* clades, closer to the latter. It cannot be directly ascribed to the *M. terrae* clade due to monophyly considerations, and following the idea of genus splitting, we would have to introduce another genus. There are three sequenced genomes of this species in the NCBI database: two for the type strain ATCC BAA-2683 and DSM 46873 and one for the isolate MO-5499 (SAMN05909063). This branch has an error of approximately 12%, which is close to the average error across the whole tree. Moreover, in our studies of clinical isolates of *Mycobacterium*, we identified a new species that belongs to the *M. talmoniae* branch with an ANI of 92% [42]. Thus, the presence of a separate *M. talmoniae* complex is undoubted and cannot be attributed to erroneous sequencing.

The proposed division of the genus *Mycobacterium* was based on the conception of conserved molecular signatures (synapomorphies), which are divided into clade-specific genes and indels [23]. The monophyletic presence of such signatures was supposed to reflect deep evolutionary valleys separating different genera. Our validation of the proposed traits on three large clades did not confirm its reliability for taxonomic positioning. We repeated the analysis proposed by Gupta using our significantly larger set of genomes and showed that the sensitivity/specificity of the proposed CSIs and CSPs were significantly lower than estimated previously. Most of the indels violated the proposed rules of genus splitting. It is worth mentioning that in the study by Gupta, the correlation of the presence of these markers was also not strict [23].

The proposed use of small deletions/insertions as significant evolutionary signatures is questionable from the general considerations. First, the phylogenetic trees of bacteria are reconstructed using the conservative housekeeping genes that are ubiquitous in the studied set [107]. In such genes, specific modifications of some conservative regions could indeed reflect phylogenetic relations. However, in the study by Gupta, the list of proteins included poorly characterized or hypothetical proteins [23]. We observed that not even all the corresponding genes could be identified in every genome studied. Furthermore, the use of the LacI-family transcriptional repressor gene cannot be justified since regulatory networks significantly vary between different species due to adaptation to different environmental conditions. For example, the *mmpS5-mmpL5* operon encoding the efflux complex in *M. tuberculosis* is regulated by the MarR-like MmpR5 repressor, while in *M. intracellulare* and *M. abscessus*, this operon is under the regulation of TetR-like repressors [108,109]. Thus, it was not unexpected that the distributions of conserved signature proteins were highly fragmented throughout the phylogenetic tree, and their sensitivities and specificities were not sufficient to serve as taxonomic traits.

Second, deletion of one or several amino acids is a questionable phylogenetic marker. For example, under the selective pressure of rifampicin, in addition to point mutations in the *rpoB* gene, deletions or insertions of several amino acids were also observed at and around codons 426, 431, 435, and 446 with noticeable total frequency [110]. Therefore, homoplastic deletion of several amino acids in the early stages of *Mycobacterium* divergence cannot be excluded. Indeed, while the CSIs were used for large taxon reconstruction [111], they were also prone to homoplasy [112], and thus cannot be the only criterion to judge the genus splitting.

Moreover, the single genome analysis as representative of the whole species could be influenced by intraspecies variability when the small number of events are analyzed. Using the CSIs and CSPs approach with a single event, we cannot be sure whether this trait is common to the whole species or is just the property of this particular strain. In the conventional alignment methods, the comparison is performed for long sequences or large bunches of short sequences, which corresponds to a range from thousands to millions of parameters (nucleotides or amino acids), thus minimizing the distance error.

The additional limitation of the CSI method is the absence of validated borderline values for delineation of genus. Contrary to that, the well-established borderline values for 16S rRNA gene similarity and genome-wide AAI metrics [26,27,91,96] did not confirm the proposed splitting. The values obtained in our study for the ‘old’ *Mycobacterium* were exactly the same as established in previous studies. The splitting violates these rules; thus, for the ‘*M. fortuitum*–*vaccae*’ clade (*Mycolicibacterium*), the average homology of the 16S rRNA gene with all other mycobacteria was 96.9%, with most of the range well above the borderline value of 94.5% for genera delineation [96].

One unresolved question remains with the taxonomic state of the *M. chelonae*–*abscessus* clade. Cluster analysis places it between *Mycobacterium* and other *Mycobacteriales*, but on the same branch as *Mycobacterium*. The length of the common branch of *M. chelonae–abscessus* was much greater than that of the common branches of *Corynebacterium*, *Nocardia*, and *Rhodococcus* (Figure S2). Previous studies confirmed the possibility of separating the clade into a genus [10]. However, the 16S and 23S rRNA genes were also similar to all other members of the genus *Mycobacterium* with values above thresholds, while some violation for the latter could be noticed (Figure 2).

On the other hand, the distribution of AAI distances inside the *Mycobacterium* allows us to suppose the existence of a separate genus corresponding to the *M. chelonae*–*abscessus* complex. The estimations of the threshold AAI value for bacterial genera delineation span from 60 to 74% [26,90,91,92,113]. In our study the gap in a range of 66–69% separating the distances within a genus and between genera appeared upon the exclusion of the *M. chelonae*–*abscessus* complex (Figure 2C). The distribution of AAI values between genomes of this complex and other mycobacterial genomes lies in this gap (Figure 2C). Therefore, we could suggest that the *M. chelonae*–*abscessus* complex represents a separate genus within the *Mycobacteriaceae* family together with the *Mycobacterium* genus. Further validation of obtained values using other bacterial orders would clarify the situation with the taxonomic rank of this complex.

In addition to many new unnamed species, the genome–genome distances and phylogenetic analysis of *Mycobacterium* showed that a significant number of genomes have ambiguous or incorrect names in the NCBI database, which also hampers the identification of mycobacterial species. On the other hand, intergenomic distances cannot be the only criterion for species delineation, and further splitting of species is probable. The criteria of physiological or clinical differences should be accounted for, as in the case of *M. marinum* and *M. ulcerans*, which cause different pathological processes [29].

In conclusion, our analysis indicated that the genus *Mycobacterium* contains at least 402 distinct species, and 246 species were identified in clinical human samples. While *M. avium* complex is the dominating cause of nontuberculous infections worldwide, the spectrum of species identified in clinical samples within the genus *Mycobacterium* and the order *Mycobacteriales* is expected to expand further. The species identification in the clinic should be based on a reliable molecular method such as the one proposed in this study.

## 4. Materials and Methods

### 4.1. Dataset

Mycobacterial genomes were retrieved from the NCBI Assembly database on 23 October 2024. The queries included the following terms: “*Mycobacterium*”, “*Mycobacteroides*”, “*Mycolicibacillus*”, “*Mycolicibacter*”, and “*Mycolicibacterium*”. Further in this study, the ‘old’ designation of *Mycobacterium* was used, which includes all ‘new’ genera. All genomic FASTA files with assembly statuses “Chromosome”, “Complete Genome”, “Scaffold”, and “Contig” were downloaded, except for *M. tuberculosis*, *M. abscessus*, *M. avium*, and *M. leprae* genomes, which were limited to not more than 50 of each. There were approximately 190,000 genomes of *M. tuberculosis*, 10,000 of *M. abscessus*, 4500 of *M. avium*, and 1000 of *M. leprae* as of the end of 2024.

Similarly, genomes of other *Mycobacteriales* were obtained for the genera *Hoyosella*, *Williamsia*, *Nocardia*, *Rhodococcus*, *Prescottella*, *Tsukamurella*, *Gordonia*, *Antrihabitans*, *Skermania*, *Tomitella*, *Aldersonia*, *Dietzia*, *Lawsonella*, *Corynebacterium*, and *Segniliparus*. In total, 315 genomes were used as the group ‘Other *Mycobacteriales*’ for comparisons of genome–genome distances.

The initial sorting of genomes into species and subspecies was performed using fastANI software (version 1.33) for comparison of whole-genome sequences [114], with a 95% threshold for species and 98% for subspecies [29]. The created database structure was highly similar to the resulting table presented in the supplements (Table S1). In a group of closely related genomes, type strains with validly published status in the List of Prokaryotic Names with Standing in Nomenclature (LPSN) [115] were preferred. Species-level clusters containing only sequences assembled from metagenomic studies were omitted from further analysis, thus leaving only 497 subspecies records comprising 402 separate species (Table S1). Data for the phenotypic division of species into rapid and slow growers were obtained from the NCBI Sample database, sample-related publications, and two recent studies [25,106].

### 4.2. Genome–Genome Distances

Pairwise comparisons of evolutionary distances were performed using the 497 genomes, corresponding to separate species/subspecies of *Mycobacterium*, and 315 genomes of various other species from other genera of the order *Mycobacteriales* (Table S2).

The average nucleotide identity (ANI) [13] was calculated using FastANI software (version 1.33), which is much less time-consuming and demanding in terms of computational resources compared to alternatives, with comparable results [114]. The software was installed in the Linux environment. Calculations were made by custom Python scripts using subprocess unit calls “fastANI -q {file1} -r {file2} -o {output}” and parsing of the output file.

Genome–genome distances (GGDC) [15,16] were calculated manually using the online server (http://ggdc.dsmz.de/ggdc.php, last accessed on 17 February 2024), since the standalone program is not available. Only seventy-five distances could be analyzed by a single query. Three alternative formulas for DNA–DNA hybridization (DDH) values were analyzed: formula 1 (also designated as formula *d*_0_): length of all high-scoring segment pairs (HSPs) divided by total genome length; formula 2 (or formula *d*_4_): sum of all identities found in HSPs divided by overall HSP length; and formula 3 (or formula *d*_6_): sum of all identities found in HSPs divided by total genome length [16]. No significant difference between the values with formulas 1 and 3 was obtained, so the third formula was omitted from the analysis.

Another whole-genome approach that allows rapid comparison of large datasets is Mash [14]. This method constructs MinHash sketches of genomes, allowing sequence-independent comparison of sets of hashes by calculating common hashes in two sequences. We used the recommended 22 nt k-mers with a relatively high sketch size of 100 K, which results in more precise values compared to those obtained with the default size of 1 K. Genome sketches were made using the command “mash-Linux64-v2.3/mash sketch -k 22 -s 100000 {file}”. Further calculations were made with the command “mash-Linux64-v2.3/mash dist {sketch1} {sketch2} > {output}” and parsing the output text file.

For multilocus sequence analysis (MLSA), 15 genes (*fusA*, *atpD*, *pheT*, *glnA*, *topA*, *secA*, *glpK*, *murC*, *pta*, *rrl*, *rrs*, *rpoB*, *recF*, *groL*, and *gyrB*) were randomly selected from the list of conservative genes [116]. The rationale for the use of 15 genes was based on the analysis of distance distributions obtained by varying the number of genes by a stepwise addition of genes to the calculation and estimation of the performance (Figure S3). The Pearson correlation coefficients for two distributions of distances within *Mycobacterium* and between *Mycobacterium* and other *Mycobacteriales* were compared at each step. Starting from 12 genes, the distributions became stable with correlation coefficients greater than 0.98. The gene sequences for the analyzed genomes were retrieved from genomic FASTA files by standalone BLAST software (version 2.14.0). Incomplete genes were omitted from the multilocus analysis. The Jukes–Cantor distances were measured as the average for 15 genes aligned in BLAST with default parameters.

Multiple peptide sequence analysis (MPSA) was based on fifteen protein sequences (RpsI, RpsH, RplJ, GrpE, RimP, Pnp, RsmH, PheT, SecA, AtpD, GyrB, MurC, RpoB, DnaN, Rnc) that were randomly selected from the list of 120 conserved bacterial proteins used for phylogenetic analysis [117]. Similarly to MLSA analysis, stepwise addition of protein sequences to calculation improved discrimination with further stabilization after n = 8 proteins accounting. The *tblastn* program from standalone BLAST was used for the retrieval of sequences from the genomes. Protein alignment was made using aligner and BLOSUM62 implemented in the Biopython package (version 1.81) [118].

The whole-genome average amino acid identity metrics (AAIs) [17] were calculated using the ezAAI implementation [119]. For each genome, protein extraction was made using the command “EzAAI extract -i {file} -o {protein}”. Alignment was called with the command “EzAAI calculate -i {protein1} -j {protein2} -o {output}” with further parsing of the output text file.

Pairwise distance comparisons and cluster analyses were performed using custom Python scripts; resulting tables were exported in csv format and visualized in Excel. As a result, the set of square matrices with dimensions (number of genomes) × (number of genomes) corresponding to different metrics was obtained.

### 4.3. Cluster Analysis

All calculations were performed using the standard NumPy library and custom Python scripts.

Further analysis of the discrimination of *Mycobacterium* from other *Mycobacteriales* was performed using two approaches from cluster analysis. The first approach used the identification of cluster medoid, which is defined as the point (genome) with the minimal average distance to all other points (genomes) in the same cluster [88,120]. Simple average distances to all other genomes in the cluster were calculated for each member of the cluster, and the genome with the minimal value was used as the medoid.

Then, the distributions of the distances from the medoid genome to the genomes of the same cluster and the other cluster were compared. We measured the average distances of the distributions, the range between the lowest intercluster distance and the highest intercluster distance, and the intersection of normalized distributions.

In the approach based on linear discriminant analysis, a projection of 2-dimensional genome space, represented by a pairwise square distance matrix, onto a single axis was made. Similarly to the medoid approach, instead of projecting onto an arbitrary line, the projection onto the line joining two points, A and B, from separate clusters was used.

Projections were calculated based on the phylogenetic tree model of clusters. For arbitrary genome C, the projection on the AB line reflects the distance to the last common ancestor and was calculated as AX = (AC + AB − BC)/2 (Figure 3). Two sets of projections of the same cluster and the remote cluster points were plotted, and the distributions were analyzed. The range of intersection of two distributions was used as a criterion for selection of dots A and B. All combinations of A from one cluster and B from the second were tested, and the best A and B providing the best discrimination were selected as representatives of the two clusters.

### 4.4. Phylogenetic Trees

Four genomes of the *Hoyosella*, *Antrihabitans*, *Tomitella*, and *Tsukamurella* genera were used as outgroups (GCF_026041215.1, GCF_012932915.1, GCF_029167405.1, GCF_023162105.1). The evolutionary history was inferred using the neighbor-joining method [121]. This analysis involved 402 *Mycobacterium* genomes representing the species. Evolutionary analyses were conducted in MEGA11 using the square distance matrixes [122]. The trees were minimally rearranged by swapping subtrees to better fit the phylogenetic tree proposed by Tortoli [29].

The tree topology estimation was analyzed using the four-point rule [101,102,103], similar to the recently described quartet sampling approach [105]. Every internal branch splits all terminal leaves (all genomes, including those used as root) into four independent sets, named quartets or quadruplexes. From the tree obtained, only one topology of such quadruplexes is implied—{ab|cd}, where the vertical line designates the internal branch that separates the two joined pairs. Here, the sum of distances ab + cd must be lower than both the ac + bd and ad + bc sums, and the latter two must be equal in the case of a perfect phylogenetic tree (Figure S3). Thus, the minimal sum of distance pairs determines the topology at the site, and it could differ from {ab|cd} in the case of real data. The distances for each of the three possible combinations of four leaves were compared following this rule [105], and all variants of selection of particular leaves from the four sets were tested. The error rate was calculated as the number of alternative topologies {ac|bd} or {ad|bc} that differ from the given {ab|cd} to the total number of combinations. Each internal branch of the tree was analyzed using a custom Python script. The branches with an error rate higher than 0.5 were marked on the resulting phylogenetic tree (Figure 4).

### 4.5. Identification of Conserved Signature Indels (CSIs) and Proteins (CSPs)

The lists of tested CSIs and CSPs were taken from the study by Gupta [23]. The *M. fortuitum*–*vaccae* clade contained 4 specific CSIs: a 5 aa insertion in LacI WP_036341761, a 2 aa insertion in Cyc WP_066808156, a 1 aa insertion in PgsA protein WP_036344961, and a 1 aa deletion in the PssA protein WP_066811333. Slow-growing *Mycobacterium* had 3 CSIs: a 1 aa insertion in SdsA1 protein WP_083113621, a 4 aa insertion in SdhB WP_083139296, and a 1 aa deletion in the hypothetical WP_009976218 (designated as Hyp1). The *M. tuberculosis*–*simiae* clade contained 3 CSIs: a 1 aa deletion in hypothetical WP_031701648 (Hyp2), a 2 aa deletion in WP_080699385 (AldH), and a 1 aa deletion in WP_083139967 (RlmB).

The lists of clade-specific CSPs included WP_048630777.1, WP_048632025.1, WP_048632497.1, WP_048634851.1, WP_048633467.1, WP_048633322.1, WP_048631132.1, WP_048634509.1, WP_048630657.1, and WP_048632441.1 for the *M. fortuitum*–*vaccae* clade (designated as A, B, C, …, J further); YP_177721.1, YP_178025.1, WP_011725130.1, and WP_003874405.1 (designated from K to M) for slow-growers; and NP_218369.1, YP_004837050.1, and NP_217322.1 (O, P, and Q, respectively) for the *M. tuberculosis*–*simiae* (emended *Mycobacterium*) clade.

The search for protein homologs was performed using the *tblast* program from the standalone BLAST package (version 2.14.0). The similarity greater than 30% was considered significant [123] to draw conclusions about the presence of the corresponding protein homologue in the genome. CSIs were further analyzed by performing the multiple alignment of the protein sequences with MEGA11 software.

The sensitivities and specificities of the presence of CSIs and CSPs in a particular clade were estimated from all genomes in which the corresponding protein was identified. All indel and protein lists are identical and are shown in figures exactly as used in Tables 4 and 5 in the study by Gupta et al. [23].

## Figures and Tables

**Table 1:** Closely related strains with alternative species names.

No	Species	Subspecies	Strain	Species	Taxonomic Status	Type Strain	Strain	Reference
1	+	+	+	*M. tokaiense*	correct name	T	ATCC 27282	Tsukamura, 1981 [48]
			+	*M. murale*	correct name	T	DSM 44340	Vuorio, 1999 [56]
2	+	+	+	*M. neumannii*	correct name	T	CECT 8766	Nouioui, 2017 [57]
		+	+	*M. lehmannii*	correct name	T	CECT 8763	Nouioui, 2017 [57]
			+	*M. acapulense*	preferred name		CSUR P1424	Gupta, 2018 [23]
3	+	+	+	*M. manitobense*	preferred name	T	DSM 44615	Turenne, 2003 [58]
		+	+	*M. hippophais*	preferred name	T	CPCC 205372	Deng, 2023 [59]
		+	+	*M. arseniciresistens*	orphaned species	T	KC 300	Zhu, 2024 [60]
4	+	+	+	*M. chubuense*	correct name	T	DSM 44219	Tsukamura, 1981 [48]
		+	+	*M. chlorophenolicum*	correct name	T	JCM 7439	Häggblom, 1994 [61]
5	+	+	+	*M. obuense*	correct name	T	DSM 44075	Tsukamura, 1981 [48]
		+	+	*M. kyogaense*	correct name	T	DSM 107316	Nouioui, 2018 [62]
6	+	+	+	*M. houstonense*	correct name	T	ATCC 49403	Schinsky, 2004 [63]
		+	+	*M. farcinogenes*	correct name	T	DSM 43637	Chamoiseau, 1973 [64]
7	+	+	+	*M. senegalense*	correct name	T	ATCC 35796	Chamoiseau, 1979 [65]
			+	*M. conceptionense*	correct name	T	CCUG 50187	Adékambi, 2006 [66]
8	+	+	+	*M. septicum*	correct name	T	DSM 44393	Schinsky, 2000 [67]
		+	+	*M. nivoides*	orphaned species	T	DL90	Dahl, 2019 [68]
9	+	+	+	*M. fluoranthenivorans*	correct name	T	JCM 14741	Hormisch, 2004 [51]
		+	+	*M. hackensackense*	preferred name	T	DSM 44833	Hong, 2003 [52]
10	+	+	+	*M. pallens*	correct name	T	JCM 16370	Hennessee, 2009 [69]
		+	+	*M. crocinum*	correct name	T	JCM 16369	Hennessee, 2009 [69]
11	+	+	+	*M. hiberniae*	correct name	T	ATCC 49874	Kazda, 1993 [70]
		+	+	*M. engbaekii*	correct name	T	ATCC 27353	Tortoli, 2013 [71]
12	+	+	+	*M. algericum*	correct name	T	DSM 45454	Sahraoui, 2011 [53]
		+	+	*M. sinensis*	preferred name	T	JDM601	Mun, 2008 [54]
			+	*M. novum*		T	JCM 6391	Tsukamura, 1967 [55]
13	+	+	+	*M. eburneum*	correct name	T	DSM 44358	Nouioui, 2017 [72]
			+	*M. talmoniae*	correct name	T	ATCC BAA-2683	Davidson, 2017 [73]
14	+	+	+	*M. marinum*	correct name	T	CCUG 20998	Aronson, 1926 [74]
		+	+	*M. shottsii*		T	JCM 12657	Rhodes, 2003 [75]
15	+	+	+	*M. timonense*	correct name	T	JCM 30726	Ben Salah, 2009 [76]
		+	+	*M. marseillense*	correct name	T	DSM 45437	Ben Salah, 2009 [76]
16	+	+	+	*M. ulcerans*	correct name	T	ATCC 19423	MacCallum, 1950 [77]
			+	*M. pseudoshottsii*	correct name	T	DSM 45108	Rhodes, 2005 [50]

## Data Availability

The datasets generated and/or analyzed during the current study are available in the GitHub repository, https://github.com/DanZimenkov/Mycobacterium-2024 (accessed on 14 August 2025).

## Supplementary Materials

The following supporting information can be downloaded at: https://www.mdpi.com/article/10.3390/ijms262110471/s1.
