# Supplementary material for: Comparative Analysis of Evolutionary Distances Using the Genus Mycobacterium
Source: Int J Mol Sci. 2025 Oct 28;26(21):10471. doi: 10.3390/ijms262110471 (PMC12607691; doi:10.3390/ijms262110471)
Supplement: Supplementary file 1 [file ijms-26-10471-s001.zip › ijms-3847462-supplementary.pdf]

Supplementary materials

Figure S1. Error rates of the internal branches for MLSA and AAI trees.

(**A**) – The four points rule for the topology {AB|CD} declares that the sum of distances AB + CD must be lower than both AC + BD and AD + BC, and the latter two must be equal in the case of a perfect phylogenetic tree. (**B**) – The existense of the internal branch X was tested for all combinations of terminal branches (colored). The error rate was estimated as a number of alternative {AC|BD} or {AD|BC} to the total number of combinations. (**C**) – internal branch errors of trees constructed using AAI and MLSA distances, ordered in descending order.

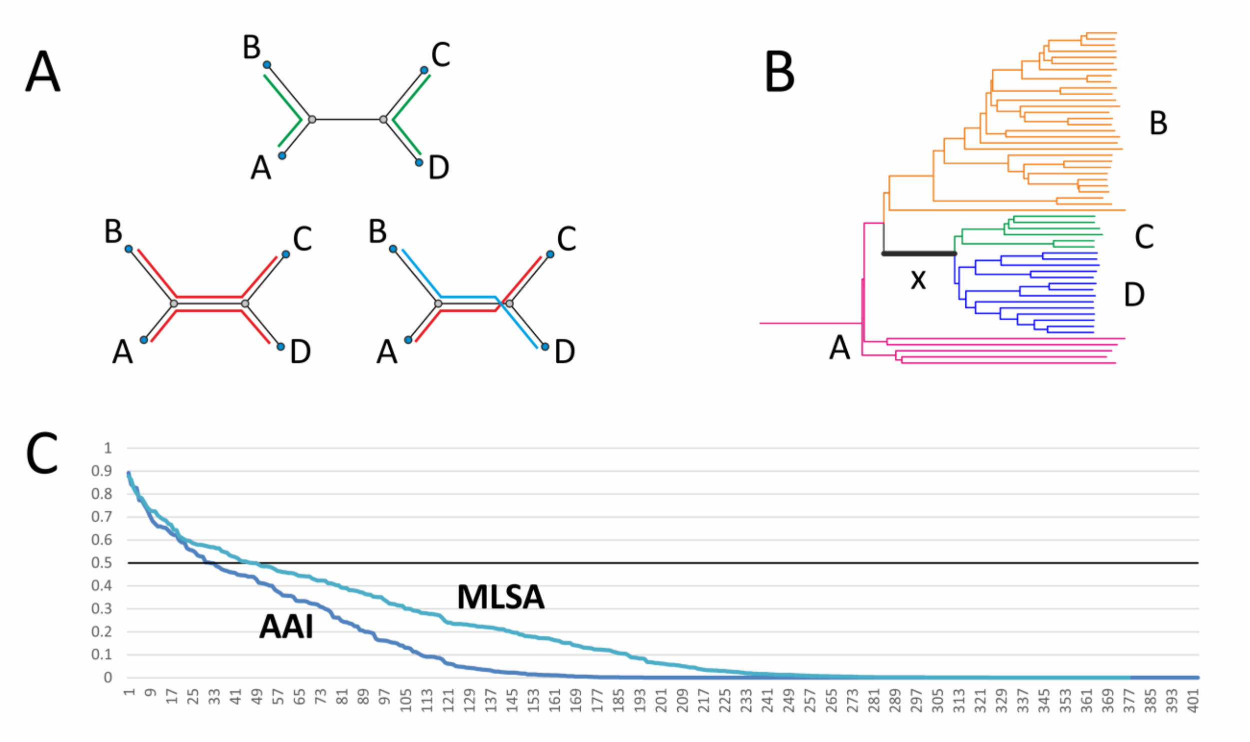

Figure S2. Phylogenetic tree of order *Mycobacteriales*.

The tree was obtained by NJ-method from AAI genome-genome distances for 471 genome of *Mycobacteria* and 315 genomes of other *Mycobacteriales*. The tree is rooted on 4 genomes of *Microbacterium*. Main clades of *Mycobacteria*, which are proposed to represent separate genera identified in publication by Gupta, 2018 are marked with different colors.

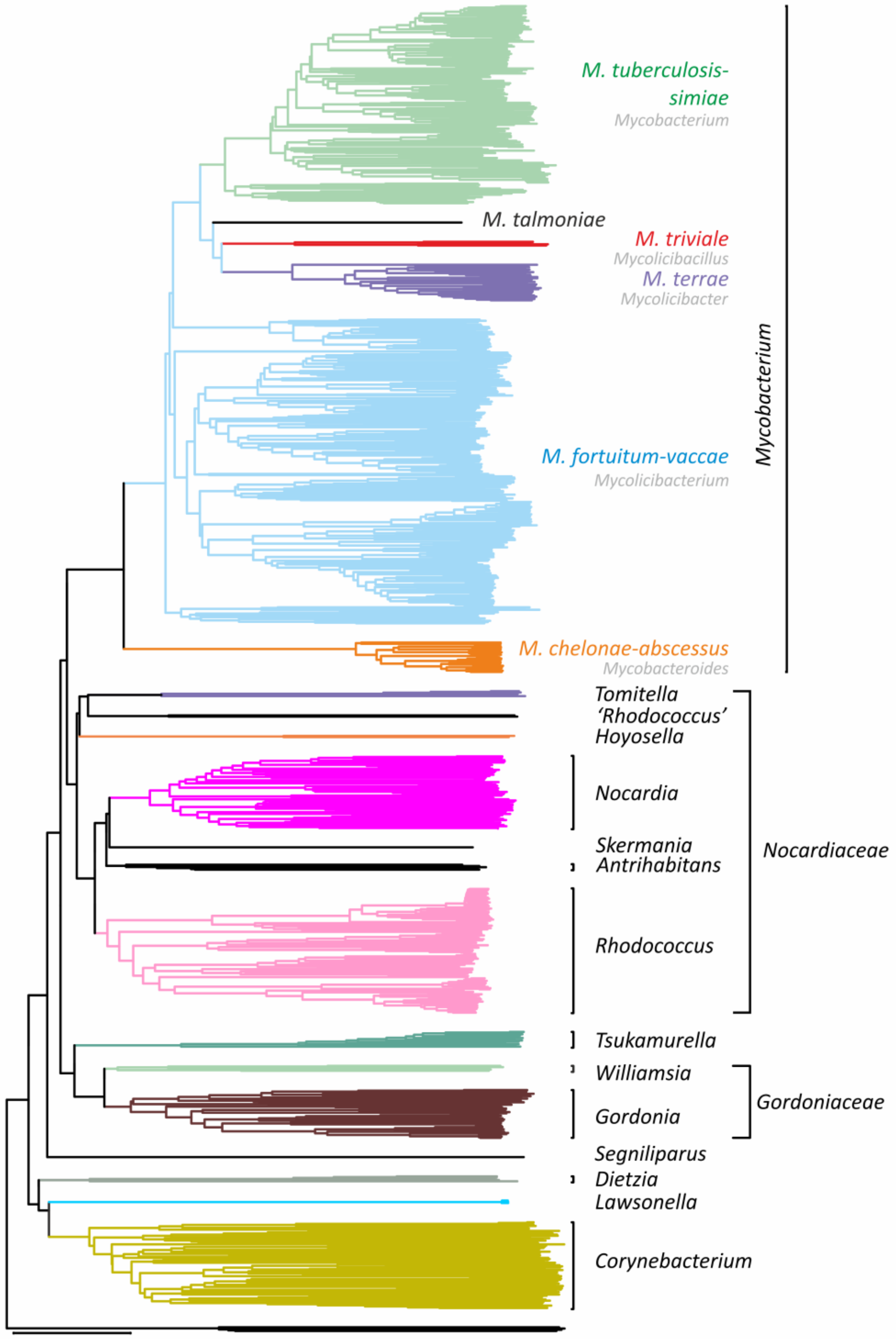

Figure S3. The dependence of MLSA analysis on the number of genes enrolled.

The evolutionary distances between species within the *Mycobacterium* genus (blue line) were compared to distances between *Mycobacterium* and species from other genera of *Mycobacteriales* (red line) in a stepwise manner from single gene (*rrs*) to a total of 15 genes. The distributions were estimated by correlation analysis of distances (Pearson coefficient) at each step compared to previous (color-coded accordingly with two distributions).

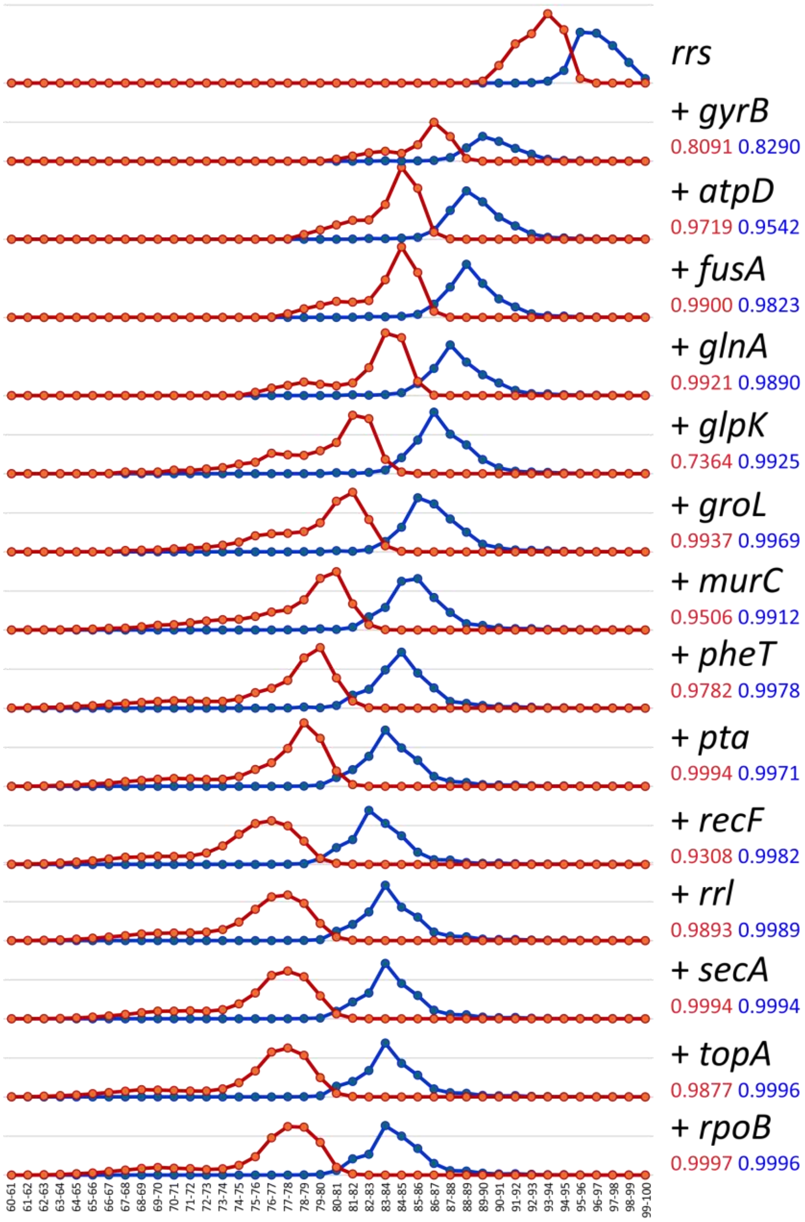

Table S1. List of analyzed *Mycobacterium* genomes.

The list of species is given in the same order as at the phylogenetic tree (maintext Figure 4). Separate species are separated by horisontal lines. The list of genomes within a specie is given in the hierarchical order. Species names in NCBI annotation column are color-coded if the same name for separate species exists: incorrect annotation is given in red, the correct one is green. Alternative namings for the same species are given in blue. Colors for species complexes are used according the Figure 4 in the maintext.

Species group/complex	Taxonomic level: Specie	Subspecie	Strain	NCBI annotation	Sample	Genome	Specie reannotation
M. chelonae - abscessus	+	+	+	*M. salmoniphilum*	SAMN05186798	GCF_002013685.1	
			+	*M. salmoniphilum*	SAMN07807803	GCF_004924335.1	
		+	+	*M. salmoniphilum*	SAMN07807799	GCF_004367915.1	
			+	*M. salmoniphilum*	SAMN07807801	GCF_004366875.1	
		+	+	*M. salmoniphilum*	SAMN05186785	GCF_002013645.1	
	+	+	+	*M. salmoniphilum*	SAMN07807798	GCF_004366675.1	*M.* sp. CCUG_60883
			+	*M. salmoniphilum*	SAMN07807800	GCF_004367255.1	*M.* sp. CCUG_60883
			+	*M. sp. D16R18*	SAMN05186784	GCF_002013775.1	*M.* sp. CCUG_60883
		+	+	*M. salmoniphilum*	SAMN07807810	GCF_004367265.1	*M.* sp. CCUG_60883
			+	*M. salmoniphilum*	SAMN07807808	GCF_004366645.1	*M.* sp. CCUG_60883
			+	*M. salmoniphilum*	SAMN07807809	GCF_004366855.1	*M.* sp. CCUG_60883
		+	+	*M.* sp. D16R12	SAMN05186783	GCF_002013465.1	*M.* sp. CCUG_60883
	+	+	+	*M.* sp. D16R24	SAMN05186774	GCF_002013385.1	*M.* sp. D16R24
			+	*M.* sp. D16Q14	SAMN05186777	GCF_002013415.1	*M.* sp. D16R24
			+	*M.* sp. D17A2	SAMN05186782	GCF_002013425.1	*M.* sp. D16R24
			+	*M.* sp. D16Q20	SAMN05186780	GCF_002013785.1	*M.* sp. D16R24
	+	+	+	*M.* sp. D16Q16	SAMN05186778	GCF_002013745.1	*M.* sp. D16Q16
			+	*M.* sp. D16Q13	SAMN05186776	GCF_002013375.1	*M.* sp. D16Q16
			+	*M.* sp. 96-892	SAMN05186794	GCF_002013545.1	*M.* sp. D16Q16
	+	+	+	*M. chelonae subsp. chelonae*	SAMN02630629	GCF_001632805.1	
			+	*M. chelonae*	SAMEA4040484	GCF_900637085.1	
		+	+	*M. chelonae subsp. gwanakae*	SAMN09770287	GCF_003390495.1	
			+	*M.* sp. MHSD3	SAMN06611218	GCF_002843405.1	
			+	*M.* sp. QIA-37	SAMN04613683	GCF_001611855.1	
		+	+	*M.* sp. H001	SAMN04091131	GCF_001440005.1	
			+	*M.* sp. H002	SAMN04091132	GCF_001440085.1	
			+	*M.* sp. H072	SAMN04091134	GCF_001440105.1	
			+	*M.* sp. H054	SAMN04091133	GCF_001440125.1	
		+	+	*M.* sp. HXXIII	SAMN04091155	GCF_001440305.1	
			+	*M.* sp. H003	SAMN04091147	GCF_001440135.1	
			+	*M.* sp. H063	SAMN04091148	GCF_001440155.1	
			+	*M.* sp. H070	SAMN04091149	GCF_001440185.1	
			+	*M.* sp. H079	SAMN04091150	GCF_001440205.1	

M. chelonae - abscessus			+	*M.* sp. H092	SAMN04091151	GCF_001440225.1	
			+	*M.* sp. H101	SAMN04091152	GCF_001440245.1	
			+	*M.* sp. HXVII	SAMN04091154	GCF_001440275.1	
	+	+	+	*M. stephanolepidis*	SAMD00081530	GCF_002356335.1	
	+	+	+	*M. saopaulense*	SAMN06064258	GCF_002086715.1	
			+	*M. saopaulense*	SAMN03225541	GCF_001456355.1	
			+	*M. saopaulense*	SAMN05897984	GCF_001853625.1	
			+	*M. saopaulense*	SAMN05897989	GCF_001853765.1	
	+	+	+	*M. franklinii*	SAMN06064239	GCF_002086225.1	
			+	*M. franklinii*	SAMN10588797	GCF_004355025.1	
			+	*M. franklinii*	SAMN05186797	GCF_002013895.1	
			+	*M. franklinii*	SAMN07807806	GCF_004366145.1	
			+	*M. franklinii*	SAMN07807802	GCF_004366895.1	
			+	*M. franklinii*	SAMN07807805	GCF_004367185.1	
			+	*M. franklinii*	SAMN07807804	GCF_004367235.1	
			+	*M. franklinii*	SAMN07807807	GCF_004367295.1	
		+	+	*M. franklinii*	SAMN05186779	GCF_002013715.1	
			+	*M. franklinii*	SAMN05186775	GCF_002013295.1	
		+	+	*M. franklinii*	SAMN03354702	GCF_011064445.1	
		+	+	*M. franklinii*	SAMN05897992	GCF_001853795.1	
	+	+	+	*M. immunogenum*	SAMN03733495	GCF_001605725.1	
			+	*M. immunogenum*	SAMN03295724	GCF_000878425.1	
			+	*M. immunogenum*	SAMN04025494	GCF_001296245.1	
			+	*M. immunogenum*	SAMN04025495	GCF_001296255.1	
			+	*M. immunogenum*	SAMN04025493	GCF_001296265.1	
			+	*M. immunogenum*	SAMN04025496	GCF_001296325.1	
			+	*M. immunogenum*	SAMN04025497	GCF_001296345.1	
			+	*M. immunogenum*	SAMN04025499	GCF_001296385.1	
			+	*M. immunogenum*	SAMN04025500	GCF_001296405.1	
			+	*M. immunogenum*	SAMN04025498	GCF_001296415.1	
			+	*M. immunogenum*	SAMN04025502	GCF_001296425.1	
			+	*M. immunogenum*	SAMN05244438	GCF_001677135.1	
			+	*M. immunogenum*	SAMN05186796	GCF_002013555.1	
			+	*M. immunogenum*	SAMN04216931	GCF_002101665.1	
			+	*M. immunogenum*	SAMN15568933	GCF_025822945.1	
		+	+	*M. immunogenum*	SAMN04395166	GCF_001655155.1	
	+	+	+	*M. abscessus subsp. abscessus*	SAMEA3138276	GCF_000069185.1	
		+	+	*M. abscessus subsp. massiliense*	SAMN06064247	GCF_002086375.1	
		+	+	*M. abscessus subsp. bolletii*	SAMN06064233	GCF_002086525.1	
	+	+	+	*M.* sp. LB1	SAMN15472764	GCF_013449725.1	*M.* sp. LB1
			+	*M.* sp. NPDC050853	SAMN41497030	GCF_040653875.1	*M.* sp. LB1
	+	+	+	*M.* sp. CBMA326	SAMN05827291	GCF_009729375.1	*M.* sp. CBMA326
			+	*M.* sp. CBMA271	SAMN12642638	GCF_009729205.1	*M.* sp. CBMA326
M. chitae	+	+	+	*M. chitae*	SAMEA4364211	GCF_900637205.1	
			+	*M. chitae*	SAMD00153176	GCF_010727725.1	
			+	*M. chitae*	SAMN15568994	GCF_025821725.1	

			+	*M. sp. SO_2017_LW2_149*	SAMN39905052	GCF_041656075.1	
	+	+	+	*M. sp. Mu0050*	SAMEA114267541	GCF_963378085.1	*Mu0050*
M. chitae	+	+	+	*M. sp. Mu0053*	SAMEA114267543	GCF_963378095.1	*Mu0053*
	+	+	+	*M. confluentis*	SAMD00153189	GCF_010729895.1	
			+	*M. confluentis*	SAMN04216920	GCF_002102105.1	
			+	*M. confluentis*	SAMN15568936	GCF_025822845.1	
	+	+	+	*M. sp. ACS4331*	SAMN04634265	GCF_001667265.1	*ACS4331*
	+	+	+	*M. palauense*	SAMN07606484	GCF_002592005.1	
	+	+	+	*M. komossense*	SAMN15568956	GCF_025822605.1	
	+	+	+	*M. mengxianglii*	SAMN16862530	GCF_015710575.1	
			+	*M. mengxianglii*	SAMN16833905	GCF_015910525.1	
	+	+	+	*M. tokaiense*	SAMD00153164	GCF_010725885.1	
			+	*M. tokaiense*	SAMN15568996	GCF_025821705.1	
			+	*M. tokaiense*	SAMEA37369168	GCF_900453365.1	
			+	*M. murale*	SAMN15568965	GCF_025822305.1	
			+	*M. murale*	SAMD00153185	GCF_010722995.1	
		+	+	*M. sp. djl-10*	SAMN05415090	GCF_001695755.1	
	+	+	+	*M. sp. MS1601*	SAMN06187687	GCF_001984215.1	*MS1601*
M. insubricum	+	+	+	*M. brumae*	SAMN07733417	GCF_002553575.1	
			+	*M. brumae*	SAMN10734566	GCF_004014795.1	
			+	*M. brumae*	SAMN25131781	GCF_025215495.1	
			+	*M. brumae*	SAMN15568958	GCF_025822525.1	
			+	*M. brumae*	SAMEA3874519	GCF_900073015.1	
	+	+	+	*M. insubricum*	SAMD00153202	GCF_010731615.1	
			+	*M. insubricum*	SAMN15569020	GCF_025821225.1	
			+	*M. insubricum*	SAMN06064241	GCF_002086255.1	
			+	*M. insubricum*	SAMN15687568	GCA_020637095.1	
	+	+	+	*M. fallax*	SAMD00153171	GCF_010726955.1	
			+	*M. fallax*	SAMN04216925	GCF_002101995.1	
			+	*M. fallax*	SAMN15569010	GCF_025821465.1	
M. parafortuitum	+	+	+	*M. sp. BiH015*	SAMN32597634	GCF_027706845.1	*BiH015*
	+	+	+	*M. iranicum*	SAMN04216933	GCF_002101705.1	
			+	*M. iranicum UM_TJL*	SAMN02471859	GCF_000455165.1	
			+	*M. iranicum*	SAMN32036409	GCF_026805495.1	
	+	+	+	*M. iranicum*	SAMN04622670	GCF_001650495.1	*H39*
	**+**	**+**	**+**	*Mycobacterium sp.* pW049	SAMN42688602	GCF_041287165.2	*bulgaricum*
	+	+	+	*M. sp. 236(2023)*	SAMN33926743	GCF_029623955.1	*236(2023)*
	+	+	+	*M. iranicum*	SAMN12025127	GCF_014190915.1	*AT2.18*
	+	+	+	*M. gilvum*	SAMEA4076702	GCF_900454025.1	
			+	*M. gilvum*	SAMN15568987	GCF_025821885.1	
		+	+	*M. gilvum*	SAMN00713568	GCF_000184435.1	
			+	*M. sp. PYR10*	SAMN06858905	GCF_002762075.1	
			+	*M. sp. PAM1*	SAMN19945804	GCF_019148985.1	
	+	+	+	*M. parafortuitum*	SAMN06064253	GCF_002086815.1	*M. parafortuitum.I*
			+	*M. parafortuitum*	SAMEA4681509	GCF_900417285.1	*M. parafortuitum.I*
	+	+	+	*M. parafortuitum*	SAMD00153162	GCF_010725485.1	*M. parafortuitum.II*
			+	*M. sp. EPG1*	SAMN08196607	GCF_002946335.1	*M. parafortuitum.II*

			+	*M. parafortuitum*	SAMN41455849	GCF_040105145.1	*M. parafortuitum.II*
	+	+	+	*M. aurum*	SAMEA3216266	GCF_001049355.1	
			+	*M. aurum*	SAMN15569011	GCF_025821345.1	
			+	*M. aurum*	SAMEA4364210	GCF_900637195.1	
	+	+	+	*M. austroafricanum*	SAMEA3138973	GCF_000612725.1	
			+	*M. austroafricanum*	SAMN20848283	GCF_019880505.1	
			+	*M. austroafricanum*	SAMN08578609	GCF_002968275.1	
			+	*M. austroafricanum*	SAMN17841208	GCF_016919645.1	
			+	*M. austroafricanum*	SAMN19987908	GCF_019856635.1	
			+	*M. austroafricanum*	SAMN19987910	GCF_019856675.1	
			+	*M. vanbaalenii*	SAMN15569003	GCF_025821565.1	
M. parafortuitum			+	*M. vanbaalenii*	SAMN02598347	GCF_000015305.1	
			+	*M. vanbaalenii*	SAMN15486221	GCF_021559935.1	
			+	*M. vanbaalenii*	SAMN33427312	GCF_029168595.1	
		+	+	*M. austroafricanum*	SAMN19987909	GCF_019856655.1	
	+	+	+	*M. vaccae*	SAMN02470132	GCF_000295825.1	
			+	*M. vaccae*	SAMD00046508	GCF_001552715.1	
			+	*M. vaccae*	SAMN03702434	GCF_001655245.1	
			+	*M. vaccae*	SAMN15569021	GCA_025821205.1	
	+	+	+	*M.* sp. NAZ190054	SAMN04227060	GCF_001545925.1	
	+	+	+	*M.* sp. SMC-8	SAMN20167551	GCF_025263565.1	
	+	+	+	*M.* sp. NGTWS1803	SAMD00393830	GCA_026005275.1	*M.* sp. NGTWS1803
			+	*M.* sp. NGTWSNA01	SAMD00322293	GCA_026005235.1	*M.* sp. NGTWS1803
			+	*M.* sp. NGTWS0302	SAMD00393829	GCA_026005255.1	*M.* sp. NGTWS1803
	+	+	+	*M.* sp. CR10	SAMN11431104	GCF_005222675.1	*M.* sp. CR10
M. pyrenivorans	+	+	+	*M. pyrenivorans*	SAMN15568999	GCF_025821625.1	
			+	*M. pyrenivorans*	SAMD00017016	GCA_001314105.1	
	+	+	+	*M. hippocampi*	SAMN14846999	GCF_013390125.1	*M.* sp. DL
	+	+	+	*M. vanbaalenii*	SAMEA5215812	GCF_902705885.1	*M.* sp. BC8_1
	+	+	+	*M. hippocampi*	SAMD00153224	GCF_010723735.1	
	+	+	+	*M. xanthum*	SAMN17081024	GCF_019710635.1	
	+	+	+	*M. poriferae*	SAMD00153179	GCF_010728325.1	
			+	*M. poriferae*	SAMN15568947	GCF_025822745.1	
			+	*M.* sp. THAF 192	SAMN11996133	GCF_009363295.1	
			+	*M.* sp. OfavD-34-C	SAMN25860348	GCF_022348005.1	
	+	+	+	*M.* sp. PO2	SAMD00208488	GCF_017312425.1	*M.* sp. PO2
			+	*M.* sp. PO1	SAMD00208487	GCF_017312405.1	
	+	+	+	*M.* sp. PSTR-4-N	SAMN25860347	GCF_022348085.1	*M.* sp. PSTR-4-N
	+	+	+	*M. obuense*	SAMN02952053	GCF_001044245.1	*M. obuense*
			+	*M. obuense*	SAMEA110089006	GCA_943913265.1	*M. obuense*
			+	*M. obuense*	SAMN03451582	GCF_000974925.2	*M. obuense*
M. chubuense			+	*M.* sp. SWH-M1	SAMN02729863	GCF_001905565.1	*M. obuense*
			+	*M. obuense*	SAMN10799735	GCF_004359005.1	*M. obuense*
		+	+	*M. kyogaense*	SAMN09281965	GCF_003254575.1	*M. obuense*
	+	+	+	*M.* sp. 018/SC-01/001	SAMN12158155	GCF_007096635.1	
	+	+	+	*M. rufum*	SAMN15569019	GCA_025821255.1	
			+	*M. rufum*	SAMN02777056	GCF_000762985.1	

M. chubuense			+	*M. rufum*	SAMN20254267	GCF_022374875.2	
	+	+	+	*M. psychrotolerans*	SAMD00153184	GCF_010729305.1	
			+	*M. psychrotolerans*	SAMN15568990	GCF_025821805.1	
		+	+	*M. sp. Soil538*	SAMN04155656	GCF_001428285.1	
	+	+	+	*M. chubuense*	SAMN06064237	GCF_002086595.1	*M. chubuense*
			+	*M. chubuense*	SAMN03375743	GCF_001044255.1	*M. chubuense*
			+	*M. chubuense*	SAMEA4535747	GCF_900453455.1	*M. chubuense*
		+	+	*M. chlorophenolicum*	SAMD00046518	GCF_001552315.1	*M. chubuense*
			+	*M. rufum*	SAMN15436697	GCA_016219585.1	*M. chubuense*
			+	*M. chlorophenolicum*	SAMN03327461	GCF_001044235.1	*M. chubuense*
		+	+	*M. sp. BK558*	SAMN10871991	GCF_004216875.1	*M. chubuense*
M. duvalii	+	+	+	*M.* sp. pV006	SAMN42692130	GCF_041287115.1	*M. sibiricum*
	+	+	+	*M. duvalii*	SAMD00153169	GCF_010726645.1	
			+	*M. duvalii*	SAMN07710579	GCF_002553585.1	
			+	*M. duvalii*	SAMN15568930	GCF_025823005.1	
	+	+	+	*M. sp. SMC-4*	SAMN20167676	GCF_025263265.1	
M. doricum	+	+	+	*M. manitobense*	SAMN15568997	GCF_025821665.1	*M. manitobense*
		+	+	*M. hippophais*	SAMN32338864	GCF_027529605.1	*M. manitobense*
		+	+	*M. arseniciresistens*	SAMN36451562	GCF_030519285.1	*M. manitobense*
	+	+	+	*M.* sp. NPDC050551	SAMN41497484	GCF_040674075.1	NPDC050551
	+	+	+	*M. sp. F2034L*	SAMN26635178	GCF_023015925.1	*M.* sp. *F2034L*
			+	*M. sp. 852013-51886_SCH5428379*	SAMN04691969	GCF_001665575.1	*M.* sp. *F2034L*
	+	+	+	*M. gossypii*	SAMN17805296	GCF_017352375.1	
	+	+	+	*M. sp. PS03-16*	SAMN11249675	GCF_004570325.1	*PS03-16*
	+	+	+	*M. sp. GA-2829*	SAMN04349500	GCF_001499965.1	*GA-2829*
	+	+	+	*M. litorale*	SAMD00200502	GCF_014218295.1	*M.* sp. NIID-NTM18
	+	+	+	*M. litorale*	SAMD00153207	GCF_010731695.1	
			+	*M. litorale*	SAMN05421873	GCF_004366555.1	
			+	*M. litorale*	SAMN15568914	GCF_025823345.1	
	+	+	+	*M. baixiangningiae*	SAMN17013278	GCF_016313185.1	
			+	*M. baixiangningiae*	SAMN15770944	GCF_014893035.1	
		+	+	*M. baixiangningiae*	SAMN16833901	GCF_015910495.1	
	+	+	+	*M. doricum*	SAMD00153178	GCF_010728155.1	
			+	*M. doricum*	SAMN04216922	GCF_002102115.1	
			+	*M. doricum*	SAMN15568951	GCF_025822585.1	
	+	+	+	*M. monacense*	SAMN06064249	GCF_002086655.1	
			+	*M. monacense*	SAMN10880098	GCF_009931355.1	
			+	*M. monacense*	SAMD00153199	GCF_010731575.1	
			+	*M. monacense*	SAMN32724495	GCF_027886265.1	
			+	*M. monacense*	SAMN04856196	GCF_001665825.1	
			+	*M. monacense*	SAMN04696138	GCF_001667465.1	
			+	*M. obuense*	SAMN13165604	GCF_014194265.1	
	+	+	+	*M. sp. IS-1496*	SAMN04349501	GCF_001499995.1	
	+	+	+	*M. sp. IS-1742*	SAMN04349502	GCF_001500125.1	
M. agri	+	+	+	*M. sp. 120270*	SAMN38084522	GCF_033741175.1	
	+	+	+	*M. sp. ITM-2016-00318*	SAMN08578608	GCF_002968285.1	

	+	+	+	*M. stellerae*	SAMN10102143	GCF_003719305.1	
	+	+	+	*M. sp. 1274761. 0*	SAMN04634189	GCF_001668615.1	
M. agri	+	+	+	*M. agri*	SAMN07733415	GCF_002553505.1	
			+	*M. agri*	SAMD00153167	GCF_010722915.1	
	+	+	+	*M. hubeiense*	SAMN08116703	GCF_002838065.1	
	+	+	+	*M. sp. ACS1612*	SAMN04634262	GCF_001667505.1	
	+	+	+	*M. sp. 3519A*	SAMEA104417301	GCF_900240945.1	
	**+**	**+**	**+**	*M.* sp. NPDC048908	SAMN43996259	GCA_043197955.1	
	+	+	+	*M. sp. OAS707*	SAMN13190077	GCF_014873705.1	*M.* sp. OAS707
			+	*M. sp. OAE908*	SAMN18259016	GCF_017872775.1	*M.* sp. OAS707
	+	+	+	*M. sp. JS623*	SAMN02261384	GCF_000328565.1	
	+	+	+	*M. moriokaense*	SAMN08778298	GCF_003201655.1	*M.* sp. GAS496
M. gadium	+	+	+	*M. gadium*	SAMD00153182	GCF_010728925.1	
		+	+	*M. gadium*	SAMN26536104	GCF_029675005.1	
	+	+	+	*M. tusciae*	SAMN06064262	GCF_002086795.1	
	+	+	+	*M. gallinarum*	SAMD00153170	GCF_010726765.1	
			+	*M. gallinarum*	SAMN27915967	GCF_032883055.1	
	+	+	+	*M. tusciae*	SAMN02256521	GCF_000243415.2	*M.* sp. JS617
	+	+	+	*M. neglectum*	SAMN07606483	GCF_002591975.1	
	+	+	+	*M. rhodesiae*	SAMN02261383	GCF_000230895.2	*M.* sp. NBB3
	+	+	+	*M. barrassiae*	SAMN15568944	GCF_025822765.1	
	+	+	+	*M. moriokaense*	SAMN06064251	GCF_002086395.1	
			+	*M. moriokaense*	SAMD00153165	GCF_010726085.1	
			+	*M. moriokaense*	SAMN15568977	GCF_025822185.1	
	+	+	+	*M. deserti*	SAMN30822140	GCF_025345615.1	
M. elephantis	+	+	+	*M. pulveris*	SAMD00153163	GCF_010725725.1	
			+	*M. pulveris*	SAMN15568975	GCF_025822105.1	
	+	+	+	*M. elephantis*	SAMN10734567	GCF_004014805.1	
			+	*M. elephantis*	SAMN15568955	GCF_025822495.1	
		+	+	*M. elephantis*	SAMN06064238	GCF_002086605.1	
			+	*M. elephantis*	SAMN03580702	GCF_001005175.1	
			+	*M. elephantis*	SAMN04634166	GCF_001665365.1	
			+	*M. elephantis*	SAMN04691997	GCF_001665755.1	
			+	*M. elephantis*	SAMN04696136	GCF_001667535.1	
	+	+	+	*M. holsaticum*	SAMN20254262	GCF_019645835.1	
			+	*M. holsaticum*	SAMN05729959	GCF_001722355.1	
			+	*M. holsaticum*	SAMN15569023	GCF_022558565.1	
			+	*M. holsaticum*	SAMN32724496	GCF_027886245.1	
M. flavescens	+	+	+	*M. phlei*	SAMN04481318	GCF_009192875.1	
			+	*M. phlei*	SAMN04537322	GCF_001582015.1	
			+	*M. phlei*	SAMN02470133	GCF_000257725.1	
			+	*M. phlei*	SAMN04537321	GCF_001581995.1	
			+	*M. phlei*	SAMN04537320	GCF_001582005.1	
			+	*M. phlei*	SAMN02951394	GCF_001582025.1	
			+	*M. phlei*	SAMN15860745	GCF_015333575.1	
			+	*M. phlei*	SAMN28729052	GCF_024652765.1	
			+	*M. phlei*	SAMEA37370668	GCF_900453675.1	

	+	+	+	*M. salfingeri*	SAMN20675999	GCF_022317005.1	
	+	+	+	*M.* sp. B14F4	SAMN41446672	GCF_039871645.1	
	+	+	+	*M. rutilum*	SAMN04489835	GCF_900108565.1	
	+	+	+	*M. flavescens*	SAMN15568943	GCF_025822705.1	
			+	*M. flavescens*	SAMN05729960	GCF_001722335.1	
	+	+	+	*M. sp. IS-3022*	SAMN04349494	GCF_001500065.1	
	+	+	+	*M. sp. GA-1999*	SAMN04349492	GCF_001500145.1	*M.* sp. GA-1999
			+	*M. sp. GA-0227b*	SAMN04349491	GCF_001499855.1	*M.* sp. GA-1999
			+	*M. sp. IS-1556*	SAMN04349493	GCF_001499985.1	*M.* sp. GA-1999
	+	+	+	*M. celeriflavum*	SAMN06064236	GCF_002086175.1	
			+	*M. celeriflavum*	SAMD00153212	GCF_010731795.1	
			+	*M. celeriflavum*	SAMN15568950	GCF_025822625.1	
			+	*M. celeriflavum*	SAMN04692011	GCF_001667065.1	
	+	+	+	*M. sp. E740*	SAMN04634201	GCF_001672895.1	
	+	+	+	*M. sp. GF69*	SAMN09476218	GCF_003284965.1	
	+	+	+	*M.* sp. ND9-15	SAMN34339285	GCF_035918395.1	
	+	+	+	*M. sp. 1164985.4*	SAMN04634188	GCF_001668575.1	
	+	+	+	*M. sp. 852002-51961_SCH5331710*	SAMN04691954	GCF_001665535.1	*M.* sp. SCH5331710
		+	+	*M. sp. E136*	SAMN04634236	GCF_001666865.1	*M.* sp. SCH5331710
	+	+	+	*M. komaniense*	SAMEA3432687	GCF_001050035.1	
M. flavescens	+	+	+	*M. malmesburyense*	SAMEA3432686	GCF_001050015.1	
	+	+	+	*M. sp. GA-1285*	SAMN04349499	GCF_001499905.1	
	+	+	+	*M. neumannii*	SAMN07269033	GCF_002245615.1	*M. neumannii*
			+	*M. flavescens*	SAMEA4076703	GCF_900637135.1	*M. neumannii*
		+	+	*M. lehmannii*	SAMN07269029	GCF_002245535.1	*M. neumannii*
			+	*M. lehmannii*	SAMN04349498	GCF_001499925.1	*M. neumannii*
			+	*M. acapulense*	SAMEA4022018	GCF_900089125.1	*M. neumannii*
			+	*M. acapulense*	SAMN04349495	GCF_001499915.1	*M. neumannii*
			+	*M. acapulense*	SAMN04349497	GCF_001500085.1	*M. neumannii*
			+	*M. acapulense*	SAMN04349496	GCF_001500105.1	*M. neumannii*
		+	+	*M. sp. 852002-51152_SCH6134967*	SAMN04696151	GCF_001667105.1	*M. neumannii*
		+	+	*M. sp. 852014-52144_SCH5372336*	SAMN04691977	GCF_001665615.1	*M. neumannii*
	+	+	+	*M. novocastrense*	SAMD00041175	GCF_001570485.1	
			+	*M. novocastrense*	SAMN15568972	GCF_025822165.1	
		+	+	*M. novocastrense*	SAMN04349490	GCF_001499845.1	
			+	*M. novocastrense*	SAMN04349489	GCF_001499825.1	
			+	*M. novocastrense*	SAMN04339709	GCF_001499835.1	
		+	+	*M. sp. GA-1199*	SAMN04349504	GCF_001500045.1	
	+	+	+	*M. sp. IS-1590*	SAMN04349503	GCF_001500025.1	*IS-1590*
	+	+	+	*M. hassiacum*	SAMEA4888736	GCF_900603025.1	
			+	*M. hassiacum*	SAMN02256416	GCF_000379865.1	
			+	*M. hassiacum*	SAMN02471699	GCF_000300375.1	
			+	*M. hassiacum*	SAMN32724494	GCF_027886095.1	
	+	+	+	*M. thermoresistibile*	SAMN02471087	GCF_000234585.1	
			+	*M. thermoresistibile*	SAMD00041176	GCF_001570505.1	

			+	*M. thermoresistibile*	SAMN15568963	GCF_025822325.1	
			+	*M. thermoresistibile*	SAMEA4412656	GCF_900187065.1	
M. sediminis	+	+	+	*M. sp. Root135*	SAMN04155726	GCF_001426545.1	
	+	+	+	*M. hodleri*	SAMN10176386	GCF_006864685.1	*M.* sp. ColchesterB
		+	+	*M. sp. AT1*	SAMN06349362	GCF_002043095.1	*M.* sp. ColchesterB
	+	+	+	*M. sp. P1-18*	SAMN07447407	GCF_008329535.1	
	+	+	+	*M. hodleri*	SAMN15568969	GCF_025822245.1	
	+	+	+	*M.* sp. NPDC006124	SAMN41498424	GCF_040614235.1	
	+	+	+	*M. yunnanensis*	SAMN15568918	GCF_025823185.1	
	+	+	+	*M. sp. TUM20985*	SAMD00573267	GCF_030295745.1	
	+	+	+	*M. hodleri*	SAMN10244993	GCF_006439015.1	*M.* sp. S5.20
	+	+	+	*M. madagascariense*	SAMD00153188	GCF_010729665.1	
			+	*M. madagascariense*	SAMN15568976	GCF_025822085.1	
	+	+	+	*M. sp. 050158*	SAMN38084454	GCF_033741125.1	*M.* sp. 50158
	+	+	+	*M. sp. URHB0044*	SAMN02743948	GCF_000620625.1	
	+	+	+	*M. sp. AZCC_0083*	SAMN14915993	GCF_014202335.1	
	+	+	+	*M. sp. P9-64*	SAMN07447410	GCF_008329605.1	
	+	+	+	*M. sp. RTGN3*	SAMN31486590	GCF_033804625.1	
	+	+	+	*M. sp. RTGN4*	SAMN31488203	GCF_033804635.1	
	+	+	+	*M. sp. CAU1645*	SAMN28907230	GCF_024320865.1	
	+	+	+	*M. sp. YH-1*	SAMN24737423	GCF_022557175.1	
	+	+	+	*M. grossiae*	SAMN12660232	GCF_008329645.1	
			+	*M. grossiae*	SAMN05465167	GCF_001766635.1	
	+	+	+	*M. sediminis*	SAMD00153209	GCF_010731735.1	
			+	*M. sediminis*	SAMN15568932	GCF_025822985.1	
	+	+	+	*M. lacusdiani*	SAMN23132885	GCF_021916785.1	
	+	+	+	*M. arabiense*	SAMD00153213	GCF_010731815.2	
			+	*M. arabiense*	SAMN15568925	GCF_025823225.1	
	+	+	+	*M.* sp. GCM10028919	SAMN43284529	GCF_042666295.1	
M. mucogenicum	+	+	+	*M. sp. TY81*	SAMD00239442	GCF_018326285.1	*M.* sp. TY81
			+	*M. sp. TY66*	SAMD00237929	GCF_018326145.1	*M.* sp. TY81
		+	+	*M. sp.* 141076	SAMN38084549	GCF_033741115.1	*M.* sp. TY81
		+	+	*M.* sp. SM3041	SAMN39413575	GCF_039668335.1	*M.* sp. TY81
	+	+	+	*M. mucogenicum*	SAMN08339096	GCF_005670685.2	
			+	*M. mucogenicum*	SAMN13067489	GCF_009192845.1	
			+	*M. mucogenicum*	SAMEA3535282	GCF_001291445.1	
			+	*M. sp. 360MFTsu5.1*	SAMN02440518	GCF_000383495.1	
			+	*M. sp. UNC410CL29Cvi84*	SAMN02743380	GCF_000686745.1	
			+	*M. sp. 283mftsu*	SAMN04488580	GCF_900107555.1	
		+	+	*M. mucogenicum*	SAMN04634163	GCF_001668705.1	
	+	+	+	*M. phocaicum*	SAMD00153196	GCF_010731115.1	
			+	*M. phocaicum*	SAMN08339097	GCF_005670655.1	
		+	+	*M. mucogenicum*	SAMN04299488	GCF_001557045.1	
		+	+	*M. mucogenicum*	SAMN04634180	GCF_001665375.1	
		+	+	*M. mucogenicum*	SAMN10799736	GCF_004359045.1	
		+	+	*M. sp. NCC-Tsukiji*	SAMD00117553	GCF_003851485.1	
		+	+	*M. mucogenicum*	SAMN31811489	GCF_026501155.1	

		+	+	*M. phocaicum*	SAMN22157750	GCF_020520165.1	
	+	+	+	*M. mucogenicum*	SAMN31811490	GCF_026501225.1	*M.* sp. 21IE208
M. mucogenicum	+	+	+	*M. sp. CBMA226*	SAMN12642641	GCF_009729075.1	*M.* sp. *CBMA226*
	+	+	+	*M. llatzerense*	SAMN04954894	GCF_025331195.1	
		+	+	*M. llatzerense*	SAMN03295723	GCF_000878195.1	
			+	*M. sp. UNC280MFTsu5.1*	SAMN02745509	GCF_000746215.1	
			+	*M. llatzerense*	SAMN04955244	GCF_025331235.1	
		+	+	*M. llatzerense*	SAMN04004267	GCF_001865165.1	
	+	+	+	*M. sp. ST-F2*	SAMN02729876	GCF_001905655.1	
	+	+	+	*M. aubagnense*	SAMN08339098	GCF_005670695.1	
			+	*M. aubagnense*	SAMD00153195	GCF_010730955.1	
	+	+	+	*M. sp. CBMA234*	SAMN06011858	GCF_009729415.1	*M.* sp. *CBMA234*
		+	+	*M. sp. CBMA213*	SAMN06579321	GCF_009729395.1	*M.* sp. *CBMA234*
			+	*M. sp. CBMA311*	SAMN12642643	GCF_009729085.1	*M.* sp. *CBMA234*
			+	*M. sp. CBMA360*	SAMN12642644	GCF_009729105.1	*M.* sp. *CBMA234*
			+	*M. sp. CBMA335*	SAMN12642633	GCF_009729275.1	*M.* sp. *CBMA234*
			+	*M. sp. CBMA230*	SAMN12642631	GCF_009729295.1	*M.* sp. *CBMA234*
			+	*M. sp. CBMA293*	SAMN12642632	GCF_009729335.1	*M.* sp. *CBMA234*
	**+**	**+**	**+**	*M.* sp. MAA66	SAMN41528301	GCF_041261665.1	
	+	+	+	*M. sp. OTB74*	SAMN28057932	GCF_029892685.1	
M. neoaurum	+	+	+	*M. sp. Root265*	SAMN04155773	GCF_001428895.1	
	+	+	+	*M. sp. P9-22*	SAMN07447409	GCF_008329585.1	
	+	+	+	*M. frederiksbergense*	SAMN15568973	GCF_025822145.1	
			+	*M. frederiksbergense*	SAMN10915336	GCF_012223425.1	
		+	**+**	*M. frederiksbergense*	SAMN36403483	GCF_030499155.1	
		+	+	*M. sp. ITM-2016-00316*	SAMN08578606	GCF_002968335.1	
	+	+	+	*M. sp. MBM*	SAMN25085286	GCF_021648845.1	
	+	+	+	*M. adipatum*	SAMN04959083	GCF_001644575.1	
	+	+	+	*M. aurum*	SAMEA3914648	GCF_900078775.1	*M.* sp. *SAMEA3914648*
	+	+	+	*M. diernhoferi*	SAMN20254259	GCF_019456655.1	
			+	*M. diernhoferi*	SAMN15568961	GCF_025822385.1	
			+	*M. diernhoferi*	SAMN07710578	GCF_002553495.1	
			+	*M. diernhoferi*	SAMN06034711	GCF_001907655.1	
			+	*M. diernhoferi*	SAMN05729958	GCF_002024975.1	
	+	+	+	*M. bacteremicum*	SAMN06064232	GCF_002086115.1	
			+	*M. bacteremicum*	SAMN15568916	GCF_025823285.1	
	+	+	+	*M. neoaurum*	SAMN32364481	GCA_027627515.1	*M. neoaurum.II*
			+	*M. neoaurum*	SAMN32362212	GCA_027857015.1	*M. neoaurum.II*
			+	*M. sp. VKMAc-1816D*	SAMN02469804	GCF_000416385.1	*M. neoaurum.II*
			+	*M. neoaurum*	SAMN02641502	GCF_000317305.3	*M. neoaurum.II*
			+	*M. neoaurum*	SAMN03329538	GCF_000959045.1	*M. neoaurum.II*
			+	*M. neoaurum*	SAMN04386425	GCF_001510785.1	*M. neoaurum.II*
			+	*M. neoaurum*	SAMN09754462	GCF_003367335.1	*M. neoaurum.II*
	+	+	+	*M. neoaurum*	SAMN02729281	GCF_000691525.1	*M. neoaurum.I*
			+	*M. neoaurum*	SAMEA3139061	GCF_000724065.1	*M. neoaurum.I*
			+	*M. neoaurum*	SAMN08339099	GCF_005670605.1	*M. neoaurum.I*

M. neoaurum			+	*M. sp. UNCCL9*	SAMN02745506	GCF_000744355.1	*M. neoaurum.I*
			+	*M. neoaurum*	SAMN18915540	GCF_018363015.1	*M. neoaurum.I*
			+	*M. neoaurum*	SAMN25687150	GCF_022559925.1	*M. neoaurum.I*
			+	*M. neoaurum*	SAMN25687151	GCF_022559965.1	*M. neoaurum.I*
			+	*M. neoaurum*	SAMN04488581	GCF_900101555.1	*M. neoaurum.I*
	+	+	+	*M. fluoranthenivorans*	SAMN12024888	GCF_011758805.1	*M. fluranthenivorans*
			+	*M. fluoranthenivorans*	SAMN15568934	GCF_025823075.1	*M. fluranthenivorans*
		+	+	*M. hackensackense*	SAMN15568952	GCF_025822555.1	*M. fluranthenivorans*
			+	*M. fluranthenivorans*	SAMN15486235	GCF_014295435.1	*M. fluranthenivorans*
			+	*M. fluranthenivorans*	SAMN02799620	GCF_900100615.1	*M. fluranthenivorans*
	+	+	+	*M. sp. 120266*	SAMN38084519	GCF_033741155.1	
	+	+	+	*M. sp. J2*	SAMN31599172	GCF_026240945.1	
	+	+	+	*M. canariasense*	SAMN04216915	GCF_002101555.1	
			+	*M. canariasense*	SAMD00041173	GCF_001570445.1	
			+	*M. canariasense*	SAMN15568964	GCF_025822345.1	
	+	+	+	*M. cosmeticum*	SAMEA3138996	GCF_000613185.1	
			+	*M. cosmeticum*	SAMN08339100	GCF_005670675.1	
			+	*M. sp. UM_RHS*	SAMN02471836	GCF_000455185.1	
			+	*M. sp. UM_NYF*	SAMN03493514	GCF_000987455.1	
		+	+	*M. cosmeticum*	SAMN39413577	GCF_039668325.1	
M. smegmatis	+	+	+	*M. goodii*	SAMN20254253	GCF_022370755.2	
			+	*M. sp.*	SAMN02729875	GCF_001905305.1	
			+	*M. goodii*	SAMN07828250	GCF_002798385.1	
			+	*M. goodii*	SAMN19031646	GCF_018524265.1	
			+	*M. goodii*	SAMN19031644	GCF_018524285.1	
			+	*M. goodii*	SAMN19031645	GCF_018524295.1	
			+	*M. goodii*	SAMN19031643	GCF_018524325.1	
			+	*M. goodii*	SAMN19031642	GCF_018524375.1	
	+	+	+	*M. goodii*	SAMN03839023	GCF_001187505.1	*M.* sp. X7B
	+	+	+	*M. smegmatis*	SAMEA2517361	GCF_001457595.1	
			+	*M. smegmatis*	SAMN02603982	GCF_000015005.1	
			+	*M. smegmatis*	SAMN02603392	GCF_000283295.1	
			+	*M. smegmatis*	SAMN02472105	GCF_000331165.2	
			+	*M. smegmatis*	SAMN02951866	GCF_000521845.1	
			+	*M. smegmatis*	SAMN03068201	GCF_000767605.1	
M. brisbanense	+	+	+	*M. dioxanotrophicus*	SAMN06134492	GCF_002157835.1	
	+	+	+	*M. aquaticum*	SAMN06064228	GCF_002086485.1	
	+	+	+	*M. brisbanense*	SAMN15568968	GCF_025822265.1	
			+	*M. brisbanense*	SAMD00041172	GCF_001570425.1	
		+	+	*M. sp. UM_WWY*	SAMN02471848	GCF_000455145.1	
	+	+	+	*M. sp. 852013-50091_SCH5140682*	SAMN04696145	GCF_001665685.1	*M.* sp. SCH5140682
		+	+	*M.* sp. 120322	SAMN38286614	GCF_036409095.1	*M.* sp. SCH5140682
	+	+	+	*M. sp. 155.00*	SAMN02441709	GCF_000373905.1	
	+	+	+	*M. sp. 141.00*	SAMN02441653	GCF_000382405.1	
	+	+	+	*M. sp. 21AC1*	SAMN27915968	GCF_032883035.1	
	+	+	+	*M. wolinskyi*	SAMN04216957	GCF_002101965.1	

			+	*M. wolinskyi*	SAMN15568954	GCF_025822475.1	
			+	*M. wolinskyi*	SAMN15568953	GCF_025822535.1	
		+	+	*M. wolinskyi*	SAMN04113082	GCF_001545135.1	
	+	+	+	*M. mageritense*	SAMEA3138978	GCF_000612825.1	
			+	*M.* sp. DSM 3803	SAMN17589965	GCA_016995035.1	
			+	*M.* sp. SWH-M3	SAMN02729864	GCF_001907615.1	
			+	*M. mageritense*	SAMD00153174	GCF_010727475.1	
			+	*M. mageritense*	SAMD00414014	GCF_020884715.1	
			+	*M. mageritense*	SAMN23076627	GCF_020907445.1	
M. fortuitum	+	+	+	*M. frederiksbergense*	SAMN28058330	GCF_029893345.1	*M.* sp. AC80
	+	+	+	*M. fortunisiensis*	SAMN12436392	GCF_019050325.1	
	+	+	+	*M.* sp. E802	SAMN04634237	GCF_001666875.1	
	+	+	+	*M.* sp. GA-1841	SAMN04634278	GCF_001954135.1	*M.* sp. GA-1841
			+	*M.* sp. NS-7484	SAMN04634271	GCF_001953975.1	*M.* sp. GA-1841
	+	+	+	*M. houstonense*	SAMN15568919	GCF_025823315.1	*M. houstonense*
			+	*M. houstonense*	SAMEA3913388	GCF_900078665.2	*M. houstonense*
		+	+	*M. farcinogenes*	SAMN15569018	GCA_025821245.1	*M. houstonense*
	+	+	+	*M.* sp. HK-90	SAMN35980692	GCF_030486405.1	
	+	+	+	*M.* sp. CBMA294	SAMN12642634	GCF_009729315.1	*M.* sp. CBMA294
			+	*M.* sp. CBMA297	SAMN12642642	GCF_009729095.1	*M.* sp. CBMA294
			+	*M.* sp. CBMA329	SAMN12642636	GCF_009729185.1	*M.* sp. CBMA294
			+	*M.* sp. CBMA331	SAMN12642639	GCF_009729195.1	*M.* sp. CBMA294
			+	*M.* sp. CBMA334	SAMN12642637	GCF_009729215.1	*M.* sp. CBMA294
			+	*M.* sp. CBMA295	SAMN12642635	GCF_009729305.1	*M.* sp. CBMA294
	+	+	+	*M.* sp. DL440	SAMN13155069	GCF_011745145.1	
	+	+	+	*M.* sp. NPDC050441	SAMN41497477	GCF_040655355.1	
	+	+	+	*M.* sp. NPDC049093	SAMN41497458	GCA_040704635.1	
	+	+	+	*M. peregrinum*	SAMN04216946	GCF_002102345.1	
			+	*M. peregrinum*	SAMN10532552	GCF_004721035.1	
			+	*M. peregrinum*	SAMEA3534845	GCF_001403655.1	
			+	*M. peregrinum*	SAMN06702994	GCF_002198105.1	
			+	*M. peregrinum*	SAMN10532378	GCF_004721025.1	
			+	*M. peregrinum*	SAMN15568960	GCF_025822425.1	
		+	+	*M. peregrinum*	SAMN04634257	GCF_001667205.1	
			+	*M. peregrinum*	SAMN04696139	GCF_001665625.1	
		+	+	*M. peregrinum*	SAMN04696154	GCF_001665785.1	
	+	+	+	*M. fortuitum subsp. fortuitum*	SAMD00436749	GCF_022179545.1	
			+	*M. fortuitum subsp. fortuitum*	SAMN02470125	GCF_000295855.1	
			+	*M. fortuitum subsp. fortuitum*	SAMN04295031	GCA_001475495.2	
			+	*M. fortuitum*	SAMEA3444530	GCF_001050075.1	
			+	*M. fortuitum*	SAMN23774624	GCA_021249325.1	
			+	*M.* sp. VKMAc-1817D	SAMN02469803	GCF_000416365.2	
			+	*M. fortuitum*	SAMN15568970	GCF_025822205.1	
			+	*M. fortuitum*	SAMEA37366918	GCF_900453945.1	
			+	*M. fortuitum*	SAMN04692001	GCF_001665455.1	
			+	*M. fortuitum*	SAMN04691961	GCF_001665545.1	
			+	*M. fortuitum*	SAMN04691959	GCF_001665795.1	

		+	+	*M. fortuitum*	SAMN15806909	GCF_020227715.1	
			+	*M. fortuitum*	SAMN04634203	GCF_001665315.1	
			+	*M. fortuitum*	SAMN04634204	GCF_001672835.1	
			+	*M. fortuitum*	SAMN04634205	GCF_001672855.1	
			+	*M. fortuitum*	SAMN04634170	GCF_001673325.1	
			+	*M. fortuitum*	SAMN04634272	GCF_001954035.1	
			+	*M. fortuitum*	SAMN04634280	GCF_001954145.1	
			+	*M. fortuitum*	SAMN13503960	GCF_013138425.1	
			+	*M.* sp. GESEQ-9	SAMN17922829	GCF_017948465.1	
		+	+	*M. fortuitum*	SAMN04692022	GCF_001667345.1	
	+	+	+	*M. lutetiense*	SAMN17673454	GCF_017876775.1	
	+	+	+	*M. alvei*	SAMD00153173	GCF_010727325.1	
			+	*M. alvei*	SAMN15568967	GCF_025822365.1	
	+	+	+	*M. setense*	SAMN01174788	GCF_000805385.1	
			+	*M. setense*	SAMN15569006	GCF_025821545.1	
			+	*M. setence*	SAMN03203477	GCF_000805375.1	
			+	*M. setense*	SAMN04691993	GCF_001665445.1	
			+	*M. setense*	SAMEA104376285	GCF_900236745.1	
	+	+	+	*M.* sp. URHD0025	SAMN02440855	GCF_000426065.1	
	+	+	+	*M. syngnathidarum*	SAMN05897976	GCF_001942625.1	
			+	*M. syngnathidarum*	SAMN05897977	GCF_001853525.1	
			+	*M.* sp. CnD-18-1	SAMN25860346	GCF_022348025.1	
		+	+	*M.* sp. DBP42	SAMN11043092	GCF_005924235.1	
	+	+	+	*M. senegalense*	SAMN20254268	GCF_019645875.1	*M. senegalense*
M. fortuitum			+	*M. senegalense*	SAMN03699825	GCF_001012795.1	*M. senegalense*
			+	*M. senegalense*	SAMN03744420	GCF_001021425.1	*M. senegalense*
			+	*M. senegalense*	SAMN15568935	GCF_025822895.1	*M. senegalense*
			+	*M. conceptionense*	SAMN04216919	GCF_002102065.1	*M. senegalense*
			+	*M. conceptionense*	SAMEA3305051	GCF_001052995.1	*M. senegalense*
			+	*M. conceptionense*	SAMN03793182	GCF_001077745.1	*M. senegalense*
			+	*M. conceptionense*	SAMN04634256	GCF_001665335.1	*M. senegalense*
	+	+	+	*M. neworleansense*	SAMEA3481574	GCF_001245615.1	
			+	*M. neworleansense*	SAMN15568929	GCF_025823025.1	
	+	+	+	*M. septicum*	SAMEA2272699	GCF_000455325.1	*M. septicum*
			+	*M. septicum*	SAMN14517851	GCF_012396425.1	*M. septicum*
		+	+	*M. nivoides*	SAMN10393184	GCF_003855255.1	*M. septicum*
			+	*M.* sp. 455mf	SAMN04488582	GCF_900113075.1	*M. septicum*
			+	*M. boenickei*	SAMN17844880	GCF_016919325.1	*M. septicum*
			+	*M. septicum*	SAMN17925406	GCF_017052695.1	*M. septicum*
			+	*M.* sp. 88mf	SAMN04488583	GCF_900110825.1	*M. septicum*
		+	+	*M. septicum*	SAMN17844879	GCF_016919345.1	*M. septicum*
		+	+	*M. septicum*	SAMN32153154	GCF_028613735.1	*M. septicum*
		+	+	*M. septicum*	SAMN33749009	GCF_029270025.1	*M. septicum*
	+	+	+	*M.* sp. DL99	SAMN10989809	GCF_004762045.1	
	+	+	+	*M.* sp. 050232	SAMN39409527	GCF_036235365.1	
	+	+	+	*M. porcinum*	SAMN06199452	GCF_001942045.1	*M.* sp. HMC1
	+	+	+	*M. porcinum*	SAMN15568926	GCF_025823105.1	

M. fortuitum			+	*M. porcinum*	SAMN06064256	GCF_002086835.1	
			+	*M. vulneris*	SAMEA3138981	GCF_000612885.1	
			+	*M.* sp. AC S5020	SAMN04634259	GCF_001686755.1	
			+	*M. porcinum*	SAMN12356590	GCF_007786435.1	
			+	*M. porcinum*	SAMEA104585265	GCF_900289185.1	
		+	+	*M. porcinum*	SAMN05730077	GCF_001722425.1	
		+	+	*M. porcinum*	SAMN04634260	GCF_001686835.1	
			+	*M. porcinum*	SAMN04634255	GCF_001686675.1	
			+	*M. porcinum*	SAMN04634267	GCF_001686845.1	
	+	+	+	*M. boenickei*	SAMN07733416	GCF_002553535.1	
			+	*M. boenickei*	SAMD00153197	GCF_010731295.1	
			+	*M. boenickei*	SAMN15569026	GCF_022558525.1	
			+	*M. boenickei*	SAMEA92028418	GCF_900166915.1	
orphan	+	+	+	*M.* sp. MYCO198283	SAMN21161762	GCF_021213805.1	
M. sphagni	+	+	+	*M. sphagni*	SAMN11637231	GCF_013337765.1	*M.* sp. ENV482
	+	+	+	*M. sp. RTGN5*	SAMN31488241	GCF_033804465.1	
	+	+	+	*M. helvum*	SAMD00153218	GCF_010731895.1	
	+	+	+	*M. sarraceniae*	SAMD00153217	GCF_010731875.1	
	+	+	+	*M. sp. BK086*	SAMN10872004	GCF_004362315.1	*M.* sp. BK086
			+	*M. sp. BK556*	SAMN12024151	GCF_014195025.1	*M.* sp. BK086
			+	*M. sp. BK607*	SAMN12024104	GCF_014195125.1	*M.* sp. BK086
			+	*M. sp. BK634*	SAMN12024136	GCF_014195195.1	*M.* sp. BK086
	+	+	+	*M. vinylchloridicum*	SAMN14942828	GCF_013404075.1	
	+	+	+	*M. rhodesiae*	SAMN02256528	GCF_000230935.1	*M.* sp. JS60
	+	+	+	*M. sphagni*	SAMN07372526	GCF_002250655.1	
			+	*M. sphagni*	SAMN15569004	GCF_025821515.1	
	+	+	+	*M. pinniadriaticum*	SAMN31656079	GCF_026242035.1	
			+	*M. sp. CVI_P4*	SAMN31656080	GCF_026242045.1	
	+	+	+	*M. sp. shizuoka-1*	SAMD00096763	GCF_002723835.1	
	+	+	+	*M. sp. EPa45*	SAMN03758429	GCF_001021385.1	
	+	+	+	*M. sp. P1-5*	SAMN07447405	GCF_008329565.1	
	+	+	+	*M. pallens*	SAMN20254255	GCF_019456675.1	*M. pallens*
		+	+	*M. sp. WY10*	SAMN06011242	GCF_001886515.1	*M. pallens*
			+	*M. sp. PYR15*	SAMN06858920	GCF_002335685.1	*M. pallens*
		+	+	*M. crocinum*	SAMN20254251	GCA_022370635.2	*M. pallens*
			+	*M. crocinum*	SAMN15568959	GCF_025822435.1	*M. pallens*
	+	+	+	*M. sp. ENV421*	SAMN07774437	GCF_002887815.1	
	+	+	+	*M. aromaticivorans*	SAMN02585141	GCF_000559085.1	
	+	+	+	*M. rhodesiae*	SAMN06064257	GCF_002086695.1	
			+	*M. rhodesiae*	SAMN15568940	GCF_025822805.1	
	+	+	+	*M. sp. ELW1*	SAMN09980424	GCF_008329905.1	*M.* sp. ELW1
	+	+	+	*M. aichiense*	SAMEA4076713	GCF_900453085.1	
			+	*M. aichiense*	SAMD00153166	GCF_010726245.1	
			+	*M. aichiense*	SAMN15568971	GCF_025822215.1	
	+	+	+	*M. litorale*	SAMN06339708	GCF_002007745.1	*M.* sp. F4
	+	+	+	*M. sp. CH28*	SAMN10867835	GCF_004745805.1	
	+	+	+	*M. litorale*	SAMN21385979	GCF_022760805.1	*M. alkanivorans*

	+	+	+	*M. massilipolynesiensis*	SAMEA3696413	GCF_001494595.1	
	+	+	+	*M. anyangense*	SAMD00153216	GCF_010731855.1	
M. sphagni	+	+	+	*M.* sp. NBC_00419	SAMN30553070	GCF_036023875.1	
	+	+	+	*M.* sp. DL592	SAMN13155068	GCF_011694515.1	
	+	+	+	*M. virginiense*	SAMN20254270	GCF_022374935.2	
			+	*M. virginiense*	SAMN08580349	GCF_002967035.1	
			+	*M. sp. UM_NZ2*	SAMN03068182	GCF_000972935.1	
			+	*M. heraklionense*	SAMN04634209	GCF_001666995.1	
			+	*M. heraklionense*	SAMN04634164	GCF_001673105.1	
	+	+	+	*M. heraklionense*	SAMN04634187	GCF_001673575.1	*M.* sp. 1211594.5
	+	+	+	*M.* sp. MU0102	SAMEA114267547	GCF_963378105.1	
	+	+	+	*M. heraklionense*	SAMN29453539	GCA_024758765.1	*M.* sp. SVM_VP21
	+	+	+	*M. mephinesia*	SAMEA4979882	GCF_900604305.1	
			+	*M.* sp. UM_WGJ	SAMN02471858	GCF_000455125.1	
			+	*M.* sp. UM_Kg27	SAMN03068181	GCF_000972905.1	
	+	+	+	*M. heraklionense*	SAMN20254261	GCF_019645815.1	
			+	*M. heraklionense*	SAMN03734870	GCF_001021505.1	
	+	+	+	*M. icosiumassiliensis*	SAMEA3913389	GCF_900078685.2	
	+	+	+	*M. nonchromogenicum*	SAMN04216939	GCF_002101775.1	
			+	*M. nonchromogenicum*	SAMN15569017	GCA_025821265.1	
			+	*M. heraklionense*	SAMN04634253	GCF_001672655.1	
		+	+	*M. heraklionense*	SAMN04634273	GCF_001953995.1	
	+	+	+	*M. minnesotense*	SAMN06064248	GCF_002086405.1	
			+	*M. minnesotense*	SAMD00153210	GCF_010731755.1	
			+	*M. minnesotense*	SAMN15569008	GCF_025821425.1	
M. terrae	+	+	+	*M. arupense*	SAMN06064230	GCF_002086515.1	*M. arupense*
			+	*M. arupense*	SAMN03457165	GCF_000970885.2	*M. arupense*
			+	*M.* sp. UM_Kg17	SAMN03068180	GCF_000972915.1	*M. arupense*
			+	*M. arupense*	SAMN12697711	GCF_008373105.1	*M. arupense*
			+	*M. arupense*	SAMN15568945	GCF_025822685.1	*M. arupense*
	+	+	+	*M. engbaekii*	SAMN04216923	GCF_002101585.1	
	+	+	+	*M. hiberniae*	SAMN04216930	GCF_002101655.1	
			+	*M. hiberniae*	SAMD00153186	GCF_010729485.1	
			+	*M. hiberniae*	SAMN15569001	GCF_025821605.1	
		+	+	*M.* sp. UM_Kg1	SAMN03068179	GCF_000972925.1	
	+	+	+	*M. longobardum*	SAMN04216937	GCF_002102265.1	
			+	*M. longobardum*	SAMN15568922	GCF_025823135.1	
	+	+	+	*M.* sp. MU0083	SAMEA114267545	GCF_963378075.1	
	+	+	+	*M.* sp. MYC340	SAMN20062778	GCF_020172685.1	
	+	+	+	*M.* sp. MYC098	SAMN20959234	GCF_020181615.1	
	+	+	+	*M.* sp. MYC101	SAMN20959235	GCF_020181635.1	*M.* sp. MYC101
			+	*M.* sp. MYC123	SAMN20062777	GCF_020172665.1	*M.* sp. MYC101
		+	+	*M.* sp. MYC017	SAMN20959233	GCF_020181595.1	*M.* sp. MYC101
	+	+	+	*M. senuensis*	SAMN09476217	GCF_003284925.1	*M.* sp. GF74
		+	+	*M.* sp. GF74	SAMN42688601	GCF_041287085.1	*M.* sp. GF74
	+	+	+	*M. algericum*	SAMN06064225	GCF_002086455.1	*M. algericum*
			+	*M. algericum*	SAMD00153221	GCF_010723515.1	*M. algericum*

		+	+	*M. sinensis*	SAMN02603865	GCF_000214155.1	*M. algericum*
			+	*M. novum*	SAMD00153168	GCF_010726505.1	*M. algericum*
M. terrae	+	+	+	*M. senuense*	SAMD00153200	GCF_010723225.1	*M. senuensis*
			+	*M. senuense*	SAMN04216949	GCF_002101885.1	*M. senuensis*
	+	+	+	*M.* sp. CSURQ5927	SAMN32979486	GCA_028728255.1	*M. polyniensis*
			+	*M. sinensis*	SAMN04692039	GCF_001667395.1	*M. polyniensis*
			+	*M. sinensis*	SAMN04692035	GCF_001667375.1	*M. polyniensis*
		+	+	*M. sinensis*	SAMN04634186	GCF_001673565.1	*M. polyniensis*
	+	+	+	*M. sinensis*	SAMN04634248	GCF_001672735.1	*M.* sp. E1876
			+	*M. sinensis*	SAMN04634232	GCF_001667945.1	*M.* sp. E1876
			+	*M. terrae*	SAMN10448711	GCF_003933725.1	*M.* sp. E1876
	+	+	+	*M. terrae*	SAMEA4434518	GCF_900187145.1	
			+	*M. terrae*	SAMN04216954	GCF_002101955.1	
			+	*M. terrae*	SAMD00153172	GCF_010727125.1	
			+	*M. terrae*	SAMN15568981	GCF_025822005.1	
	+	+	+	*M. kumamotonense*	SAMN06064243	GCF_002086285.1	
			+	*M. kumamotonense*	SAMN13905685	GCF_010093495.1	
			+	*M. kumamotonense*	SAMN03793173	GCF_001679965.1	
			+	*M. avium subsp. avium Env 77*	SAMN02472016	GCF_000240505.1	
	+	+	+	*M. acidiphilus*	SAMN19113909	GCF_018455725.1	
M. triviale	+	+	+	*M. triviale*	SAMN04216956	GCF_002102395.1	
			+	*M. triviale*	SAMN15568993	GCF_025821865.1	
	+	+	+	*M.* sp. 1274756.6	SAMN04634181	GCF_001668625.1	*M.* sp. 1274756.6
	+	+	+	*M. koreense*	SAMD00153215	GCF_010731835.1	
			+	*M. koreense*	SAMN06651658	GCF_002104795.1	
			+	*M. koreense*	SAMN15568946	GCF_025822715.1	
			+	*M. triviale*	SAMN05729963	GCF_001722415.1	
	+	+	+	*M. parakoreense*	SAMN20254257	GCF_022370835.2	
			+	*M. parakoreense*	SAMN15568938	GCF_025822825.1	
M. talmoniae	+	+	+	*Mycobacterium sp.* pUA109	SAMN42692129	GCF_041287095.1	*M. moscowiense*
	+	+	+	*M. eburneum*	SAMN11101234	GCF_004354905.1	*M. talmoniae*
			+	*M. talmoniae*	SAMN08357531	GCF_002967005.1	*M. talmoniae*
			+	*M. talmoniae*	SAMN05909063	GCF_001854525.1	*M. talmoniae*
M. celatum	+	+	+	*M. branderi*	SAMN06064235	GCF_002086575.1	
			+	*M. branderi*	SAMD00153181	GCF_010728725.1	
			+	*M. branderi*	SAMN15568957	GCF_025822465.1	
	+	+	+	*M. kyorinense*	SAMD00019087	GCF_000759695.1	
			+	*M. kyorinense*	SAMN04216935	GCF_002101735.1	
			+	*M. kyorinense*	SAMN04196478	GCF_001439515.1	
	+	+	+	*M. celatum*	SAMN04216916	GCF_002101595.1	
			+	*M. celatum*	SAMD00025827	GCF_000974705.1	
			+	*M. celatum*	SAMN07733418	GCF_002742165.1	
	+	+	+	*M. sp. MFM001*	SAMD00098221	GCF_003402475.1	
	+	+	+	*M. kyorinense*	SAMN04634245	GCF_001672775.1	*M.* sp. E861
	+	+	+	*M. shimoidei*	SAMN04216951	GCF_002101905.1	
			+	*M. shimoidei*	SAMN15568941	GCF_025822885.1	

			+	*M. shimoidei*	SAMN05729965	GCF_001722445.1	
			+	*M. shimoidei*	SAMEA4681384	GCF_900417275.1	
	+	+	+	*M. fragae*	SAMN04216927	GCF_002102185.1	
			+	*M. fragae*	SAMN04101982	GCF_020622285.1	
	+	+	+	*M. methanotrophicum*	SAMEA8898435	GCF_910591625.1	
			+	*M. methanotrophicum*	SAMN28196450	GCF_023515995.1	
M. celatum	+	+	+	*M. cookii*	SAMD00153177	GCF_010727945.1	
			+	*M. cookii*	SAMN15568939	GCF_025822925.1	
	+	+	+	*M. wuenschmannii*	SAMN33244890	GCF_030252325.1	
	+	+	+	*M. sp. 1245111.1*	SAMN04634196	GCF_001673405.1	
	+	+	+	*M. paraterrae*	SAMN20254271	GCF_022430545.2	
	+	+	+	*M. botniense*	SAMD00153205	GCF_010723305.1	
	+	+	+	*M. sp. SM1*	SAMN18118619	GCF_018361265.1	
	+	+	+	*M. noviomagense*	SAMN06064252	GCF_002086415.1	
			+	*M. noviomagense*	SAMD00153203	GCF_010731635.1	
			+	*M. noviomagense*	SAMN15569016	GCF_025821305.1	
	+	+	+	*M. xenopi*	SAMEA101170168	GCF_900453395.1	
			+	*M. xenopi*	SAMN04216958	GCF_002102015.1	
			+	*M. xenopi*	SAMD00197529	GCF_009936235.1	
			+	*M. xenopi*	SAMN02641614	GCA_000523695.1	
M. xenopi			+	*M. xenopi*	SAMN02641613	GCA_000523715.1	
			+	*M. xenopi*	SAMN02470128	GCF_000257745.1	
			+	*M. xenopi*	SAMN32603694	GCF_027854175.1	
			+	*M. xenopi*	SAMN32603692	GCF_027854195.1	
			+	*M. xenopi*	SAMN32603693	GCF_027854205.1	
	+	+	+	*M. heckeshornense*	SAMD00265704	GCF_016592155.1	
			+	*M. heckeshornense*	SAMN15568980	GCF_025822025.1	
			+	*M. heckeshornense*	SAMN03793170	GCF_001077755.1	
			+	*M. heckeshornense*	SAMN05990901	GCF_001881585.2	
			+	*M. heckeshornense*	SAMD00261041	GCF_016861545.1	
	+	+	+	*M. paragordonae*	SAMN08222636	GCF_003614435.1	
			+	*M. paragordonae*	SAMD00153214	GCF_010723415.1	
		+	+	*M. gordonae*	SAMN05202166	GCF_001673255.1	
		+	+	*M. paragordonae*	SAMN13318592	GCF_009733755.1	
			+	*M. gordonae*	SAMN04634194	GCF_001673475.1	
		+	+	*M. paragordonae*	SAMN10799734	GCF_004359035.1	
			+	*M. paragordonae*	SAMN10799737	GCF_004359015.1	
			+	*M. paragordonae*	SAMN10799738	GCF_004359025.1	
M. gordonae	+	+	+	*M. gordonae*	SAMN04123344	GCF_001417955.1	*M.* sp. CTRI_14-8773
		+	+	*M.* sp. Z3061	SAMN37256842	GCF_031583025.1	*M.* sp. CTRI_14-8773
	+	+	+	*M.* sp. TY814	SAMN35524008	GCF_030755175.1	*M.* sp. TY814
	+	+	+	*M. gordonae*	SAMN18021193	GCF_017086405.1	*M.* sp. X7091
	+	+	+	*M. gordonae*	SAMN04216929	GCF_002101675.1	
			+	*M. gordonae*	SAMN05202167	GCF_001675225.1	
			+	*M. gordonae*	SAMN05730078	GCF_001722405.1	
			+	*M. gordonae*	SAMN19689020	GCF_024451565.1	
	+	+	+	*M. asiaticum*	SAMN04634167	GCF_001673365.1	*1165133.8*

M. gordonae	+	+	+	*M. asiaticum*	SAMN04634169	GCF_001673315.1	*1245139.5*
	+	+	+	*M. asiaticum*	SAMEA3138999	GCF_000613245.1	
			+	*M. asiaticum*	SAMN06064231	GCF_002086545.1	
			+	*M. asiaticum*	SAMN04634178	GCF_001669345.1	
			+	*M. asiaticum*	SAMN04634199	GCF_001672925.1	
			+	*M. asiaticum*	SAMN05202163	GCF_001673165.1	
	+	+	+	*M. asiaticum*	SAMN04634183	GCF_001673635.1	*M.* sp. 1275241.6
			+	*M. asiaticum*	SAMN04634168	GCF_001673345.1	*M.* sp. 1275241.6
	+	+	+	*M. asiaticum*	SAMN05202170	GCF_001668675.1	*M.* sp. 1081914.2
	+	+	+	*M. vicinigordonae*	SAMN15540336	GCF_013466425.1	
	+	+	+	*M. kiyosense*	SAMD00407201	GCF_021654635.1	
			+	*M.* sp. 20KCMC460	SAMD00432540	GCF_022179425.1	
			+	*M. kiyosense*	SAMD00505758	GCF_026011815.1	
			+	*M. kiyosense*	SAMD00505759	GCF_026011835.1	
			+	*M. kiyosense*	SAMD00505760	GCF_026011855.1	
			+	*M. kiyosense*	SAMD00505761	GCF_026011875.1	
	+	+	+	*M. intermedium*	SAMN06064242	GCF_002086275.1	
			+	*M. intermedium*	SAMN15568978	GCF_025822065.1	
			+	*M. intermedium*	SAMN05729961	GCF_001722345.1	
			+	*M. intermedium*	SAMN05729955	GCF_002024965.1	
	+	+	+	*M. bourgelatii*	SAMD00153222	GCF_010723575.1	
			+	*M. bourgelatii*	SAMN15568983	GCF_025821955.1	
	+	+	+	*M. kubicae*	SAMN16828374	GCF_015689175.1	
			+	*M. kubicae*	SAMD00153187	GCF_010723135.1	
			+	*M. kubicae*	SAMN15568989	GCF_025821825.1	
			+	*M. kubicae*	SAMN12910285	GCF_014263315.1	
			+	*M. kubicae*	SAMN04634263	GCF_001667525.1	
			+	*M. kubicae*	SAMN04634195	GCF_001673455.1	
			+	*M. kubicae*	SAMN04216934	GCF_002101745.1	
			+	*M. kubicae*	SAMN12910286	GCF_014263335.1	
	+	+	+	*M.* sp. 1423905.2	SAMN05202164	GCF_001673155.1	*M.* sp. 1423905.2
M. szulgai	+	+	+	*M. riyadhense*	SAMN04216947	GCF_002101845.1	
			+	*M. riyadhense*	SAMN15568995	GCF_025821735.1	
			+	*M. riyadhense*	SAMEA7003854	GCF_905219535.1	
			+	*M. riyadhense*	SAMN12495011	GCF_016864455.1	
			+	*M. riyadhense*	SAMEA7003858	GCF_905219475.1	
			+	*M. riyadhense*	SAMEA7003850	GCF_905219485.1	
	+	+	+	*M. simulans*	SAMEA104335938	GCF_900232995.1	
		+	+	*M. simulans*	SAMEA104346610	GCF_900232515.1	
	+	+	+	*M. szulgai*	SAMN04216953	GCF_002116635.1	
			+	*M. szulgai*	SAMN15569012	GCF_025821385.1	
	+	+	+	*M. angelicum*	SAMN06064227	GCF_002086155.1	
			+	*M. angelicum*	SAMN15568962	GCF_025822405.1	
M. tuberculosis	+	+	+	*M. tuberculosis*	SAMEA3138326	GCF_000195955.2	
			+	*M. tuberculosis*	SAMN02470820	GCF_000353205.1	
			+	*M. tuberculosis*	SAMN04942516	GCF_001652545.1	
			+	*M. tuberculosis*	SAMN11890852	GCF_006385035.1	

M. tuberculosis			+	*M. tuberculosis*	SAMN16564840	GCF_015265495.1	
	+	+	+	*M. decipiens*	SAMN06651657	GCF_002104675.1	
	+	+	+	*M. spongiae*	SAMN12776813	GCF_018278905.1	
	+	+	+	*M. shinjukuense*	SAMN06064260	GCF_002086755.1	
			+	*M. shinjukuense*	SAMD00153190	GCF_010730055.1	
			+	*M. shinjukuense*	SAMN15568966	GCF_025822285.1	
	+	+	+	*M. lacus*	SAMD00153198	GCF_010731535.1	
			+	*M. lacus*	SAMN04216936	GCF_002102215.1	
			+	*M. lacus*	SAMN15568998	GCF_025821645.1	
M. ulcerans	+	+	+	*M. marinum*	SAMN07792364	GCF_003391395.1	*M. marinum*
			+	*M. marinum*	SAMN14832045	GCF_016745295.1	*M. marinum*
			+	*M. liflandii*	SAMN07566685	GCA_022354805.1	*M. marinum*
			+	*M. marinum*	SAMN02604327	GCF_000018345.1	*M. marinum*
			+	*M. liflandii*	SAMN02603618	GCF_000026445.2	*M. marinum*
			+	*M. marinum*	SAMN02469865	GCF_000419315.1	*M. marinum*
			+	*M. marinum*	SAMEA3139029	GCF_000723425.2	*M. marinum*
			+	*M. marinum*	SAMN08394918	GCF_002911505.1	*M. marinum*
			+	*M. marinum*	SAMN08394919	GCF_002911515.1	*M. marinum*
		+	+	*M. shottsii*	SAMD00153180	GCF_010728525.1	*M. marinum*
	+	+	+	*M. ulcerans*	SAMN20254269	GCA_022374915.2	
			+	*M. ulcerans*	SAMN02641612	GCA_000524035.1	
	+	+	+	*M. basiliense*	SAMEA104628733	GCF_900292015.1	
	+	+	+	*M. simiae*	SAMN12672811	GCF_008370645.1	*M.* sp. JAL-560-SIM
M. kansasii	+	+	+	*M. attenuatum*	SAMEA4938330	GCF_900566085.1	
			+	*M. attenuatum*	SAMEA4938328	GCF_900566065.1	
			+	*M. kansasii*	SAMN06215600	GCA_002086865.1	
			+	*M. attenuatum*	SAMEA4938329	GCF_900566105.1	
	+	+	+	*M. gastri*	SAMN04216928	GCF_002102175.1	
			+	*M. gastri*	SAMN02597426	GCF_000521195.1	
	+	+	+	*M. ostraviense*	SAMN07196566	GCF_002705925.1	
			+	*M. ostraviense*	SAMN23242882	GCF_021183725.1	
			+	*M. ostraviense*	SAMN04599333	GCF_001632895.1	
	+	+	+	*M. pseudokansasii*	SAMEA4938324	GCF_900566075.1	
			+	*M. kansasii*	SAMN02641607	GCF_000524055.1	
			+	*M. kansasii*	SAMN04599332	GCF_001632915.1	
			+	*M. kansasii*	SAMN07197156	GCF_002705885.1	
			+	*M. kansasii*	SAMN07197158	GCF_002705935.1	
			+	*M. pseudokansasii*	SAMEA4938326	GCF_900566025.1	
			+	*M. pseudokansasii*	SAMEA4938325	GCF_900566045.1	
	+	+	+	*M. kansasii*	SAMN02603596	GCF_000157895.3	
			+	*M. kansasii*	SAMD00233320	GCA_014701265.1	
			+	*M. kansasii*	SAMN02641606	GCF_000523615.1	
			+	*M. kansasii*	SAMN02641608	GCF_000524095.1	
			+	*M. kansasii*	SAMN02798027	GCF_000715375.1	
			+	*M. kansasii*	SAMN04599336	GCF_001632965.1	
			+	*M. kansasii*	SAMN06339043	GCF_002086895.1	
			+	*M. kansasii*	SAMN08397726	GCF_002920735.1	

			+	*M. kansasii*	SAMEA4938320	GCF_900565995.1	
	+	+	+	*M. persicum*	SAMN06064255	GCF_002086675.1	
			+	*M. persicum*	SAMEA6620858	GCF_902825395.1	
			+	*M. persicum MK4*	SAMEA4938323	GCF_900566035.1	
			+	*M. persicum MK42*	SAMEA4938322	GCF_900566015.1	
			+	*M. persicum MK15*	SAMEA4938321	GCF_900566005.1	
M. kansasii			+	*M. persicum*	SAMN07193079	GCF_002705895.1	
	+	+	+	*M. innocens*	SAMEA4938327	GCF_900566055.1	
			+	*M. kansasii*	SAMN04599334	GCF_001632885.1	
			+	*M. kansasii*	SAMN04599330	GCF_001632905.1	
			+	*M. kansasii*	SAMN07197157	GCF_002705965.1	
	+	+	+	*M. lepromatosis*	SAMN03481272	GCF_000975265.2	
			+	*M. lepromatosis*	SAMN02900753	GCF_000966355.1	
	+	+	+	*M. leprae*	SAMEA1705921	GCF_000195855.1	
			+	*M. leprae*	SAMEA2271916	GCF_000026685.1	
			+	*M. leprae*	SAMN04578110	GCF_001648835.1	
			+	*M. leprae*	SAMN05172957	GCF_001653495.1	
			+	*M. leprae*	SAMN09104579	GCF_003253775.1	
M. leprae			+	*M. leprae*	SAMD00011899	GCF_003584725.1	
	+	+	+	*M. uberis*	SAMN08894308	GCF_003408705.1	
	+	+	+	*M. haemophilum*	SAMN01918491	GCF_000340435.2	
			+	*M. haemophilum*	SAMN03734844	GCF_001021405.1	
			+	*M. haemophilum*	SAMN03734842	GCF_001021415.1	
			+	*M. haemophilum*	SAMN03734843	GCF_001021435.1	
			+	*M. haemophilum*	SAMN03734845	GCF_001021485.1	
			+	*M. haemophilum*	SAMN15568927	GCF_025823035.1	
	+	+	+	*M.* sp. 1164966.3	SAMN05202168	GCF_001665395.1	*M.* sp. 1164966.3
	+	+	+	*M.* sp. 852002-51163_SCH5372311	SAMN04696152	GCF_001667115.1	*M.* sp. SCH5372311
	+	+	+	*M. conspicuum*	SAMD00153191	GCF_010730195.1	
			+	*M. conspicuum*	SAMN04216921	GCF_002102095.1	
			+	*M. conspicuum*	SAMN15568991	GCF_025821765.1	
	+	+	+	*M. ahvazicum*	SAMEA103958175	GCF_900176255.2	
	+	+	+	*M.* sp. 050272	SAMN41075872	GCF_039023525.1	*M.* sp. 050272
	+	+	+	*M.* sp. pR1184	SAMN42692128	GCF_041287235.1	*M. peterburgiense*
	+	+	+	*M.* sp. 050128	SAMN38286611	GCF_036409155.1	*M.* sp. 050128
	+	+	+	*M. lentiflavum*	SAMN20254258	GCF_022374895.2	
			+	*M. lentiflavum*	SAMEA3305053	GCF_001373395.1	
	+	+	+	*M.* sp. Aquia_213	SAMN31424470	GCF_026625985.1	*M.* sp. Aquia_213
M. simiae	+	+	+	*M. stomatepiae*	SAMD00153208	GCF_010731715.1	
			+	*M. stomatepiae*	SAMN15568992	GCF_025821785.1	
	+	+	+	*M. florentinum*	SAMD00153192	GCF_010730355.1	
			+	*M. florentinum*	SAMN04216926	GCF_002101635.1	
			+	*M. florentinum*	SAMN15568915	GCF_025823305.1	
	+	+	+	*M. triplex*	SAMN04216955	GCF_002102415.1	
			+	*M. triplex*	SAMEA3139001	GCF_000689255.1	
	+	+	+	*M. genavense*	SAMN02585006	GCF_000526915.1	

	+	+	+	*M.* sp. Aquia_216	SAMN31424471	GCF_026723865.1	*M.* sp. Aquia_216
	+	+	+	*M. montefiorense*	SAMN15568917	GCF_025823255.1	
			+	*M. montefiorense*	SAMD00467631	GCF_026012615.1	
		+	+	*M. montefiorense*	SAMD00114206	GCF_003112775.1	
			+	*M. montefiorense*	SAMD00452371	GCF_024341945.1	
			+	*M. montefiorense*	SAMD00452372	GCF_024341965.1	
			+	*M. montefiorense*	SAMD00452373	GCF_024341985.1	
			+	*M. montefiorense*	SAMD00452374	GCF_024342005.1	
			+	*M. montefiorense*	SAMD00452375	GCF_024342025.1	
			+	*M. montefiorense*	SAMD00452376	GCF_024342045.1	
			+	*M. montefiorense*	SAMD00452377	GCF_024342065.1	
			+	*M. montefiorense*	SAMD00452378	GCF_024342085.1	
	+	+	+	*M.* sp. 1100029.7	SAMN04634185	GCF_001665235.1	*M.* sp. 1100029.7
M. simiae	+	+	+	*M. tilburgii*	SAMEA5775539	GCF_902168065.1	
	+	+	+	*M. simiae*	SAMD00153175	GCF_010727605.1	
			+	*M. simiae*	SAMEA2272207	GCF_000455305.1	
		+	+	*M. simiae*	SAMN06564598	GCF_002093075.1	
	+	+	+	*M. sherrisii*	SAMN04216950	GCF_002102355.1	
			+	*M. sherrisii*	SAMN15568979	GCF_025822045.1	
			+	*M. sherrisii*	SAMN05729962	GCF_001722325.1	
	+	+	+	*M. numidiamassiliense*	SAMEA54001918	GCF_900157365.1	
	+	+	+	*M. shigaense*	SAMD00081394	GCF_002356315.1	
			+	*M. shigaense*	SAMN06448869	GCF_002983495.1	
			+	*M. shigaense*	SAMN07510626	GCF_003515645.1	
	+	+	+	*M. rhizamassiliense*	SAMEA54078418	GCF_900157375.1	
	+	+	+	*M. terramassiliense*	SAMEA54120418	GCF_900157385.1	
	+	+	+	*M. interjectum*	SAMN04216932	GCF_002102225.1	*M. interjectum.I*
	+	+	+	*M. interjectum*	SAMN15569009	GCF_025821415.1	*M. interjectum.II*
			+	*M. interjectum*	SAMEA3913390	GCF_900078675.2	*M. interjectum.II*
	+	+	+	*M. sp. E3198*	SAMN04634211	GCF_001667015.1	
	+	+	+	*M. sp. 852002-50816_SCH5313054-b*	SAMN04696148	GCF_001667185.1	*M.* sp. SCH5313054
	+	+	+	*M. paraense*	SAMN04216942	GCF_002101815.1	
			+	*M. paraense*	SAMN04216943	GCF_002101825.1	
			+	*M. paraense*	SAMN04216944	GCF_002102295.1	
			+	*M. paraense*	SAMN04216941	GCF_002102305.1	
M. interjectum	+	+	+	*M.* sp. E787	SAMN04634243	GCF_001668725.1	*M.* sp. E787
			+	*M.* sp. E2699	SAMN04634234	GCF_001666745.1	*M.* sp. E787
	+	+	+	*M.* sp. 1554424.7	SAMN04634179	GCF_001665295.1	
	+	+	+	*M.* sp. 1081908.1	SAMN04634193	GCF_001673535.1	
	+	+	+	*M.* sp. 1245805.9	SAMN05202169	GCF_001672915.1	
	+	+	+	*M.* sp. IEC1808	SAMN04216952	GCF_002101935.1	*M.* sp. IEC1808
		+	+	*M.* sp. UM_CSW	SAMN04287657	GCF_000455205.1	*M.* sp. IEC1808
		+	+	*M.* sp. E2327	SAMN04634250	GCF_001672675.1	*M.* sp. IEC1808
	+	+	+	*M.* sp. E796	SAMN04634244	GCF_001672815.1	
	+	+	+	*M. alsense*	SAMN06064226	GCF_002086635.1	
			+	*M. alsense*	SAMN15568921	GCF_025823205.1	

M. interjectum		+	+	*M. alsense*	SAMN04634198	GCF_001672935.1	
			+	*M. alsense*	SAMN04634210	GCF_001666815.1	
	+	+	+	*M. sp. 852002-51057_SCH5723018*	SAMN04692007	GCF_001667035.1	*M.* sp. SCH5723018
	+	+	+	*M. palustre*	SAMN04216940	GCF_002101785.1	
			+	*M. palustre*	SAMN15569007	GCF_025821485.1	
	+	+	+	*M. heidelbergense*	SAMN06064240	GCF_002086215.1	
			+	*M. heidelbergense*	SAMD00153194	GCF_010730745.1	
			+	*M. heidelbergense*	SAMN15568984	GCF_025821945.1	
	+	+	+	*M. malmoense*	SAMN20254263	GCF_019645855.1	
			+	*M. malmoense*	SAMN15569027	GCF_022558505.1	
			+	*M. malmoense*	SAMN06064244	GCF_002086305.1	
			+	*M. malmoense*	SAMN05969904	GCF_001870845.1	
	+	+	+	*M. sp. 663a-19*	SAMN31248680	GCF_035616495.1	
	+	+	+	*M. sp. IS-836*	SAMN04634285	GCF_001954275.1	*M.* sp. IS-836
			+	*M. sp. SP-6446*	SAMN04634274	GCF_001954045.1	*M.* sp. IS-836
	+	+	+	*M. sp. IS-2888*	SAMN04634283	GCF_001954215.1	*M.* sp. IS-2888
			+	*M. sp. IS-1264*	SAMN04634282	GCF_001954195.1	*M.* sp. IS-2888
M. bohemicum	+	+	+	*M. saskatchewanense*	SAMN04216948	GCF_002101875.1	
			+	*M. saskatchewanense*	SAMD00153183	GCF_010729105.1	
			+	*M. saskatchewanense*	SAMN15569014	GCF_025821405.1	
	+	+	+	*M.* sp. 050134	SAMN38286612	GCF_036409085.1	
	+	+	+	*M. cambodiensis*	SAMEA6157417	GCF_902652685.1	
	+	+	+	*M. bohemicum*	SAMN04216914	GCF_002102025.1	
			+	*M. bohemicum*	SAMEA3305052	GCF_001053185.1	
			+	*M. bohemicum*	SAMN15568985	GCF_025821915.1	
	+	+	+	*M. helveticum*	SAMN12187686	GCF_007714205.1	
			▪	*M. helveticum*	SAMN12284734	GCF_007714185.1	
	+	+	+	*M.* sp. HUMS_12744610	SAMN42741947	GCF_041206865.1	
	+	+	+	*M. parmense*	SAMD00153193	GCF_010730575.1	
			+	*M. parmense*	SAMN04216945	GCF_002102335.1	
			+	*M. parmense*	SAMN15568931	GCF_025822965.1	
M. scrofulaceum	+	+	+	*M. sp. 852014-52450_SCH5900713*	SAMN04692044	GCF_001667075.1	*M.* sp. SCH5900713
			+	*M. sp. 852002-51613_SCH5001154*	SAMN04691946	GCF_001667425.1	*M.* sp. SCH5900713
			+	*M. sp. 852002-53434_SCH5985345*	SAMN04691965	GCF_001667155.1	*M.* sp. SCH5900713
	+	+	+	*M. paraffinicum*	SAMN15568937	GCF_025822865.1	
		+	+	*M.* sp. 852002-30065_SCH5024008	SAMN04696142	GCF_001665645.1	
			+	*M. sp. 852014-50255_SCH5639931*	SAMN04691973	GCF_001665605.1	
	+	+	+	*M.* sp. E1386	SAMN04634247	GCF_001672745.1	*M.* sp. E1386
			+	*M.* sp. E3251	SAMN04634235	GCF_001666835.1	*M.* sp. E1386
			+	*M.* sp. E2238	SAMN04634249	GCF_001672665.1	*M.* sp. E1386
	+	+	+	*M.* sp. E2733	SAMN04634216	GCF_001667585.1	*M.* sp. E2733
	+	+	+	*M. nebraskense*	SAMN15568988	GCF_025821845.1	
			+	*M. nebraskense*	SAMN04216938	GCF_002102255.1	

			+	*M. nebraskense*	SAMN03734848	GCF_001021495.1	
			+	*M. nebraskense*	SAMN03458449	GCF_000970875.1	
M. scrofulaceum	+	+	+	*M. scrofulaceum*	SAMN06064259	GCF_002086735.1	
			+	*M. scrofulaceum*	SAMN15569015	GCA_025821325.1	
	+	+	+	*M. parascrofulaceum*	SAMN00189881	GCF_000164135.1	
			+	*M. malmoense*	SAMN05202171	GCF_001686725.1	
		+	+	*M. malmoense*	SAMN04634213	GCF_001686825.1	
			+	*M. malmoense*	SAMN04634212	GCF_001686665.1	
			+	*M. malmoense*	SAMN04634214	GCF_001686735.1	
			+	*M. malmoense*	SAMN04634215	GCF_001686745.1	
	+	+	+	*M. paraseoulense*	SAMN06064254	GCF_002086475.1	
			+	*M. paraseoulense*	SAMD00153204	GCF_010731655.1	
	+	+	+	*M. seoulense*	SAMD00153201	GCF_010731595.1	
			+	*M. seoulense*	SAMN15568913	GCF_025823355.1	
	+	+	+	*M. europaeum*	SAMEA3305049	GCF_001373515.1	
			+	*M. europaeum*	SAMN04216924	GCF_002102155.1	
			+	*M. europaeum*	SAMN38872439	GCF_034555895.1	
	+	+	+	*M.* sp. E342	SAMN04634230	GCF_001667665.1	*M.* sp. *E342*
		+	+	*M.* sp. 4858	SAMEA104417325	GCF_900240975.1	*M.* sp. *E342*
			+	*M.* sp. E3247	SAMN05202173	GCF_001667785.1	*M.* sp. *E342*
			+	*M.* sp. E2497	SAMN04634251	GCF_001668695.1	*M.* sp. *E342*
	+	+	+	*M.* sp. SMC-2	SAMN20167550	GCF_025263485.1	*M.* sp. *SMC-2*
	+	+	+	*M.* sp. E2989	SAMN04634218	GCF_001667595.1	*M.* sp. *E2989*
			+	*M.* sp. UM_11	SAMN03458310	GCF_001414075.1	*M.* sp. *E2989*
			+	*M.* sp. UM_3	SAMN03458309	GCF_001414095.1	*M.* sp. *E2989*
			+	*M.* sp. E3339	SAMN04634240	GCF_001666915.1	*M.* sp. *E2989*
		+	+	*M.* sp. ACS4054	SAMN04634268	GCF_001667275.1	*M.* sp. *E2989*
	+	+	+	*M.* sp. E1747	SAMN04634233	GCF_001667775.1	*M.* sp. *E1747*
	+	+	+	*M.* sp. E183	SAMN04634229	GCF_001667735.1	*M.* sp. *E183*
			+	*M.* sp. E3305	SAMN04634239	GCF_001666785.1	*M.* sp. *E183*
			+	*M.* sp. E3298	SAMN05202174	GCF_001666895.1	*M.* sp. *E183*
			+	*M.* sp. E188	SAMN04634241	GCF_001666935.1	*M.* sp. *E183*
			+	*M.* sp. E735	SAMN04634242	GCF_001666955.1	*M.* sp. *E183*
			+	*M.* sp. E1715	SAMN05202172	GCF_001667625.1	*M.* sp. *E183*
	+	+	+	*M. scrofulaceum*	SAMN04634217	GCF_001672575.1	*M.* sp. *E2838*
			+	*M. scrofulaceum*	SAMN04634219	GCF_001667885.1	*M.* sp. *E2838*
	**+**	**+**	**+**	*M.* sp. HNNTM2301	SAMN43334327	GCF_041890355.1	
	+	+	+	*M. paraffinicum*	SAMN06034714	GCF_001907675.1	*M.* sp. *M11*
	+	+	+	*M. sp. E2462*	SAMN04634252	GCF_001672685.1	*E2462*
			+	*M. sp. E1214*	SAMN04634238	GCF_001666755.1	*E2462*
			+	*M. sp. E1319*	SAMN04634231	GCF_001667745.1	*E2462*
M. avium	+	+	+	*M. intracellulare subsp. intracellulare*	SAMN02603186	GCF_000277125.1	
			+	*M. intracellulare*	SAMN02603185	GCF_000276825.1	
			+	*M. intracellulare*	SAMN06651659	GCF_002104735.1	
			+	*M. intracellulare*	SAMD00153220	GCF_010731935.1	
			+	*M. intracellulare*	SAMD00258751	GCF_016587595.1	

			+	*M. intracellulare*	SAMD00258748	GCF_016587655.1	
		+	+	*M. intracellulare subsp. yongonense*	SAMN02603187	GCF_000418535.1	
			+	*M. sp. MOTT36Y*	SAMN02603851	GCF_000262165.1	
			+	*M. sp. H4Y*	SAMN02470905	GCF_000364405.1	
			+	*M. sp. TKK-01-0059*	SAMN02053792	GCF_000709305.1	
		+	+	*M. intracellulare subsp. chimaera*	SAMN06111444	GCF_001901205.1	
	+	+	+	*M. timonense*	SAMD00153223	GCF_010723675.1	*M. timonense*
		+	+	*M. marseillense*	SAMN06064246	GCF_002086345.1	*M. timonense*
			+	*M. marseillense*	SAMD00153206	GCF_010731675.1	*M. timonense*
			+	*M. marseillense*	SAMN15568923	GCF_025823125.1	*M. timonense*
			+	*M. marseillense*	SAMN04634159	GCF_001673235.1	*M. timonense*
			+	*M. marseillense*	SAMN07528776	GCF_002285715.1	*M. timonense*
			+	*M. marseillense*	SAMN11577515	GCF_007654935.1	*M. timonense*
			+	*M. marseillense*	SAMN16989000	GCF_020217515.1	*M. timonense*
	+	+	+	*M. sp. WUMAC-025*	SAMN16988985	GCF_020217805.1	*M.* sp. WUMAC-025
			+	*M. sp. WUMAC-067*	SAMN16989001	GCF_020217485.1	*M.* sp. WUMAC-025
	+	+	+	*M. sp. 852002-10029_SCH5224772*	SAMN04696135	GCF_001667315.1	*M.* sp. SCH5224772
	+	+	+	*M. avium*	SAMN13352280	GCF_009741445.1	
			+	*M. avium*	SAMN02470543	GCF_000174035.1	
			+	*M. avium subsp. paratuberculosis*	SAMN02604086	GCF_000007865.1	
			+	*M. sp. MAC_011194_8550*	SAMN02641624	GCF_000523635.1	
			+	*M. sp. MAC_080597_8934*	SAMN02641625	GCF_000523975.1	
M. avium			+	*M. avium*	SAMN15568982	GCF_025821985.1	
			+	*M. avium*	SAMN15568974	GCF_025822125.1	
			+	*M. avium*	SAMN06064261	GCF_002086775.1	
			+	*M. avium*	SAMN06064234	GCF_002086165.1	
		+	+	*M. lepraemurium*	SAMN06841017	GCF_002291465.1	
	+	+	+	*M. sp. 94-17*	SAMN31248620	GCF_035617725.1	
	+	+	+	*M. sp. 1465703. 0*	SAMN04634158	GCF_001672975.1	
	+	+	+	*M. sp. 852002-51971_SCH5477799-a*	SAMN04691956	GCF_001667145.1	*M.* sp. SCH5477799
			+	*M. sp. 1165178. 9*	SAMN04634190	GCF_001673555.1	*M.* sp. SCH5477799
	+	+	+	*M. sp. E2479*	SAMN04634224	GCF_001667925.1	
	+	+	+	*M. mantenii*	SAMN06064245	GCF_002086335.1	
			+	*M. mantenii*	SAMD00153211	GCF_010731775.1	
			+	*M. mantenii*	SAMN15568949	GCF_025822645.1	
		+	+	*M. mantenii*	SAMN04634225	GCF_001667995.1	
			+	*M. mantenii*	SAMN04634221	GCF_001667695.1	
			+	*M. mantenii*	SAMN04634220	GCF_001667835.1	
			+	*M. mantenii*	SAMN04634226	GCF_001667865.1	
	+	+	+	*M. sp. 1245499. 0*	SAMN04634184	GCF_001673615.1	*M.* sp. 1245499.0
			+	*M. sp. 1482292.6*	SAMN04634157	GCF_001672995.1	*M.* sp. 1245499.0
			+	*M. sp. 1245801.1*	SAMN04634155	GCF_001673055.1	*M.* sp. 1245499.0
			+	*M. sp. 1165196.3*	SAMN04634197	GCF_001673415.1	*M.* sp. 1245499.0
			+	*M. sp. 1245852.3*	SAMN05202165	GCF_001669335.1	*M.* sp. 1245499.0

		+	+	*M. colombiense*	SAMN09476220	GCF_003284975.1	*M.* sp. 1245499.0
	+	+	+	*M. colombiense*	SAMN09476219	GCF_003284935.1	*M.* sp. GF28
	+	+	+	*M. arosiense*	SAMN06064229	GCF_002086125.1	
M. avium	+	+	+	*M. sp. 852002-40037_SCH5390672*	SAMN04696143	GCF_001665875.1	*M.* sp. SCH5390672
	+	+	+	*M. colombiense*	SAMN06760893	GCF_002105755.1	
			+	*M. colombiense*	SAMN00622208	GCF_000222105.3	
		+	+	*M. colombiense*	SAMEA59602168	GCF_900161855.1	
			+	*M. sp. TKK-01-0051*	SAMN02053784	GCF_000661085.1	
			+	*M. colombiense*	SAMN27759972	GCF_023218095.1	
	+	+	+	*M. senriense*	SAMD00250051	GCF_019668465.1	
	+	+	+	*M. colombiense*	SAMN04634277	GCF_001954115.1	*M.* sp. IS-576
			+	*M. colombiense*	SAMN04634270	GCF_001953985.1	*M.* sp. IS-576
			+	*M. colombiense*	SAMN04634275	GCF_001954055.1	*M.* sp. IS-576
			+	*M. colombiense*	SAMN04634276	GCF_001954075.1	*M.* sp. IS-576
		+	+	*M. colombiense*	SAMN04634191	GCF_001673505.1	*M.* sp. IS-576
			+	*M. colombiense*	SAMN04634156	GCF_001673005.1	*M.* sp. IS-576
			+	*M. colombiense*	SAMN05202162	GCF_001673015.1	*M.* sp. IS-576
			+	*M. colombiense*	SAMN04634154	GCF_001673075.1	*M.* sp. IS-576
			+	*M. colombiense*	SAMN04634153	GCF_001673085.1	*M.* sp. IS-576
			+	*M. colombiense*	SAMN04634161	GCF_001673175.1	*M.* sp. IS-576
			+	*M. colombiense*	SAMN04634160	GCF_001673195.1	*M.* sp. IS-576
		+	+	*M. colombiense*	SAMN04634223	GCF_001667905.1	*M.* sp. IS-576
	+	+	+	*M. colombiense*	SAMN04691980	GCF_001665835.1	*M.* sp. SCH5396731
	+	+	+	*M. vulneris*	SAMN06651660	GCF_002104765.1	
	+	+	+	*M. colombiense*	SAMN04634246	GCF_001672755.1	*M.* sp. E1334

Table S2. List of 315 *Mycobacteriales* genomes and 4 genomes of *Microbacterium*.

Four reference *Mycobacteriales* genomes used as root for *Mycobacterium* tree reconstruction are shown in bold type.

Cluster	Family	Genus	Specie	Sample	Dir
Mycobacteriales	*Segniliparaceae*	*Segniliparus*	*Segniliparus rotundus*	SAMN02598516	GCF_000092825.1
Mycobacteriales	*Nocardiaceae*	*Hoyosella*	*Hoyosella subflava*	SAMN02603151	GCF_000214175.1
Mycobacteriales	*Nocardiaceae*	*Nocardia*	*Nocardia huaxiensis*	SAMN23376436	GCF_021044705.1
Mycobacteriales	*Nocardiaceae*	*Rhodococcus*	*Rhodococcus sp. WMMA185*	SAMN05560454	GCF_001767395.1
Mycobacteriales	*Nocardiaceae*	*Rhodococcus*	*Rhodococcus oxybenzonivorans*	SAMN07126182	GCF_003130705.1
Mycobacteriales	*Nocardiaceae*	*Rhodococcus*	*Rhodococcus opacus*	SAMN26492386	GCF_022985635.2
Mycobacteriales	*Nocardiaceae*	*Rhodococcus*	*Rhodococcus sp. WB9*	SAMN12234611	GCF_007558985.1
Mycobacteriales	*Nocardiaceae*	*Rhodococcus*	*Rhodococcus sp. 21391*	SAMN13510402	GCF_016772255.1
Mycobacteriales	*Nocardiaceae*	*Rhodococcus*	*Rhodococcus opacus*	SAMN12617337	GCF_019856255.1
Mycobacteriales	*Nocardiaceae*	*Rhodococcus*	*Rhodococcus jostii*	SAMN02604146	GCF_000014565.1
Mycobacteriales	*Nocardiaceae*	*Rhodococcus*	*Rhodococcus sp. JS3073*	SAMN32034740	GCF_026810085.1
Mycobacteriales	*Nocardiaceae*	*Rhodococcus*	*Rhodococcus sp. ABRD24*	SAMN10761912	GCF_004328705.1
Mycobacteriales	*Nocardiaceae*	*Rhodococcus*	*Rhodococcus sp. PAMC28707*	SAMN11370749	GCF_004795915.1
Mycobacteriales	*Nocardiaceae*	*Rhodococcus*	*Rhodococcus sp. 75*	SAMN31373510	GCF_026153295.1
Mycobacteriales	*Nocardiaceae*	*Rhodococcus*	*Rhodococcus sp. X156*	SAMN10651094	GCF_004006015.1
Mycobacteriales	*Nocardiaceae*	*Rhodococcus*	*Rhodococcus sp. P1Y*	SAMN10180271	GCF_003641205.1
Mycobacteriales	*Gordoniaceae*	*Gordonia*	*Gordonia hongkongensis*	SAMN27409696	GCF_023078355.1
Mycobacteriales	*Gordoniaceae*	*Gordonia*	*Gordonia sp. JH63*	SAMN13680426	GCF_009856645.1
Mycobacteriales	*Gordoniaceae*	*Gordonia*	*Gordonia sp. WA4-43*	SAMN21584138	GCF_020520305.1
Mycobacteriales	*Gordoniaceae*	*Gordonia*	*Gordonia terrae*	SAMN05417667	GCF_001698225.1
Mycobacteriales	*Gordoniaceae*	*Gordonia*	*Gordonia terrae*	SAMN27776997	GCF_023238525.1
Mycobacteriales	*Gordoniaceae*	*Gordonia*	*Gordonia alkanivorans*	SAMN26527893	GCF_022591595.1
Mycobacteriales	*Gordoniaceae*	*Gordonia*	*Gordonia sp. 135*	SAMN13326465	GCF_009720185.1
Mycobacteriales	*Gordoniaceae*	*Gordonia*	*Gordonia rubripertincta*	SAMN07356858	GCF_003568625.1
Mycobacteriales	*Gordoniaceae*	*Gordonia*	*Gordonia insulae*	SAMN10160079	GCF_003855095.1
Mycobacteriales	*Gordoniaceae*	*Gordonia*	*Gordonia polyisoprenivorans*	SAMN18477556	GCF_017654315.1
Mycobacteriales	*Gordoniaceae*	*Gordonia*	*Gordonia otitidis*	SAMN22365129	GCF_020735545.1
Mycobacteriales	*Gordoniaceae*	*Gordonia*	*Gordonia iterans*	SAMN08634400	GCF_002993285.1
Mycobacteriales	*Gordoniaceae*	*Gordonia*	*Gordonia sp. PDNC005*	SAMN17844877	GCF_016919385.1
Mycobacteriales	*Gordoniaceae*	*Gordonia*	*Gordonia zhaorongruii*	SAMN12318967	GCF_007559005.1
Mycobacteriales	*Gordoniaceae*	*Gordonia*	*Gordonia phthalatica*	SAMN03776117	GCF_001305675.1
Mycobacteriales	*Dietziaceae*	*Dietzia*	*Dietzia psychralcaliphila*	SAMN04880516	GCF_003096095.1
Mycobacteriales	*Dietziaceae*	*Dietzia*	*Dietzia kunjamensis*	SAMN29261762	GCF_024125715.1
Mycobacteriales	*Dietziaceae*	*Dietzia*	*Dietzia sp. B32*	SAMN25598616	GCF_024732245.1
Mycobacteriales	*Dietziaceae*	*Dietzia*	*Dietzia lutea*	SAMN04880518	GCF_003096075.1
Mycobacteriales	*Dietziaceae*	*Dietzia*	*Dietzia timorensis*	SAMN04219460	GCF_001659785.1
Mycobacteriales	*Lawsonellaceae*	*Lawsonella*	*Lawsonella clevelandensis*	SAMEA5065507	GCF_900610365.1
Mycobacteriales	*Lawsonellaceae*	*Lawsonella*	*Lawsonella clevelandensis*	SAMN02943398	GCF_001293125.1
Mycobacteriales	*Lawsonellaceae*	*Lawsonella*	*Lawsonella clevelandensis*	SAMN03943545	GCF_001281505.1
Mycobacteriales	*Corynebacteriaceae*	*Corynebacterium*	*Corynebacterium segmentosum*	SAMEA37377418	GCF_900637825.1
Mycobacteriales	*Corynebacteriaceae*	*Corynebacterium*	*Corynebacterium accolens*	SAMN13404507	GCF_023520795.1
Mycobacteriales	*Corynebacteriaceae*	*Corynebacterium*	*Corynebacterium macginleyi*	SAMN16357337	GCF_016889465.1
Mycobacteriales	*Corynebacteriaceae*	*Corynebacterium*	*Corynebacterium minutissimum*	SAMN16357161	GCF_016126915.1
Mycobacteriales	*Corynebacteriaceae*	*Corynebacterium*	*Corynebacterium camporealensis*	SAMN03092874	GCF_000766885.2
Mycobacteriales	*Corynebacteriaceae*	*Corynebacterium*	*Corynebacterium endometrii*	SAMN11357123	GCF_004795735.1
Mycobacteriales	*Corynebacteriaceae*	*Corynebacterium*	*Corynebacterium flavescens*	SAMN02996497	GCF_001941465.1
Mycobacteriales	*Corynebacteriaceae*	*Corynebacterium*	*Corynebacterium wankanglinii*	SAMN15954653	GCA_014490595.1
Mycobacteriales	*Corynebacteriaceae*	*Corynebacterium*	*Corynebacterium ureicelerivorans*	SAMN02953970	GCF_000747315.1
Mycobacteriales	*Corynebacteriaceae*	*Corynebacterium*	*Corynebacterium lujinxingii*	SAMN15951546	GCF_014490555.1
Mycobacteriales	*Corynebacteriaceae*	*Corynebacterium*	*Corynebacterium coyleae*	SAMN21218848	GCF_020097655.1
Mycobacteriales	*Corynebacteriaceae*	*Corynebacterium*	*Corynebacterium aquatimens*	SAMN17304831	GCA_019443945.1

Mycobacteriales	*Corynebacteriaceae*	*Corynebacterium*	*Corynebacterium timonense*	SAMN04488539	GCF_900105305.1
Mycobacteriales	*Corynebacteriaceae*	*Corynebacterium*	*Corynebacterium mycetoides*	SAMN04488535	GCF_900103625.1
Mycobacteriales	*Corynebacteriaceae*	*Corynebacterium*	*Corynebacterium glaucum*	SAMN03854643	GCF_002287505.1
Mycobacteriales	*Corynebacteriaceae*	*Corynebacterium*	*Corynebacterium qintianiae*	SAMN14086271	GCF_011038645.2
Mycobacteriales	*Corynebacteriaceae*	*Corynebacterium*	*Corynebacterium genitalium*	SAMN18647610	GCF_024453835.1
Mycobacteriales	*Corynebacteriaceae*	*Corynebacterium*	*Corynebacterium liangguodongii*	SAMN08388739	GCF_003070865.1
Mycobacteriales	*Corynebacteriaceae*	*Corynebacterium*	*Corynebacterium yudongzhengii*	SAMN08388720	GCF_003065405.1
Mycobacteriales	*Corynebacteriaceae*	*Corynebacterium*	*Corynebacterium comes*	SAMN13284570	GCF_009734405.1
Mycobacteriales	*Corynebacteriaceae*	*Corynebacterium*	*Corynebacterium marinum*	SAMN02800399	GCF_000835165.1
Mycobacteriales	*Corynebacteriaceae*	*Corynebacterium*	*Corynebacterium humireducens*	SAMN03283197	GCF_000819445.1
Mycobacteriales	*Corynebacteriaceae*	*Corynebacterium*	*Corynebacterium maris*	SAMN02603057	GCF_000442645.1
Mycobacteriales	*Corynebacteriaceae*	*Corynebacterium*	*Corynebacterium frankenforstense*	SAMN02991553	GCF_001941485.1
Mycobacteriales	*Corynebacteriaceae*	*Corynebacterium*	*Corynebacterium efficiens*	SAMD00061103	GCF_000011305.1
Mycobacteriales	*Corynebacteriaceae*	*Corynebacterium*	*Corynebacterium falsenii*	SAMN21218849	GCF_020097615.1
Mycobacteriales	*Corynebacteriaceae*	*Corynebacterium*	*Corynebacterium freneyi*	SAMN16357568	GCF_019047805.1
Mycobacteriales	*Corynebacteriaceae*	*Corynebacterium*	*Corynebacterium xerosis*	SAMN11056389	GCF_009730475.1
Mycobacteriales	*Corynebacteriaceae*	*Corynebacterium*	*Corynebacterium suranareeae*	SAMD00000552	GCF_002355155.1
Mycobacteriales	*Corynebacteriaceae*	*Corynebacterium*	*[Brevibacterium] flavum*	SAMN20145499	GCA_019317305.1
Mycobacteriales	*Corynebacteriaceae*	*Corynebacterium*	*Corynebacterium deserti*	SAMN02950576	GCF_001277995.1
Mycobacteriales	*Corynebacteriaceae*	*Corynebacterium*	*Corynebacterium crudilactis*	SAMN04990137	GCF_001643015.1
Mycobacteriales	*Corynebacteriaceae*	*Corynebacterium*	*Corynebacterium callunae*	SAMN02603058	GCF_000344785.1
Mycobacteriales	*Corynebacteriaceae*	*Corynebacterium*	*Corynebacterium sphenisci*	SAMN02996499	GCF_001941505.1
Mycobacteriales	*Corynebacteriaceae*	*Corynebacterium*	*Corynebacterium aquilae*	SAMN02996496	GCF_001941445.1
Mycobacteriales	*Corynebacteriaceae*	*Corynebacterium*	*Corynebacterium uterequi*	SAMN03480647	GCF_001021065.1
Mycobacteriales	*Corynebacteriaceae*	*Corynebacterium*	*Corynebacterium testudinoris*	SAMN03480629	GCF_001021045.1
Mycobacteriales	*Corynebacteriaceae*	*Corynebacterium*	*Corynebacterium urealyticum*	SAMN16357165	GCF_016127095.1
Mycobacteriales	*Corynebacteriaceae*	*Corynebacterium*	*Corynebacterium atypicum*	SAMN02911287	GCF_000732945.1
Mycobacteriales	*Corynebacteriaceae*	*Corynebacterium*	*Corynebacterium nuruki*	SAMN12496158	GCF_007970465.1
Mycobacteriales	*Corynebacteriaceae*	*Corynebacterium*	*Corynebacterium variabile*	SAMN02603088	GCF_000179395.2
Mycobacteriales	*Corynebacteriaceae*	*Corynebacterium*	*Corynebacterium terpenotabidum*	SAMN02603028	GCF_000418365.1
Mycobacteriales	*Corynebacteriaceae*	*Corynebacterium*	*Corynebacterium provencense*	SAMN08043153	GCF_003209395.1
Mycobacteriales	*Corynebacteriaceae*	*Corynebacterium*	*Corynebacterium incognita*	SAMN15548381	GCF_014217255.1
Mycobacteriales	*Corynebacteriaceae*	*Corynebacterium*	*Corynebacterium occultum*	SAMN13284899	GCF_009734425.1
Mycobacteriales	*Corynebacteriaceae*	*Corynebacterium*	*Corynebacterium uberis*	SAMN22160858	GCF_020614015.1
Mycobacteriales	*Corynebacteriaceae*	*Corynebacterium*	*Corynebacterium bovis*	SAMN16357221	GCF_016128075.1
Mycobacteriales	*Corynebacteriaceae*	*Corynebacterium*	*Corynebacterium amycolatum*	SAMN16357277	GCF_016728725.1
Mycobacteriales	*Corynebacteriaceae*	*Corynebacterium*	*Corynebacterium sp. ATCC 6931*	SAMN02902856	GCF_000755185.1
Mycobacteriales	*Corynebacteriaceae*	*Corynebacterium*	*Corynebacterium jeikeium*	SAMN10163223	GCF_003955985.1
Mycobacteriales	*Corynebacteriaceae*	*Corynebacterium*	*Corynebacterium lactis*	SAMN04012704	GCF_001274895.1
Mycobacteriales	*Corynebacteriaceae*	*Corynebacterium*	*Corynebacterium hindlerae*	SAMN18395664	GCF_017638445.1
Mycobacteriales	*Corynebacteriaceae*	*Corynebacterium*	*Corynebacterium zhongnanshanii*	SAMN15951547	GCF_014490575.1
Mycobacteriales	*Corynebacteriaceae*	*Corynebacterium*	*Corynebacterium anserum*	SAMN13546099	GCF_014262665.1
Mycobacteriales	*Corynebacteriaceae*	*Corynebacterium*	*Corynebacterium kalinowskii*	SAMN13284568	GCF_009734385.1
Mycobacteriales	*Corynebacteriaceae*	*Corynebacterium*	*Corynebacterium urogenitale*	SAMN12924940	GCF_009026825.1
Mycobacteriales	*Corynebacteriaceae*	*Corynebacterium*	*Corynebacterium ammoniagenes*	SAMEA5851036	GCF_902381765.1
Mycobacteriales	*Corynebacteriaceae*	*Corynebacterium*	*Corynebacterium stationis*	SAMN04445726	GCF_001561975.1
Mycobacteriales	*Corynebacteriaceae*	*Corynebacterium*	*Corynebacterium casei*	SAMN03081454	GCF_000550785.1
Mycobacteriales	*Corynebacteriaceae*	*Corynebacterium*	*Corynebacterium glucuronolyticum*	SAMN16357222	GCF_016127395.1
Mycobacteriales	*Corynebacteriaceae*	*Corynebacterium*	*Corynebacterium diphtheriae*	SAMN22365121	GCF_020736385.1
Mycobacteriales	*Corynebacteriaceae*	*Corynebacterium*	*Corynebacterium rouxii*	SAMEA5992727	GCF_902702935.1
Mycobacteriales	*Corynebacteriaceae*	*Corynebacterium*	*Corynebacterium phocae*	SAMN02996498	GCF_001941565.1
Mycobacteriales	*Corynebacteriaceae*	*Corynebacterium*	*Corynebacterium pelargi*	SAMN06041739	GCF_004114895.1
Mycobacteriales	*Corynebacteriaceae*	*Corynebacterium*	*Corynebacterium pseudopelargi*	SAMN08449372	GCF_003814005.1
Mycobacteriales	*Corynebacteriaceae*	*Corynebacterium*	*Corynebacterium gerontici*	SAMN08448852	GCF_003813985.1
Mycobacteriales	*Corynebacteriaceae*	*Corynebacterium*	*Corynebacterium choanae*	SAMN08448940	GCF_003813965.1
Mycobacteriales	*Corynebacteriaceae*	*Corynebacterium*	*Corynebacterium renale*	SAMEA4030736	GCF_900478035.1
Mycobacteriales	*Corynebacteriaceae*	*Corynebacterium*	*Corynebacterium glyciniphilum*	SAMN03081498	GCF_000626675.1
Mycobacteriales	*Corynebacteriaceae*	*Corynebacterium*	*Corynebacterium epidermidicanis*	SAMN03462986	GCF_001021025.1

Mycobacteriales	*Corynebacteriaceae*	*Corynebacterium*	*Corynebacterium mustelae*	SAMN03568800	GCF_001020985.1
Mycobacteriales	***Nocardiaceae***	***Hoyosella***	***Hoyosella sp. YIM 151337***	**SAMN31551484**	**GCF_026041215.1**
Mycobacteriales	*Gordoniaceae*	*Williamsia*	*Williamsia deligens*	SAMN05661029	GCF_024171765.1
Mycobacteriales	*Gordoniaceae*	*Williamsia*	*uncultured Williamsia sp.*	SAMEA112157012	GCA_947494875.1
Mycobacteriales	*Gordoniaceae*	*Williamsia*	*Williamsia sp. CHRR-6*	SAMN19136547	GCF_018474015.1
Mycobacteriales	*Gordoniaceae*	*Williamsia*	*Williamsia sp. DF01-3*	SAMN27352446	GCF_023051145.1
Mycobacteriales	*Gordoniaceae*	*Williamsia*	*Williamsia faeni*	SAMN09064160	GCF_003202005.1
Mycobacteriales	*Nocardiaceae*	*Nocardia*	*Nocardia farcinica*	SAMN09723209	GCF_003367415.1
Mycobacteriales	*Nocardiaceae*	*Nocardia*	*Nocardia sp. FDAARGOS_372*	SAMN07312416	GCF_002554515.1
Mycobacteriales	*Nocardiaceae*	*Nocardia*	*Nocardia shimofusensis*	SAMD00040665	GCF_001613125.1
Mycobacteriales	*Nocardiaceae*	*Nocardia*	*Nocardia aurea*	SAMN08826618	GCF_003123685.1
Mycobacteriales	*Nocardiaceae*	*Nocardia*	*Nocardia noduli*	SAMN18094840	GCF_019038455.1
Mycobacteriales	*Nocardiaceae*	*Nocardia*	*Nocardia sp. NBC_00802*	SAMN30553239	GCF_026341645.1
Mycobacteriales	*Nocardiaceae*	*Nocardia*	*Nocardia salmonicida*	SAMD00018771	GCF_001613085.1
Mycobacteriales	*Nocardiaceae*	*Nocardia*	*Nocardia fluminea*	SAMN04489843	GCF_002846365.1
Mycobacteriales	*Nocardiaceae*	*Nocardia*	*Nocardia alba*	SAMN09074704	GCF_004339125.1
Mycobacteriales	*Nocardiaceae*	*Nocardia*	*Nocardia asteroides*	SAMN23242890	GCF_021183605.1
Mycobacteriales	*Nocardiaceae*	*Nocardia*	*Nocardia mangyaensis*	SAMN06019995	GCF_001886715.1
Mycobacteriales	*Nocardiaceae*	*Nocardia*	*Nocardia rosealba*	SAMN21620047	GCF_020216065.1
Mycobacteriales	*Nocardiaceae*	*Nocardia*	*Nocardia coubleae*	SAMN14517881	GCF_012395945.1
Mycobacteriales	*Nocardiaceae*	*Nocardia*	*Nocardia caishijiensis*	SAMN12250502	GCF_009858255.1
Mycobacteriales	*Nocardiaceae*	*Nocardia*	*Nocardia sp. alder85J*	SAMN20345131	GCF_020176955.2
Mycobacteriales	*Nocardiaceae*	*Nocardia*	*Nocardia aurantia*	SAMN11902338	GCF_009604425.1
Mycobacteriales	*Nocardiaceae*	*Nocardia*	*Nocardia zapadnayensis*	SAMN28693309	GCF_026229585.1
Mycobacteriales	*Nocardiaceae*	*Nocardia*	*Nocardia flavorosea*	SAMN16551420	GCF_015477805.1
Mycobacteriales	*Nocardiaceae*	*Nocardia*	*Nocardia carnea*	SAMD00041818	GCF_000308515.1
Mycobacteriales	*Nocardiaceae*	*Nocardia*	*Nocardia sp. CDC141*	SAMN28906645	GCF_023740755.1
Mycobacteriales	*Nocardiaceae*	*Nocardia*	*Nocardia sp. CDC159*	SAMN28906646	GCF_023740735.1
Mycobacteriales	*Nocardiaceae*	*Nocardia*	*Nocardia pseudobrasiliensis*	SAMN09074773	GCF_003350585.1
Mycobacteriales	*Nocardiaceae*	*Nocardia*	*Nocardia asteroides*	SAMN20888902	GCF_019930625.1
Mycobacteriales	*Nocardiaceae*	*Nocardia*	*Nocardia gipuzkoensis*	SAMN23242888	GCF_021183645.1
Mycobacteriales	*Nocardiaceae*	*Nocardia*	*Nocardia abscessus*	SAMN22365125	GCF_020731305.1
Mycobacteriales	*Nocardiaceae*	*Nocardia*	*Nocardia araoensis*	SAMD00041768	GCF_000308435.1
Mycobacteriales	*Nocardiaceae*	*Nocardia*	*Nocardia iowensis*	SAMN20062579	GCF_019222765.1
Mycobacteriales	*Nocardiaceae*	*Nocardia*	*Nocardia suismassiliense*	SAMEA104566999	GCF_900269665.1
Mycobacteriales	*Nocardiaceae*	*Nocardia*	*Nocardia sp. CS682*	SAMN09280253	GCF_004634405.1
Mycobacteriales	*Nocardiaceae*	*Nocardia*	*Nocardia brasiliensis*	SAMN06173365	GCF_002209125.2
Mycobacteriales	*Nocardiaceae*	*Nocardia*	*Nocardia vulneris*	SAMD00040670	GCF_001613425.1
Mycobacteriales	*Nocardiaceae*	*Nocardia*	*Nocardia altamirensis*	SAMD00018774	GCF_001612685.1
Mycobacteriales	*Nocardiaceae*	*Nocardia*	*Nocardia bhagyanarayanae*	SAMN11511976	GCF_006716565.1
Mycobacteriales	*Nocardiaceae*	*Nocardia*	*Nocardia goodfellowii*	SAMN05878021	GCF_017875645.1
Mycobacteriales	*Nocardiaceae*	*Nocardia*	*Nocardia bovistercoris*	SAMN16707683	GCF_015674855.1
Mycobacteriales	*Nocardiaceae*	*Nocardia*	*Nocardia sp. DSM 111726*	SAMN22568590	GCF_020733635.1
Mycobacteriales	*Nocardiaceae*	*Nocardia*	*Nocardia africana*	SAMN22365126	GCF_020731565.1
Mycobacteriales	*Nocardiaceae*	*Nocardia*	*Nocardia nova*	SAMN08458271	GCF_002933535.1
Mycobacteriales	*Nocardiaceae*	*Nocardia*	*Nocardia veterana*	SAMN14517858	GCF_012396305.1
Mycobacteriales	*Nocardiaceae*	*Nocardia*	*Nocardia vermiculata*	SAMN14517876	GCF_012396005.1
Mycobacteriales	*Nocardiaceae*	*Nocardia*	*Nocardia sp. ncl2*	SAMN18094565	GCF_019038435.1
Mycobacteriales	*Nocardiaceae*	*Nocardia*	*Nocardia yamanashiensis*	SAMN23242892	GCF_021183565.1
Mycobacteriales	*Nocardiaceae*	*Nocardia*	*Nocardia inohanensis*	SAMD00040655	GCF_001612945.1
Mycobacteriales	*Nocardiaceae*	*Nocardia*	*Nocardia tengchongensis*	SAMN19031525	GCF_018362975.1
Mycobacteriales	*Nocardiaceae*	*Nocardia*	*Nocardia seriolae*	SAMN12164966	GCF_009712575.1
Mycobacteriales	*Nocardiaceae*	*Nocardia*	*Nocardia yunnanensis*	SAMN10135935	GCF_003626895.1
Mycobacteriales	*Nocardiaceae*	*Nocardia*	*Nocardia acidivorans*	SAMD00040643	GCF_001625085.1
Mycobacteriales	*Nocardiaceae*	*Nocardia*	*Nocardia crassostreae*	SAMD00040648	GCF_001613405.1
Mycobacteriales	*Nocardiaceae*	*Nocardia*	*Nocardia jejuensis*	SAMD00018769	GCF_001613145.1
Mycobacteriales	*Nocardiaceae*	*Nocardia*	*Nocardia wallacei*	SAMD00238642	GCF_014466955.1
Mycobacteriales	*Nocardiaceae*	*Nocardia*	*Nocardia stercoris*	SAMN10284878	GCF_003696265.1

Mycobacteriales	*Nocardiaceae*	*Nocardia*	*Nocardia panacis*	SAMN10089539	GCF_003598715.1
Mycobacteriales	*Nocardiaceae*	*Nocardia*	*Nocardia mexicana*	SAMN09074698	GCF_003350525.1
Mycobacteriales	*Nocardiaceae*	*Nocardia*	*Nocardia cyriacigeorgica*	SAMN08458266	GCF_002949635.1
Mycobacteriales	*Nocardiaceae*	*Nocardia*	*Nocardia cyriacigeorgica*	SAMN08458267	GCF_002933455.1
Mycobacteriales	*Nocardiaceae*	*Nocardia*	*Nocardia terpenica*	SAMN04426989	GCF_001625105.1
Mycobacteriales	*Nocardiaceae*	*Nocardia*	*Nocardia harenae*	SAMD00040653	GCF_001612885.1
Mycobacteriales	*Nocardiaceae*	*Nocardia*	*Nocardia takedensis*	SAMD00041777	GCF_000308695.1
Mycobacteriales	*Nocardiaceae*	*Nocardia*	*Nocardia sp. BMG51109*	SAMN02584923	GCF_000526215.1
Mycobacteriales	*Nocardiaceae*	*Nocardia*	*Nocardia globerula*	SAMN14517869	GCA_012843125.1
Mycobacteriales	*Nocardiaceae*	*Rhodococcus*	*Rhodococcus globerulus*	SAMN20011299	GCF_019334125.1
Mycobacteriales	*Nocardiaceae*	*Rhodococcus*	*Rhodococcus qingshengii*	SAMD00491760	GCF_026074015.1
Mycobacteriales	*Nocardiaceae*	*Rhodococcus*	*Rhodococcus erythropolis*	SAMD00119030	GCF_024349805.1
Mycobacteriales	*Nocardiaceae*	*Rhodococcus*	*Rhodococcus qingshengii*	SAMN04589880	GCF_001623435.1
Mycobacteriales	*Nocardiaceae*	*Rhodococcus*	*Rhodococcus baikonurensis*	SAMD00016817	GCF_001311605.1
Mycobacteriales	*Nocardiaceae*	*Rhodococcus*	*Rhodococcus erythropolis*	SAMD00143522	GCF_003990875.1
Mycobacteriales	*Nocardiaceae*	*Rhodococcus*	*Rhodococcus qingshengii*	SAMN07414823	GCF_002744595.1
Mycobacteriales	*Nocardiaceae*	*Rhodococcus*	*Rhodococcus erythropolis*	SAMN10077188	GCF_021497645.1
Mycobacteriales	*Nocardiaceae*	*Rhodococcus*	*Rhodococcus erythropolis*	SAMN18298927	GCF_018615075.1
Mycobacteriales	*Nocardiaceae*	*Rhodococcus*	*Rhodococcus sp. USK13*	SAMN17526040	GCF_018343855.1
Mycobacteriales	*Nocardiaceae*	*Rhodococcus*	*Rhodococcus opacus*	SAMN20605963	GCF_020542785.1
Mycobacteriales	*Nocardiaceae*	*Rhodococcus*	*Rhodococcus sp. IEGM 248*	SAMN13936297	GCF_010436075.1
Mycobacteriales	*Nocardiaceae*	*Rhodococcus*	*Rhodococcus sp. WS4*	SAMN10354867	GCF_006543605.1
Mycobacteriales	*Nocardiaceae*	*Rhodococcus*	*Rhodococcus jostii*	SAMD00046778	GCF_001894825.1
Mycobacteriales	*Nocardiaceae*	*Rhodococcus*	*Rhodococcus sp. 4CII*	SAMN15495701	GCF_014256275.1
Mycobacteriales	*Nocardiaceae*	*Rhodococcus*	*Rhodococcus opacus*	SAMD00060964	GCF_000010805.1
Mycobacteriales	*Nocardiaceae*	*Rhodococcus*	*Rhodococcus sp. T7*	SAMN13717632	GCF_009872365.1
Mycobacteriales	*Nocardiaceae*	*Rhodococcus*	*Rhodococcus pseudokoreensis*	SAMN17840530	GCF_017068395.1
Mycobacteriales	*Nocardiaceae*	*Rhodococcus*	*Rhodococcus wratislaviensis*	SAMD00148910	GCF_003851765.1
Mycobacteriales	*Nocardiaceae*	*Rhodococcus*	*Rhodococcus sp. T2V*	SAMN33620769	GCA_029269925.1
Mycobacteriales	*Nocardiaceae*	*Rhodococcus*	*Rhodococcus gordoniae*	SAMN29637735	GCF_024362245.1
Mycobacteriales	*Nocardiaceae*	*Rhodococcus*	*Rhodococcus sp. B50*	SAMN13483355	GCF_013602415.1
Mycobacteriales	*Nocardiaceae*	*Rhodococcus*	*Rhodococcus pyridinivorans*	SAMN23420020	GCF_021026105.1
Mycobacteriales	*Nocardiaceae*	*Rhodococcus*	*Rhodococcus pyridinivorans*	SAMN31691423	GCF_026802115.1
Mycobacteriales	*Nocardiaceae*	*Rhodococcus*	*Rhodococcus sp. RDE2*	SAMD00412678	GCF_020885655.1
Mycobacteriales	*Nocardiaceae*	*Rhodococcus*	*Rhodococcus rhodochrous*	SAMN13685868	GCF_009831075.1
Mycobacteriales	*Nocardiaceae*	*Rhodococcus*	*Rhodococcus sp. CH91*	SAMN24906810	GCF_022554085.1
Mycobacteriales	*Nocardiaceae*	*Rhodococcus*	*Rhodococcus sp. Z13*	SAMN31157772	GCF_025837095.1
Mycobacteriales	*Nocardiaceae*	*Rhodococcus*	*Rhodococcus zopfii*	SAMN11633062	GCF_006148895.1
Mycobacteriales	*Nocardiaceae*	*Rhodococcus*	*Rhodococcus phenolicus*	SAMN04357313	GCF_001646785.1
Mycobacteriales	*Nocardiaceae*	*Rhodococcus*	*Rhodococcus sp. 14C212*	SAMN14142454	GCF_011058195.1
Mycobacteriales	*Nocardiaceae*	*Rhodococcus*	*Rhodococcus sp. YH1*	SAMN13707389	GCF_009939605.1
Mycobacteriales	*Nocardiaceae*	*Rhodococcus*	*Rhodococcus aetherivorans*	SAMN03486836	GCF_000982715.1
Mycobacteriales	*Nocardiaceae*	*Rhodococcus*	*Rhodococcus ruber*	SAMN23420027	GCF_021025995.1
Mycobacteriales	*Nocardiaceae*	*Rhodococcus*	*Rhodococcus yananensis*	SAMN21894551	GCF_020515525.1
Mycobacteriales	*Nocardiaceae*	*Rhodococcus*	*Rhodococcus triatomae*	SAMN14089706	GCF_014217785.1
Mycobacteriales	*Nocardiaceae*	*Rhodococcus*	*Rhodococcus coprophilus*	SAMEA4412684	GCF_900478115.1
Mycobacteriales	*Nocardiaceae*	*Rhodococcus*	*Rhodococcus sp. SMB37*	SAMN10872458	GCF_004345605.1
Mycobacteriales	*Nocardiaceae*	*Rhodococcus*	*Rhodococcus rhodnii*	SAMN09739484	GCF_008011915.1
Mycobacteriales	*Nocardiaceae*	*Rhodococcus*	*Rhodococcus spelaei*	SAMN12158049	GCF_006704125.1
Mycobacteriales	*Nocardiaceae*	*Rhodococcus*	*Rhodococcus sp. MTM3W5.2*	SAMN06160711	GCF_001984015.1
Mycobacteriales	*Nocardiaceae*	*Rhodococcus*	*Rhodococcus oryzae*	SAMN11475379	GCF_005049235.1
Mycobacteriales	*Nocardiaceae*	*Rhodococcus*	*Rhodococcus maanshanensis*	SAMN05444583	GCF_900109405.1
Mycobacteriales	*Nocardiaceae*	*Rhodococcus*	*Rhodococcus tukisamuensis*	SAMN05444580	GCF_900101735.1
Mycobacteriales	*Nocardiaceae*	*Rhodococcus*	*Rhodococcus xishaensis*	SAMN10395176	GCF_004011825.1
Mycobacteriales	*Nocardiaceae*	*Rhodococcus*	*Rhodococcus spongiicola*	SAMN10395175	GCF_004011835.1
Mycobacteriales	*Nocardiaceae*	*Rhodococcus*	*Prescottella equi*	SAMN25558261	GCF_022691685.1
Mycobacteriales	*Nocardiaceae*	*Rhodococcus*	*Prescottella equi*	SAMN25558260	GCF_022691665.1
Mycobacteriales	*Nocardiaceae*	*Rhodococcus*	*Prescottella equi*	SAMN13450482	GCF_016025875.1

Mycobacteriales	*Nocardiaceae*	*Rhodococcus*	*Prescottella equi*	SAMN13391757	GCA_012443715.1
Mycobacteriales	*Nocardiaceae*	*Rhodococcus*	*Prescottella agglutinans*	SAMN10395197	GCF_004011865.1
Mycobacteriales	*Nocardiaceae*	*Rhodococcus*	*Prescottella defluvii*	SAMN02910065	GCF_000738775.1
Mycobacteriales	*Nocardiaceae*	*Rhodococcus*	*Rhodococcus sp. W8901*	SAMN15150009	GCF_013348805.1
Mycobacteriales	*Nocardiaceae*	*Rhodococcus*	*Rhodococcus sp. SGAir0479*	SAMN11388438	GCF_005484805.1
Mycobacteriales	*Nocardiaceae*	*Rhodococcus*	*Rhodococcus sp. OK519*	SAMN08777571	GCF_003051005.1
Mycobacteriales	*Nocardiaceae*	*Rhodococcus*	*Rhodococcus sp. Q*	SAMN08712319	GCF_005503035.1
Mycobacteriales	*Nocardiaceae*	*Rhodococcus*	*Rhodococcus triatomae*	SAMN02469993	GCF_000341795.1
Mycobacteriales	*Nocardiaceae*	*Rhodococcus*	*Rhodococcus sp. JG-3*	SAMN02441111	GCF_000482405.1
Mycobacteriales	*Nocardiaceae*	*Rhodococcus*	*Rhodococcus fascians*	SAMN15000776	GCF_019784225.1
Mycobacteriales	*Nocardiaceae*	*Rhodococcus*	*Rhodococcus fascians*	SAMN15000810	GCF_019783545.1
Mycobacteriales	*Nocardiaceae*	*Rhodococcus*	*Rhodococcus fascians*	SAMN15000814	GCF_019783505.1
Mycobacteriales	*Nocardiaceae*	*Rhodococcus*	*Rhodococcus fascians*	SAMN15000812	GCF_019783485.1
Mycobacteriales	*Nocardiaceae*	*Rhodococcus*	*Rhodococcus fascians*	SAMN15000829	GCF_019782825.1
Mycobacteriales	*Nocardiaceae*	*Rhodococcus*	*Rhodococcus fascians*	SAMN15000867	GCF_019782455.1
Mycobacteriales	*Nocardiaceae*	*Rhodococcus*	*Rhodococcus fascians*	SAMEA7892365	GCF_918697885.1
Mycobacteriales	*Nocardiaceae*	*Rhodococcus*	*Rhodococcus fascians*	SAMN07414744	GCF_002259505.1
Mycobacteriales	*Nocardiaceae*	*Rhodococcus*	*Rhodococcus fascians*	SAMN07376390	GCF_002259165.1
Mycobacteriales	*Nocardiaceae*	*Rhodococcus*	*Rhodococcus sp. 05-2256-B3*	SAMN07376360	GCF_002259065.1
Mycobacteriales	*Nocardiaceae*	*Rhodococcus*	*Rhodococcus sp. 05-2255-1e*	SAMN07376365	GCF_002259025.1
Mycobacteriales	*Nocardiaceae*	*Rhodococcus*	*Rhodococcus sp. 06-1460-1B*	SAMN07376371	GCF_002258475.1
Mycobacteriales	*Nocardiaceae*	*Rhodococcus*	*Rhodococcus sp. 1R11*	SAMN11249662	GCF_004563845.1
Mycobacteriales	*Nocardiaceae*	*Rhodococcus*	*Rhodococcus fascians*	SAMD00046773	GCF_001894785.1
Mycobacteriales	*Nocardiaceae*	*Rhodococcus*	*Rhodococcus fascians*	SAMN04521220	GCF_001620305.1
Mycobacteriales	*Nocardiaceae*	*Rhodococcus*	*Rhodococcus sp. 06-235-1A*	SAMN07376378	GCF_002258785.1
Mycobacteriales	*Nocardiaceae*	*Rhodococcus*	*Rhodococcus sp. 14-2483-1-2*	SAMN07414572	GCF_002259405.1
Mycobacteriales	*Nocardiaceae*	*Rhodococcus*	*Rhodococcus sp. 14-2496-1d*	SAMN07414573	GCF_002259365.1
Mycobacteriales	*Nocardiaceae*	*Rhodococcus*	*Rhodococcus fascians*	SAMN15000868	GCF_019782385.1
Mycobacteriales	*Nocardiaceae*	*Rhodococcus*	*Rhodococcus sp. KRD162*	SAMN14679900	GCF_017168335.1
Mycobacteriales	*Nocardiaceae*	*Rhodococcus*	*Rhodococcus fascians*	SAMN02569991	GCF_000760935.2
Mycobacteriales	*Nocardiaceae*	*Rhodococcus*	*Rhodococcus fascians*	SAMN02569990	GCF_000760905.2
Mycobacteriales	*Nocardiaceae*	*Rhodococcus*	*Rhodococcus sp. 06-412-2B*	SAMN07376382	GCF_002258385.1
Mycobacteriales	*Nocardiaceae*	*Rhodococcus*	*Rhodococcus sp. IEGM 1401*	SAMN32240013	GCF_027270635.1
Mycobacteriales	*Nocardiaceae*	*Rhodococcus*	*Rhodococcus sp. 14-1411-2a*	SAMN07414568	GCF_002259485.1
Mycobacteriales	*Nocardiaceae*	*Rhodococcus*	*Rhodococcus sp. Leaf278*	SAMN04151709	GCF_001426085.1
Mycobacteriales	*Nocardiaceae*	*Rhodococcus*	*Rhodococcus sp. SBT000017*	SAMN04495211	GCF_003688915.1
Mycobacteriales	*Nocardiaceae*	*Rhodococcus*	*Rhodococcus kyotonensis*	SAMN05421642	GCF_900188125.1
Mycobacteriales	*Nocardiaceae*	*Rhodococcus*	*Rhodococcus sp. 14-2470-1b*	SAMN07414570	GCF_002259415.1
Mycobacteriales	*Nocardiaceae*	*Rhodococcus*	*Rhodococcus cavernicola*	SAMN12303457	GCF_008297975.1
Mycobacteriales	*Nocardiaceae*	*Rhodococcus*	*Rhodococcus yunnanensis*	SAMN32218586	GCF_027213855.1
Mycobacteriales	*Nocardiaceae*	*Rhodococcus*	*Rhodococcus sp. Eu-32*	SAMN09240609	GCF_003336925.1
Mycobacteriales	*Nocardiaceae*	*Rhodococcus*	*Rhodococcus yunnanensis*	SAMD00046770	GCF_001895005.1
Mycobacteriales	*Tsukamurellaceae*	*Tsukamurella*	*Tsukamurella sp. M9C*	SAMN21529074	GCF_020169415.1
Mycobacteriales	*Tsukamurellaceae*	*Tsukamurella*	*Tsukamurella tyrosinosolvens*	SAMN04157994	GCF_002869785.1
Mycobacteriales	*Tsukamurellaceae*	*Tsukamurella*	*Tsukamurella tyrosinosolvens*	SAMN04479211	GCF_001623895.1
Mycobacteriales	*Tsukamurellaceae*	*Tsukamurella*	*Tsukamurella paurometabola*	SAMN22091655	GCF_020735845.1
Mycobacteriales	*Tsukamurellaceae*	*Tsukamurella*	*Tsukamurella paurometabola*	SAMN18928733	GCF_018335505.1
Mycobacteriales	*Tsukamurellaceae*	*Tsukamurella*	*Tsukamurella columbiensis*	SAMN14517855	GCF_012843135.1
Mycobacteriales	*Tsukamurellaceae*	*Tsukamurella*	*Tsukamurella pulmonis*	SAMD00434442	GCF_022179485.1
Mycobacteriales	*Tsukamurellaceae*	*Tsukamurella*	*Tsukamurella asaccharolytica*	SAMN12158046	GCF_007858435.1
Mycobacteriales	*Tsukamurellaceae*	*Tsukamurella*	*Tsukamurella sp. PLM1*	SAMN27015329	GCF_023168325.1
Mycobacteriales	*Tsukamurellaceae*	*Tsukamurella*	*Tsukamurella spumae*	SAMN14517875	GCF_012396015.1
Mycobacteriales	*Tsukamurellaceae*	*Tsukamurella*	*Tsukamurella sp. 1534*	SAMEA2272095	GCF_000312385.1
Mycobacteriales	*Tsukamurellaceae*	*Tsukamurella*	*Tsukamurella sp. TY48*	SAMD00250050	GCF_019656655.1
Mycobacteriales	*Gordoniaceae*	*Gordonia*	*Gordonia alkanivorans*	SAMN08565628	GCF_004011905.1
Mycobacteriales	*Gordoniaceae*	*Gordonia*	*Gordonia ajococcus*	SAMN14731745	GCF_012974285.1
Mycobacteriales	*Gordoniaceae*	*Gordonia*	*Gordonia jinghuaiqii*	SAMN15594914	GCF_014041935.1
Mycobacteriales	*Gordoniaceae*	*Gordonia*	*Gordonia bronchialis*	SAMN22365123	GCF_020731255.1

Mycobacteriales	*Gordoniaceae*	*Gordonia*	*Gordonia bronchialis*	SAMN11056391	GCF_009730435.1
Mycobacteriales	*Gordoniaceae*	*Gordonia*	*Gordonia sp. ABSL49_1*	SAMN26237318	GCF_022513585.1
Mycobacteriales	*Gordoniaceae*	*Gordonia*	*Gordonia pseudamarae*	SAMN13182582	GCF_025273675.1
Mycobacteriales	*Gordoniaceae*	*Gordonia*	*Gordonia amarae*	SAMN13182585	GCF_009914515.1
Mycobacteriales	*Gordoniaceae*	*Gordonia*	*Gordonia hankookensis*	SAMN16120623	GCF_014673215.1
Mycobacteriales	*Gordoniaceae*	*Gordonia*	*Gordonia sp. SL306*	SAMN31669892	GCF_026625785.1
Mycobacteriales	*Gordoniaceae*	*Gordonia*	*Gordonia sp. SID5947*	SAMN13630882	GCF_009862785.1
Mycobacteriales	*Gordoniaceae*	*Gordonia*	*Gordonia sedimidis*	SAMN10642655	GCF_004193205.1
Mycobacteriales	*Gordoniaceae*	*Gordonia*	*Gordonia polyisoprenivorans*	SAMN02256401	GCF_000385355.1
Mycobacteriales	*Gordoniaceae*	*Gordonia*	*Gordonia jacobaea*	SAMN31808702	GCF_027659065.1
Mycobacteriales	*Gordoniaceae*	*Gordonia*	*Gordonia sp. 852002-50395_SCH5434458*	SAMN04696146	GCF_001665905.1
Mycobacteriales	*Gordoniaceae*	*Gordonia*	*Gordonia crocea*	SAMD00170765	GCF_009932435.1
Mycobacteriales	*Gordoniaceae*	*Gordonia*	*Gordonia sp. X0973*	SAMN15150008	GCF_013348785.1
Mycobacteriales	*Gordoniaceae*	*Gordonia*	*Gordonia alkaliphila*	SAMN27187103	GCF_023017165.1
Mycobacteriales	*Gordoniaceae*	*Gordonia*	*Gordonia hirsuta*	SAMD00041827	GCF_000333015.1
Mycobacteriales	*Gordoniaceae*	*Gordonia*	*Gordonia liuliyuniae*	SAMN25118198	GCF_021739025.1
Mycobacteriales	*Gordoniaceae*	*Gordonia*	*Gordonia liuliyuniae*	SAMN25118129	GCF_021738805.1
Mycobacteriales	*Gordoniaceae*	*Gordonia*	*Gordonia humi*	SAMN05877986	GCF_014197435.1
Mycobacteriales	*Gordoniaceae*	*Gordonia*	*Gordonia spumicola*	SAMD00170766	GCF_009932475.1
Mycobacteriales	*Gordoniaceae*	*Gordonia*	*Gordonia malaquae*	SAMN04488550	GCF_900105435.1
Mycobacteriales	*Nocardiaceae*	*Antrihabitans*	*Antrihabitans stalagmiti*	SAMN17150779	GCF_016482825.1
Mycobacteriales	*Nocardiaceae*	*Antrihabitans*	*Antrihabitans sp. YC2-6*	SAMN17150780	GCF_016481275.1
Mycobacteriales	***Nocardiaceae***	***Antrihabitans***	***Antrihabitans stalactiti***	**SAMN11867825**	**GCF_012932915.1**
Mycobacteriales	*Nocardiaceae*	*Skermania*	*Skermania piniformis*	SAMN20079133	GCF_019285775.1
Mycobacteriales	***Nocardiaceae***	***Tomitella***	***Tomitella fengzijianii***	**SAMN23497867**	**GCF_023162105.1**
Mycobacteriales	*Nocardiaceae*	*Tomitella*	*Tomitella gaofuii*	SAMN23497866	GCF_023162085.1
Mycobacteriales	*Nocardiaceae*	*Tomitella*	*Tomitella gaofuii*	SAMN15676248	GCF_014121175.1
Mycobacteriales	*Nocardiaceae*	*Tomitella*	*Tomitella cavernea*	SAMN17109521	GCF_016599145.1
Mycobacteriales	*Nocardiaceae*	*Tomitella*	*Tomitella biformata*	SAMN17109562	GCF_016599245.1
Mycobacteriales	*Nocardiaceae*	*Aldersonia*	*Aldersonia kunmingensis*	SAMN04357316	GCF_001646865.1
Mycobacteriales	*Gordoniaceae*	*Gordonia*	*Gordonia sp. OPL2*	SAMN10354926	GCF_003797825.1
Mycobacteriales	***Tsukamurellaceae***	***Tsukamurella***	***Tsukamurella sp. 8J***	**SAMN33554932**	**GCF_029167405.1**
Microbacterium	*Microbacteriaceae*	*Microbacterium*	*Microbacterium sp. AZCO*	SAMN41107758	GCF_039614715.1
Microbacterium	*Microbacteriaceae*	*Microbacterium*	*Microbacterium enclense*	SAMN32653126	GCA_038182865.1
Microbacterium	*Microbacteriaceae*	*Microbacterium*	*Microbacterium limosum*	SAMN37714333	GCF_036324365.1
Microbacterium	*Microbacteriaceae*	*Microbacterium*	*Microbacterium plantarum*	SAMN38810176	GCF_035231185.1

Table S3. Cluster parameters of *Mycobacterium* genomes.

Distance calculation method	Medoid genome or genomes selected for the best projection of clusters		Average distances		The highest intracluster distance	The lowest intercluster distance	Gap	Interception
			intracluster	intercluster				
**all-by-all**								
ANI			0.205	0.240	0.250	0.211	-0.04	9.4%
GGDC-1			0.809	0.868	0.873	0.847	-0.026	8.6%
GGDC-2			0.792	0.799	0.826	0.596	-0.230	73.2%
MASH			0.208	0.274	0.310	0.204	-0.106	9.8%
MLSA			0.159	0.243	0.321	0.175	-0.145	4.6%
MPSA			0.110	0.201	0.192	0.126	-0.065	2.9%
AAI			0.266	0.385	0.336	0.344	**0.008**	0.0%
**Medoids method**								
ANI	GCF_009741445.1	*M. avium*	0.193	0.237	0.222	0.220	-0.002	2.4%
GGDC-1	GCF_001667275.1	*M.* sp. E2989	0.755	0.868	0.862	0.859	-0.003	2.3%
GGDC-2	GCA_026005275.1	*M.* sp. NGTWS1803	0.768	0.802	0.791	0.781	-0.010	7.2%
MASH	GCF_009741445.1	*M. avium*	0.188	0.268	0.243	0.226	-0.017	6.1%
MLSA	GCF_900161855.1	*M. colombiense*	0.139	0.230	0.255	0.183	-0.072	0.4%
MPSA	GCF_001722425.1	*M. porcinum*	0.092	0.188	0.140	0.127	-0.013	0.8%
AAI	GCF_002086215.1	*M. heidelbergense*	0.244	0.382	0.321	0.353	**0.031**	0.0%
**LDA method**								
ANI	GCF_009192875.1<br>GCF_009939605.1	*M. phlei*<br>*Rhodococcus* sp. YH1	0.092	0.114	0.100	0.108	**0.008**	0.0%
GGDC-1	GCF_003402475.1<br>GCF_004011825.1	*M.* sp. MFM001<br>*Rhodococcus xishaensis*	0.401	0.440	0.428	0.432	**0.005**	0.0%
GGDC-2	GCA_026005275.1<br>GCF_009872365.1	*M.* sp. NGTWS1803<br>*Rhodococcus sp. T7*	0.380	0.408	0.389	0.386	**-0.003**	1.6%
MASH	GCF_032883035.1<br>GCF_005049235.1	*M.* sp. 21AC1<br>*Rhodococcus oryzae*	0.114	0.153	0.127	0.140	**0.013**	0.0%
MLSA	GCF_036409095.1<br>GCF_002993285.1	*M.* sp. SCH5140682<br>*Gordonia iterans*	0.082	0.137	0.096	0.113	**0.016**	0.0%
MPSA	GCF_020172685.1<br>GCF_004006015.1	*M.* sp. MYC340<br>*Rhodococcus sp. X156*	0.049	0.105	0.065	0.091	**0.026**	0.0%
AAI	GCF_003201655.1<br>GCF_000308515.1	*M.* sp. GAS496<br>*Nocardia carnea*	0.129	0.200	0.160	0.186	**0.026**	0.0%
